# Synthesis of yellow and red fluorescent 1,3a,6a-triazapentalenes and the theoretical investigation of their optical properties†
†Electronic supplementary information (ESI) available: the experimental details for the synthesis of the triazapentalenes and the fluorescent cell staining, the absorption and fluorescence spectra, and the ^1^H and ^13^C NMR spectra. Also given are the molecular orbitals, the natural charges, the dipole moments, and the Cartesian coordinates of the triazapentalenes (**1a**, **1b**, **1g**, **1e**, and **1f**). See DOI: 10.1039/c4sc02780a



**DOI:** 10.1039/c4sc02780a

**Published:** 2014-10-23

**Authors:** Kosuke Namba, Ayumi Osawa, Akira Nakayama, Akane Mera, Fumi Tano, Yoshiro Chuman, Eri Sakuda, Tetsuya Taketsugu, Kazuyasu Sakaguchi, Noboru Kitamura, Keiji Tanino

**Affiliations:** a Department of Pharmaceutical Science , The University of Tokushima , 1-78 Shomachi , Tokushima 770-8505 , Japan . Email: namba@tokushima-u.ac.jp; b Graduate School of Chemical Sciences and Engineering , Hokkaido University , Sapporo 060-0810 , Japan; c Department of Chemistry , Faculty of Science , Hokkaido University , Kita-ku , Sapporo 060-0810 , Japan; d Catalysis Research Center , Hokkaido University , Sapporo 001-0021 , Japan

## Abstract

To expand the originally developed fluorescent 1,3a,6a-triazapentalenes as fluorescent labelling reagents, the fluorescence wavelength of the 1,3a,6a-triazapentalenes was extended to the red color region. Based on the noteworthy correlation of the fluorescence wavelength with the inductive effect of the 2-substituent, electron-deficient 2-(2-cyano-4-methoxycarbonylphenyl)-1,3a,6a-triazapentalene and 2-(2,6-dicyano-4-methoxycarbonylphenyl)-1,3a,6a-triazapentalene were synthesized. The former exhibited yellow fluorescence and the latter exhibited red fluorescence, and both compounds exhibited large Stokes shifts, and the 1,3a,6a-triazapentalene system enabled the same fluorescent chromophore to cover the entire region of visible wavelengths. The potential applications of the 1,3a,6a-triazapentalenes as fluorescent probes in the fields of the life sciences were investigated, and the 1,3a,6a-triazapentalene system was clearly proven to be useful as a fluorescent reagent for live cell imaging. Quantum chemical calculations were performed to investigate the optical properties of the 1,3a,6a-triazapentalenes. These calculations revealed that the excitation involves a significant charge-transfer from the 1,3a,6a-triazapentalene skeleton to the 2-substituent. The calculated absorption and fluorescence wavelengths showed a good correlation with the experimental ones, and thus the system could enable the theoretical design of substituents with the desired optical properties.

## Abstract 2

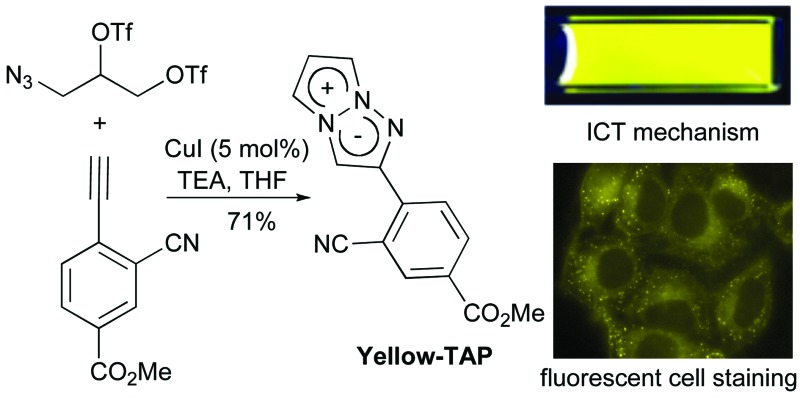
To expand the function of the fluorescent 1,3a,6a-triazapentalenes as labelling reagents, their fluorescence wavelength was extended to the red color region.

## Introduction

Fluorescent organic molecules are an important class of compounds in modern science and technology, and are widely used as biological imaging probes, sensors, lasers, and in light-emitting devices.^1^ Thus, the development of useful fluorescent organic molecules is crucial for the advancement of many industries, and has been a subject of intensive research.^2^ In particular, small fluorescent organic molecules have attracted great attention in the field of chemical biology, because the visualization of biologically active small compounds by introducing fluorophores is one of the most useful ways for studying their mechanism.^3^ However, several key improvements are needed for the commonly used fluorescent molecules. The most highly fluorescent molecules possess a relatively large molecular size depending on the target bioactive compounds, and the fluorescence-labelled molecules sometimes lose their activity as a result of the structural modifications. Furthermore, often the methods used to synthesize them do not allow for the design of systems whose luminescence properties span a wide range of wavelengths. As a potential fluorescent chromophore to overcome the above problems, we have recently discovered that a 1,3a,6a-triazapentalene skeleton without an additional fused ring system is a compact and highly fluorescent chromophore.^4,5^ In contrast, benzotriazapentalene as an aryl-fused ring system exhibits almost no fluorescence (*Φ*_F_ < 0.001),^6^ and the various related analogues of the aryl-fused 1,3a,6a-triazapentalenes^7^ have not been reported to have noteworthy fluorescence properties. The limited synthesis of 1,3a,6a-triazapentalenes without an aryl-fused system^8^ might be the main reason that they have been previously unrecognized as excellent fluorescent chromophores until our finding.

**Scheme 1:**
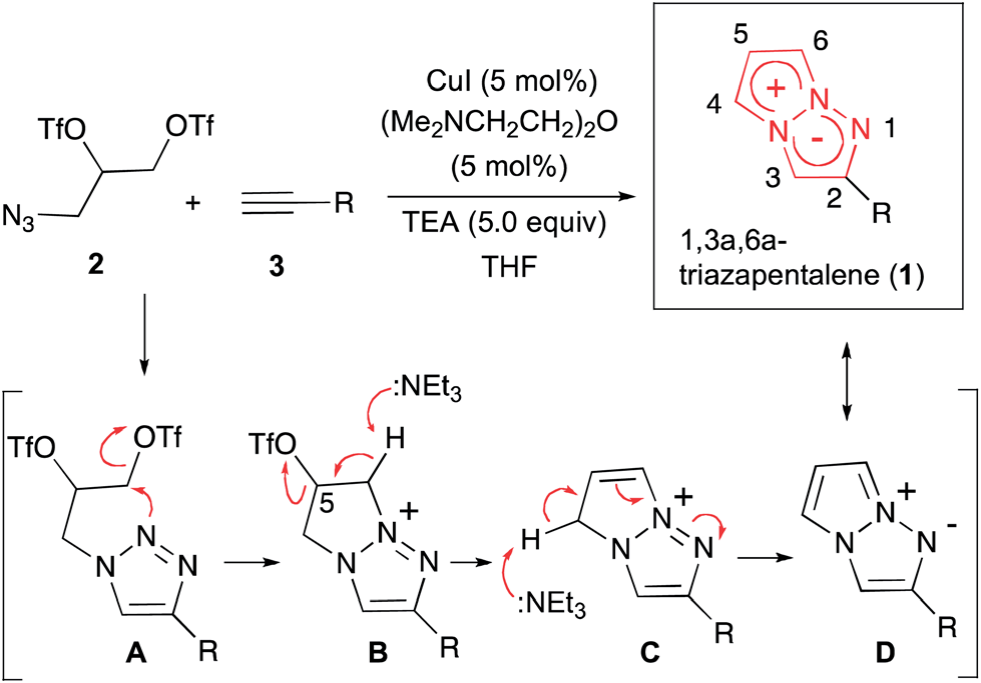
Single step synthesis of the 1,3a,6a-triazapentalenes (**1**).

The construction of the 1,3a,6a-triazapentalene skeleton without an aryl-fused ring system was recently established in our laboratory, and the 1,3a,6a-triazapentalenes were readily prepared by the click-cyclization–aromatization cascade reaction of various alkynes with the azide **2** possessing two triflates at the *C*2 and *C*3 positions (Scheme 1).^4^ The click reaction of azide **2** with the alkynes produced triazole **A**, which underwent cyclization to give triazolium ion **B**. In the presence of triethylamine, the intermediate **B** was subsequently converted to triazapentalene **1** by a sequential reaction of E2 elimination and deprotonation (Scheme 1). This cascade reaction was confirmed to be applicable to a wide range of alkynes, and the easy access to the various 1,3a,6a-triazapentalenes was enabled. Furthermore, the 5,5-dimethoxy analog of **B** was found to be stable enough for isolation, and a strong base was necessary for the elimination of the methoxy group to give 5-methoxy-1,3a,6a-triazapentalenes. This method was applicable to the one-pot synthesis of the various 2,5-disubstituted-1,3a,6a-triazapentalenes.^9^ Although the 1,3a,6a-triazapentalenes are composed of a zwitter ion, the polarities and the electrical charges are neutralised due to the resonance stabilization of the aromatic compounds and so they are easily manipulated.

**Fig. 1:**
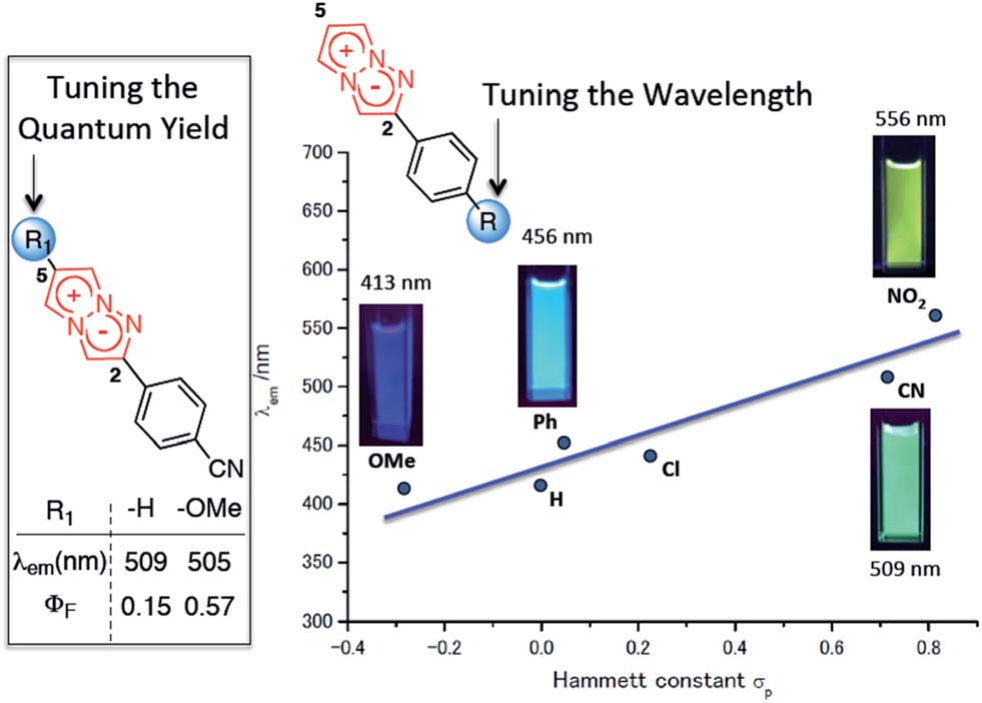
Substitution effect on the fluorescence properties of the 1,3a,6a-triazapentalenes. The values were determined in deaerated dichloromethane.

The 1,3a,6a-triazapentalenes exhibit not only intense fluorescence but also various interesting fluorescence properties such as an extremely large Stokes shift (Stokes shift exceeding 100 nm)^10^ and large positive fluorescence solvatochromism. More interestingly, the 1,3a,6a-triazapentalenes as fluorescent chromophores provide an innovative fluorescence system that can be tuned both in terms of the fluorescence wavelength and the quantum yield by varying the 2- and 5-substituents, respectively.^4,9^ For example, the fluorescence of the 1,3a,6a-triazapentalenes shifted to longer wavelengths due to the inductive effect of the 2-substituents. In fact, the fluorescence maxima of the 2-phenyl-1,3a,6a-triazapentalene derivatives exhibited a noteworthy correlation with the Hammett *σ*_p_ value of the substituent on the benzene ring, as shown in Fig. 1. In contrast, the introduction of an electron donating substituent at the *C*5 position had little effect on the fluorescence wavelength, although the enhancement of the push–pull effect on the 10 π-electron system was expected. Meanwhile, the fluorescence quantum yields (*Φ*_F_) were dramatically changed. In fact, the introduction of a methoxy group at the *C*5 position of 2-(4-cyanophenyl)-1,3a,6a-triazapentalene caused a substantial increase in *Φ*_F_ (from 0.15 to 0.57) without having any effect on the fluorescence wavelength .

Recently, emission- and/or quantum yield-tunable fluorophores have received a great deal of attention as the core skeleton of fluorescent probes.^11^ The 1,3a,6a-triazapentalene system also provides a novel fluorescent molecule that enables the same fluorescent chromophore to exhibit various fluorescence colors and quantum yields. However, the detailed mechanisms of the above interesting fluorescence properties have not been elucidated.

**Fig. 2:**
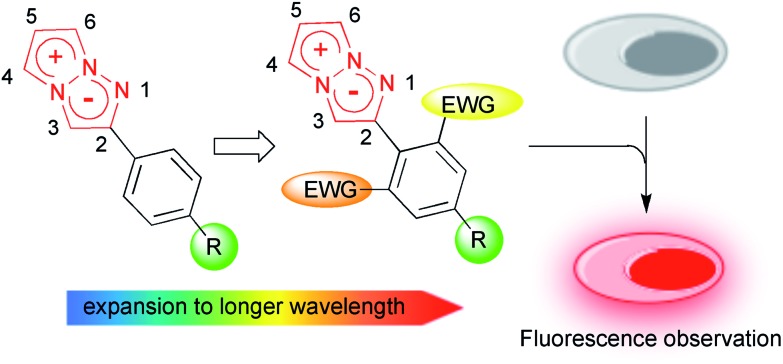
Design of yellow and orange fluorescent 1,3a,6a-triazapentalene and its application to cell staining.

To actually develop the 1,3a,6a-triazapentalenes as fluorescent labelling reagents, several goals had to be met: (i) to expand the fluorescence wavelength of the triazapentalenes to the red color region, (ii) to confirm that the fluorescence of triazapentalene from the inside of cells is observable, (iii) to introduce binding sites, such as a succinimide ester and a maleimide moiety, as labelling reagents, and (iv) to obtain the theoretical explanation of the fluorescence properties of 1,3a,6a-triazapentalene. The fluorescent labels exhibiting longer emission wavelengths, such as those emitting yellow, orange, and red light, might be more suitable for the living cells and tissues due to the reduction of the light irradiation damage and the potential access to deeper tissue. However, the existing fluorescent organic molecules emitting red light have several common problems, including a large molecular size and a small Stokes shift.^11,12^ On the other hand, 1,3a,6a-triazapentalene is a compact fluorescent chromophore exhibiting a large Stokes shift, and its fluorescence wavelength can be tuned based on the inductive effect of *C*2-substituents. Although the fluorescence wavelengths of the 1,3a,6a-triazapentalene derivatives previously reported in a preliminary communication are below the 556 nm (lime green) fluorescence wavelength of 2-(4-nitrophenyl)-1,3a,6a-triazapentalene, additional introductions of electron-withdrawing groups on the benzene ring are expected to induce additional and longer wavelength shifts. Thus, we became intrigued by the synthesis of 1,3a,6a-triazapentalenes possessing additional electron-withdrawing groups in order to investigate the possibility of 1,3a,6a-triazapentalenes emitting yellow, orange, and red light. Herein, we describe the synthesis of 2-phenyl-1,3a,6a-triazapentalene derivatives possessing both electron-withdrawing groups and binding sites on the benzene ring, the observation of their fluorescence inside cells, and the computational efforts made to provide a theoretical explanation of the fluorescence properties of the 1,3a,6a-triazapentalenes (Fig. 2).

## Results and discussion on the synthesis and the fluorescence properties

A cyano group was chosen as the electron-withdrawing group due to its small size and excellent stability under UV irradiation. Thus, a suitable position for the introduction of the cyano group to the benzene ring was first investigated. Treatments of **2** with the phenyl acetylene derivatives possessing a cyano group at the *para* **3b**, *meta* **3c**, and *ortho* position **3d** in the presence of the CuI·ligand complex and triethylamine gave the desired triazapentalenes **1b**, **1c**, and **1d** with yields of 77%, 87%, and 93%, respectively (Table 1).

**Table 1:** The orientational effects of the cyano group on the benzene ring in deaerated dichloromethane

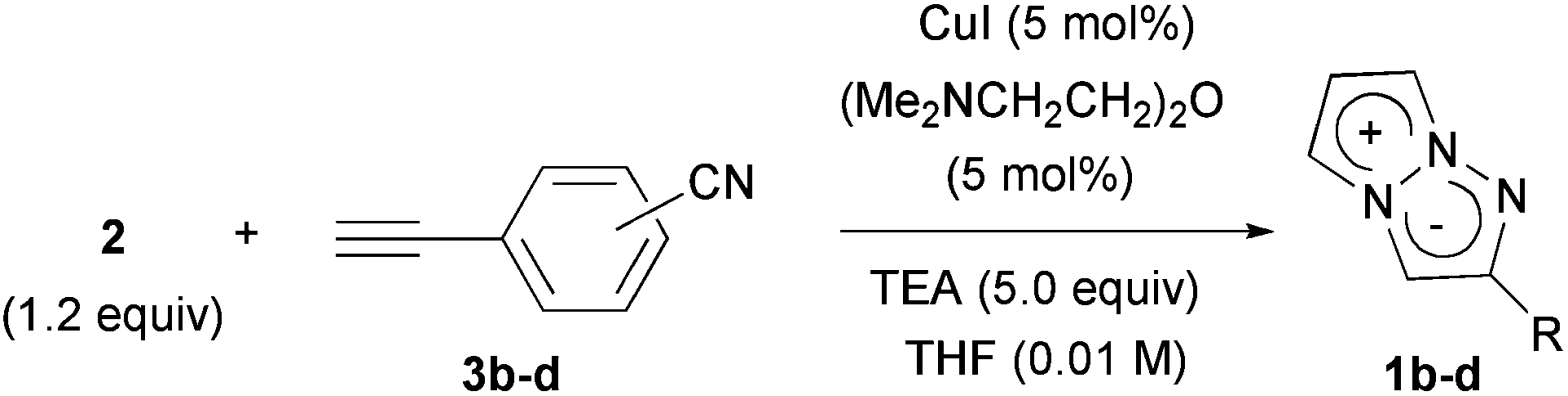					
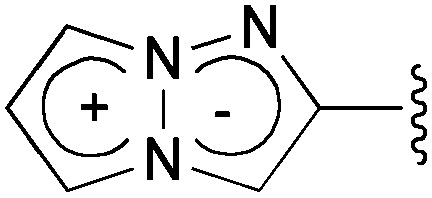	Yield (%)	*λ* max abs (nm)	*λ* max em (nm)	*Φ*_F_	Color
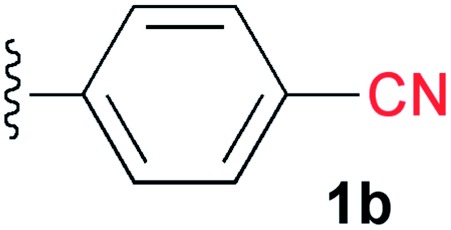	77	381	509	0.15	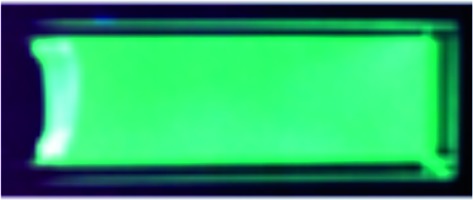
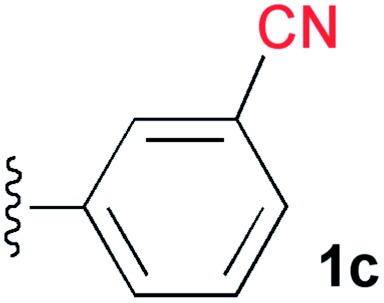	87	327	493	0.24	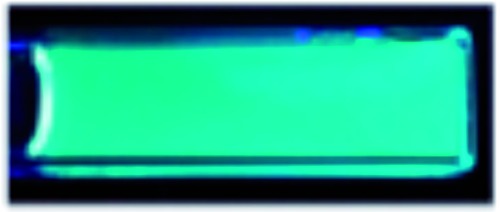
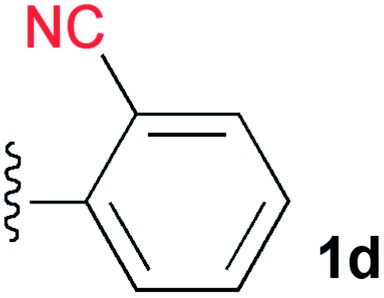	93	376	515	0.24	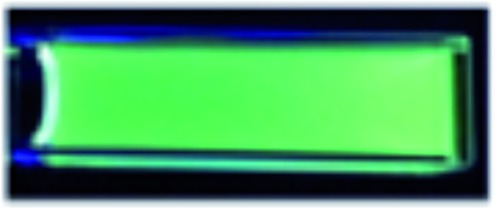

**Scheme 2:**
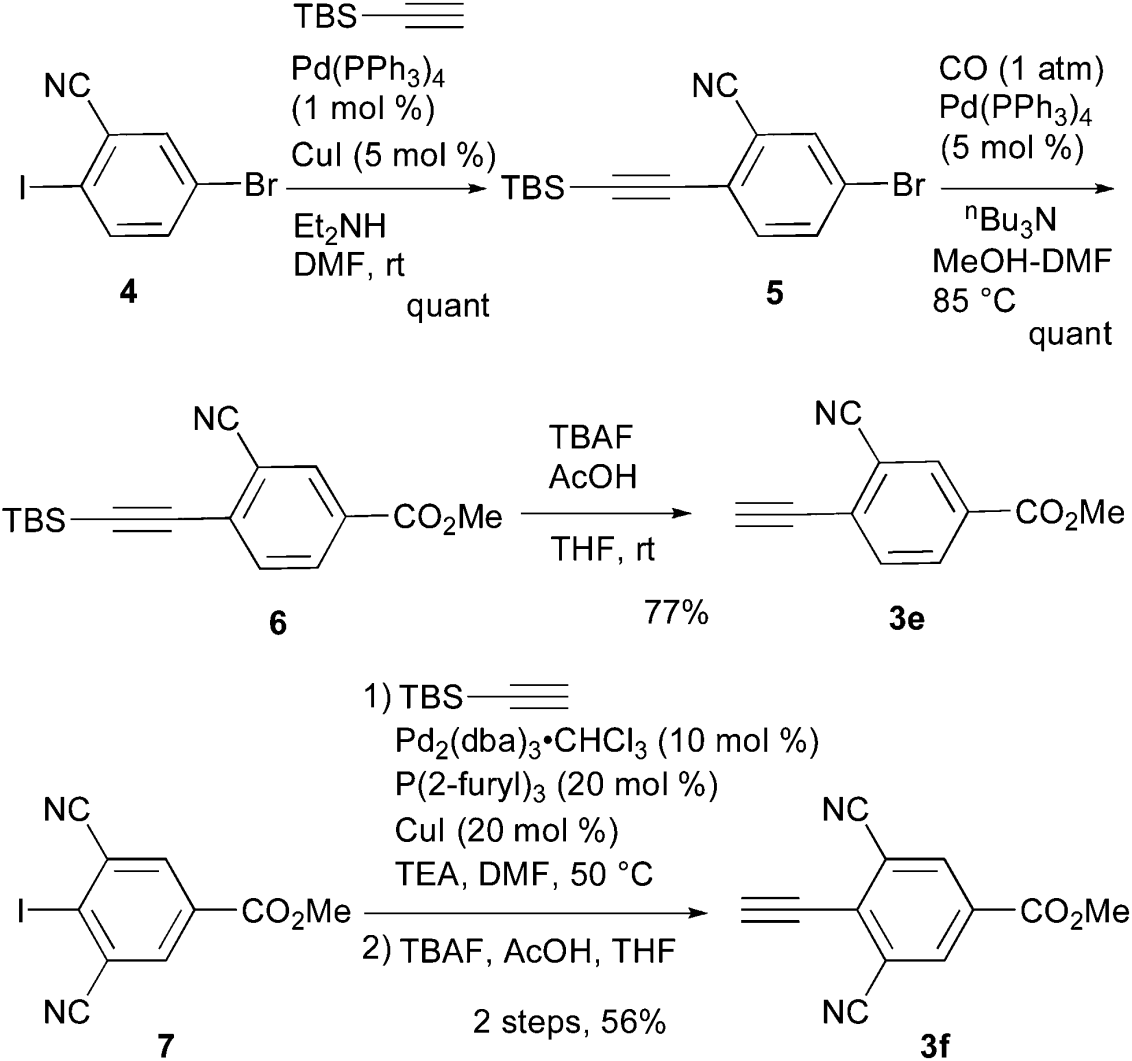
Synthesis of alkyne fragment **3e** and **3f**.

In comparison with the *para*-substituent **1b**, the introduction of a cyano group at the *meta* position (**1c**) induced the undesired shorter wavelength shift, although the *Φ*_F_ value was increased to 0.24 (Table 1).^13^ In contrast, the *ortho*-cyano analog **1d** exhibited a slightly longer-wavelength shift, and the *Φ*_F_ value was also increased (Table 1).^13^ Therefore, we found that the *ortho* position is more suitable for the introduction of the cyano group as an additional electron-withdrawing group for the expansion of the fluorescence wavelength to the yellow and red color regions. Thus, we first tried to synthesize methyl 3-cyano-4-ethynylbenzoate **3e** as an alkyne fragment. Commercially available 5-bromo-2-iodobenzonitrile **4** was converted into ethynylbenzonitrile **5** by the Sonogashira coupling reaction with *tert*-butyldimethylsilylacetylene.^14^ The treatment of **5** with 5 mol% of Pd(PPh_3_)_4_ in methanol under a CO atmosphere produced the methyl ester **6** in quantitative yield. Finally, the removal of the TBS group gave the desired alkyne fragment **3e** (Scheme 2). Next, we tried to synthesize the dicyano analog, which was expected to induce a further wavelength shift. 3,5-Dicyano-4-iodo-benzoate **7** as a starting material was obtained from the commercially available *p*-toluidine in 5 steps according to the procedure of Professor Gübel.^15^ The Sonogashira coupling reaction of **7** with various acetylenes was initially difficult, and yielded mainly the deiodinated reductive product.^16^ After various investigations, we found that the reaction with TBS–acetylene under the conditions of 10 mol% of Pd_2_(dba)_3_·CHCl_3_, 20 mol% of trifurylphosphine, 20 mol% of copper(i) iodide, and triethylamine in DMF at 50 °C produced the desired coupling product. Finally, the subsequent treatment with TBAF and acetic acid gave the alkyne fragment **3f**.

Next, the cascade reaction leading to the production of the 1,3a,6a-triazapentalenes was applied to the prepared alkynes **3e** and **3f**. The treatment of **3e** with 1.2 equiv. of **2** in the presence of 5 mol% of the CuI·ligand complex and triethylamine produced the desired 1,3a,6a-triazapentalene **1e** with a yield of 71%. The similar click reaction of **3f** also proceeded smoothly to give **1f** with a yield of 72%. Furthermore, the comparative analog **1g**, which did not possess cyano groups, was also synthesized with a yield of 73% from methyl 4-ethynylbenzoate (**3g**). Having prepared the desired 1,3a,6a-triazapentalenes **1e**, **1f**, and **1g**, their fluorescence properties were examined (Table 2). Since these three compounds were only slightly soluble in water due to the lipophilicity of the benzene ring, their fluorescence spectra were measured in deaerated dichloromethane. The standard analog **1g** exhibited a high fluorescence quantum yield (*Φ*_F_ = 0.44)^13^ and green emission (*λ*maxem = 510 mm) as predicted from the Hammett *σ*_p_ value of the methyl ester on the benzene ring. As we expected, the mono-cyano analog **1e** showed a noteworthy longer-wavelength shift of the fluorescence maximum from 510 nm of **1g** to 572 nm, and **1e** emitted yellow light. Although the fluorescence quantum yield (*Φ*_F_) of **1e** was slightly decreased to 0.34,^17^ this value was still within the range required for an effective fluorescent labelling reagent. Furthermore, the fluorescence maximum of the di-cyano analog **1f** shifted to a still longer-wavelength region (632 nm), and **1f** exhibited red fluorescence. Therefore, the introductions of the cyano groups were found to induce an approximately 60 nm longer shift of the fluorescence maximum in each case, and the development of yellow and red fluorescent 1,3a,6a-triazapentalenes was accomplished.

**Table 2:** Yields and fluorescence properties of **1g**, **1e**, and **1f**

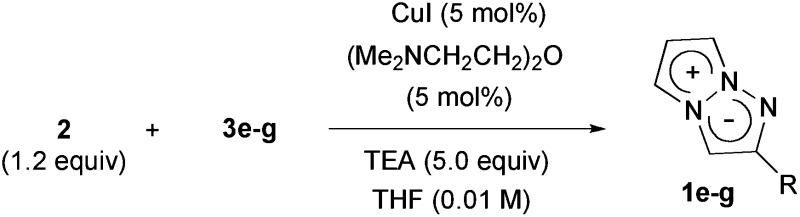					
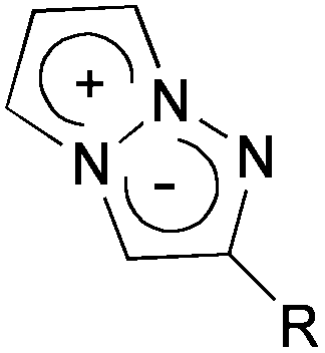	**1g**		**1e**		**1f**
	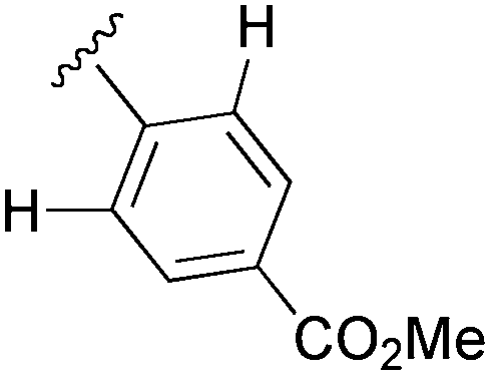		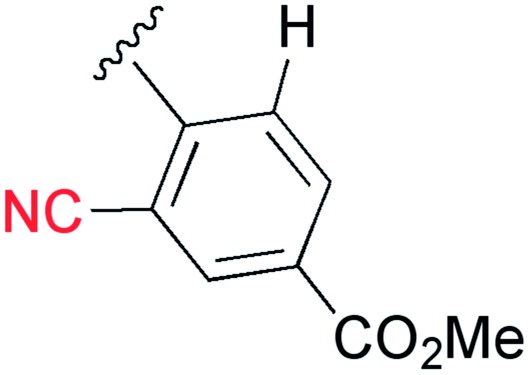		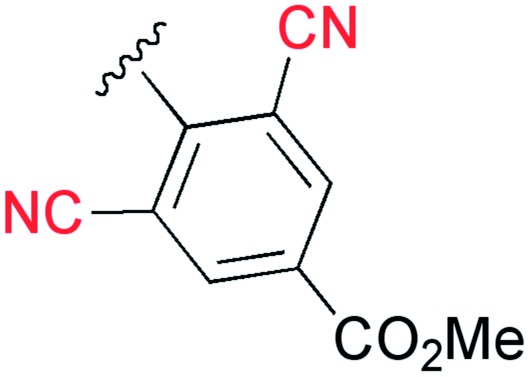
Yield	73%		71%		72%
	Solution^*a*^	Solid	Solution^*a*^	Solid	Solution^*a*^
*λ* max abs (nm)	376	N/A	420	N/A	466
*λ* max em (nm)	510	496	572	549	632
*Φ*_F_	0.44	0.06	0.34	0.06	0.096
Color	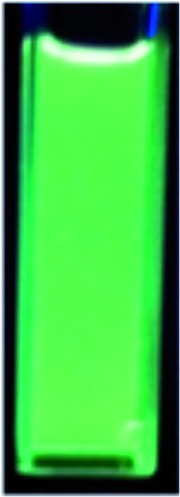	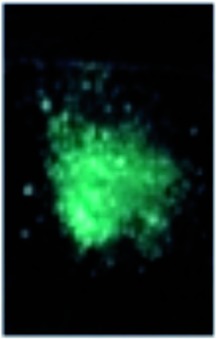	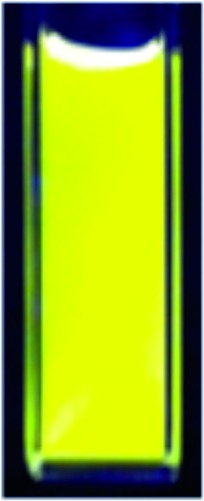	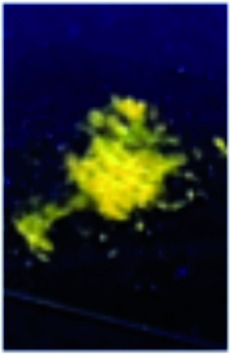	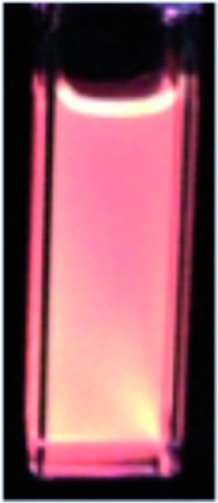

^*a*^In deaerated dichloromethane.

It was especially noteworthy that these long-wavelength fluorescent molecules exhibited large Stokes shifts, such as the 152 nm shift of **1e** and the 166 nm shift of **1f**, despite there having been few prior examples of the long-wavelength (>550 nm) organic fluorophores exhibiting such large (mega) Stokes shifts,^18^ since such shifts were useful for suppressing the action of background fluorescence in the various fluorescence analyses. In addition, the molecular sizes of **1e** and **1f** were considerably smaller in comparison with the conventional yellow and red fluorescent molecules. Therefore, the 1,3a,6a-triazapentalenes might be practical fluorescent chromophores for use as molecular probes to cover the entire region of visible wavelengths, although the further shift toward longer wavelengths is still needed. Furthermore, **1g** and **1e** exhibited fluorescence emission in the solid state with a fluorescence maximum similar to that observed in the solution of dichloromethane, whereas the fluorescence of **1f** in the solid state was not detected.

On the other hand, the extinction coefficient (*ε*) of **1g** at 376 nm was 1230 dm^3^ mol^–1^ cm^–1^, and this value still needed to be increased for a more bright fluorescent reagent. Although the *ε* value at 287 nm was 13 800 dm^3^ mol^–1^ cm^–1^, at a practical level, this region (ultraviolet) is not a suitable excitation light for imaging probes. Similarly, the extinction coefficients (*ε*) of **1e** and **1f** in a visible light region were also not high at 630 dm^3^ mol^–1^ cm^–1^ (420 nm) and 1580 dm^3^ mol^–1^ cm^–1^ (466 nm), respectively. Therefore, the improvement of the extinction coefficient (*ε*) was the next challenge for the development of more useful bright fluorescent labels. So far, we have already found that the introduction of a substituent at the *C*4 position dramatically increases the *ε* value. For example, 4-phenyl analogs of **1g** showed a substantial increase in the *ε* value from 1230 dm^3^ mol^–1^ cm^–1^ (376 nm) for **1g** to 22 600 dm^3^ mol^–1^ cm^–1^ (345 nm) with comparable *Φ*_F_ values. The 4-phenyl analog of **1e** also exhibited a practical *ε* value of 4560 dm^3^ mol^–1^ cm^–1^ (432 nm) and 38 000 dm^3^ mol^–1^ cm^–1^ (336 nm), although the *Φ*_F_ value was decreased to 0.07.^19^ Further investigation of 4-substituents for the design of practical fluorescent labelling reagents is currently underway in our laboratory.

**Fig. 3:**
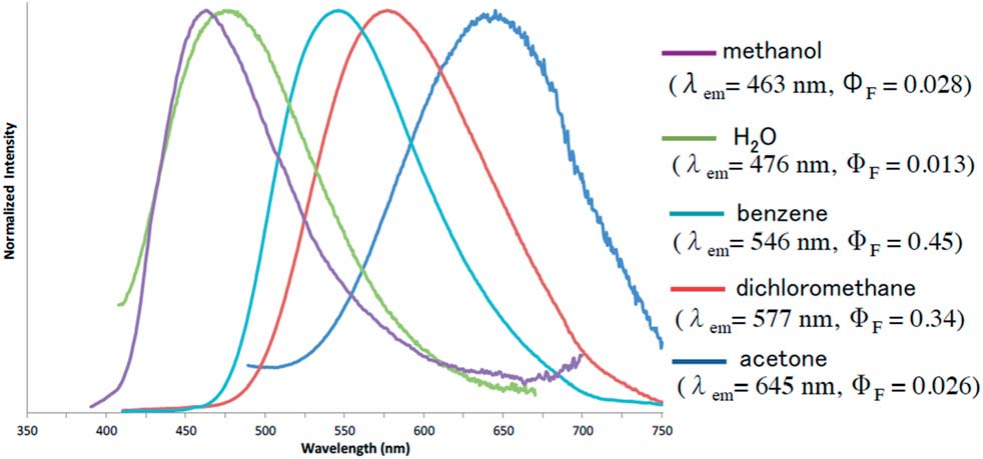
Emission behaviors and the fluorescence spectra of **1e** in several solvents.

Furthermore, the fluorescence solvatochromism of **1e** was examined. The fluorescence spectra of **1e** in several solvents are shown in Fig. 3. Basically, the fluorescence of **1e** shifted to the longer wavelength with the Stokes shift being increased by an increase in the solvent polarity from benzene (546 nm) to acetone (645 nm). On the other hand, its fluorescence in methanol shifted inversely to the shorter wavelength (*λ*maxem = 463 nm). Furthermore, since **1e** was only slightly soluble in water, its fluorescence in water was also measured. The fluorescence shifted to 476 nm similarly to the fluorescence shift observed in methanol. The fluorescence quantum yield (*Φ*_F_) in water was substantially decreased to a value of 0.013. Therefore, the 1,3a,6a-triazapentalenes are expected to change their fluorescence wavelength and intensity according to the hydrophobic environment in the cells.

**Fig. 4:**
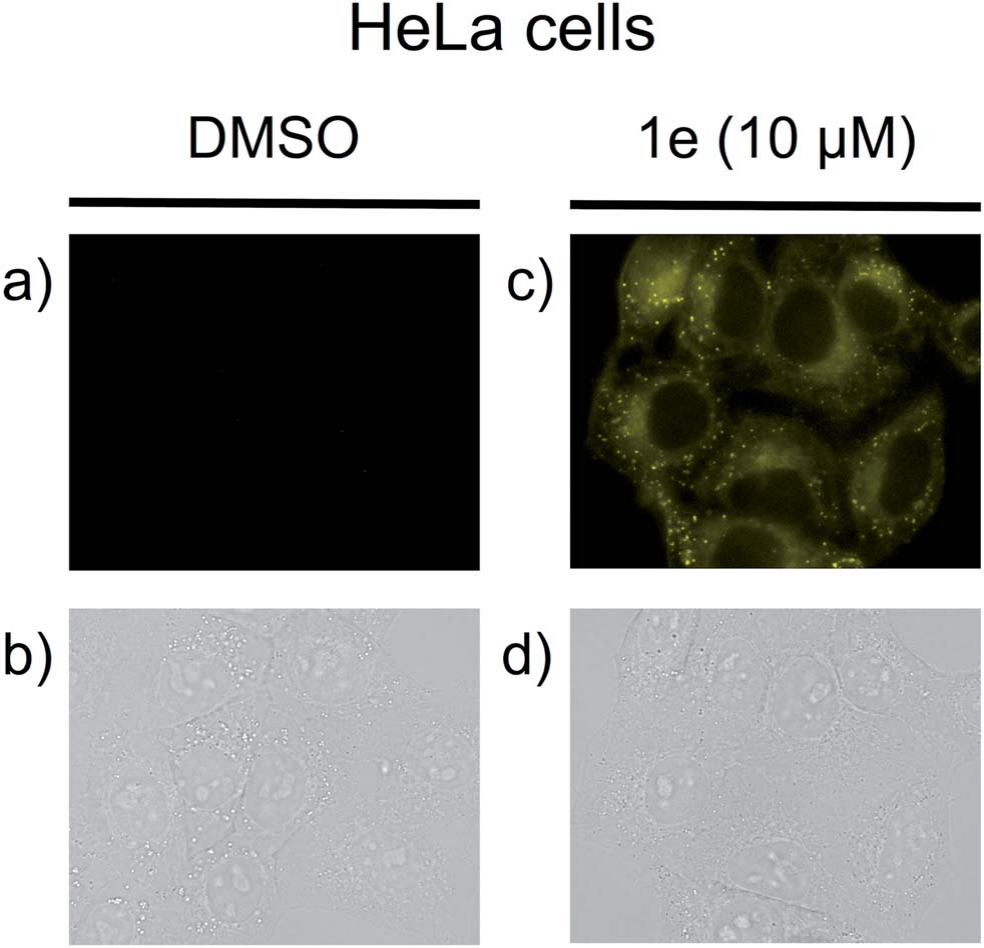
The observation of **1e** in HeLa cells. Living cells were cultured in 0.02% DMSO as a control (a and b) or with 10 μM **1e** in 0.02% DMSO (c and d). The uptake of **1e** was monitored by using a fluorescence microscope (BZ-9000; Keyence) with a bright-field image (b and d) or with a fluorescence image (a and c) with a BZ set (FF01-452/45 nm exciter, FF01-607/70 nm emitter, FF511 nn-Di01 dichroic mirror).

Next, we investigated the applicability of the long-wavelength fluorescent 1,3a,6a-triazapentalenes as fluorescent probes in a biological system. Since the di-cyano analog **1f** was not very stable under UV irradiation and its *Φ*_F_ was lower (*Φ*_F_ = 0.096) than that of **1e** (*Φ*_F_ = 0.34),^17^ the mono-cyano analog **1e** was adopted for this purpose. Thus, HeLa cells were treated with a solution of **1e** (10 μM in 0.02% DMSO)^20^ and monitored in the 572–642 nm wavelength region. As shown in Fig. 4, the fluorescent staining of HeLa cells was successfully observed without washing the cellular medium. The living HeLa cells were clearly visualized as observed using a fluorescence microscope, whereas the interiors of the control cells, which were treated with DMSO, were not stained. Since the active uptake of the fluorescent **1e** by living cells and the fluorescence solvatochromism of **1e** enhance the fluorescence contrast between the cells and the background, it was not necessary to fix the cells. Furthermore, a cytotoxic effect on the cells was not identified over the observation period, suggesting that the triazapentalene is suitable for connecting to small biofunctional molecules as a fluorescent label. This is the first experimental evidence that the 1,3a,6a-triazapentalene is applicable to the life sciences field as a fluorescent reagent. Further detailed investigations into the localization and quantitative analysis of **1e** inside cells are currently underway in our laboratory.

**Scheme 3:**
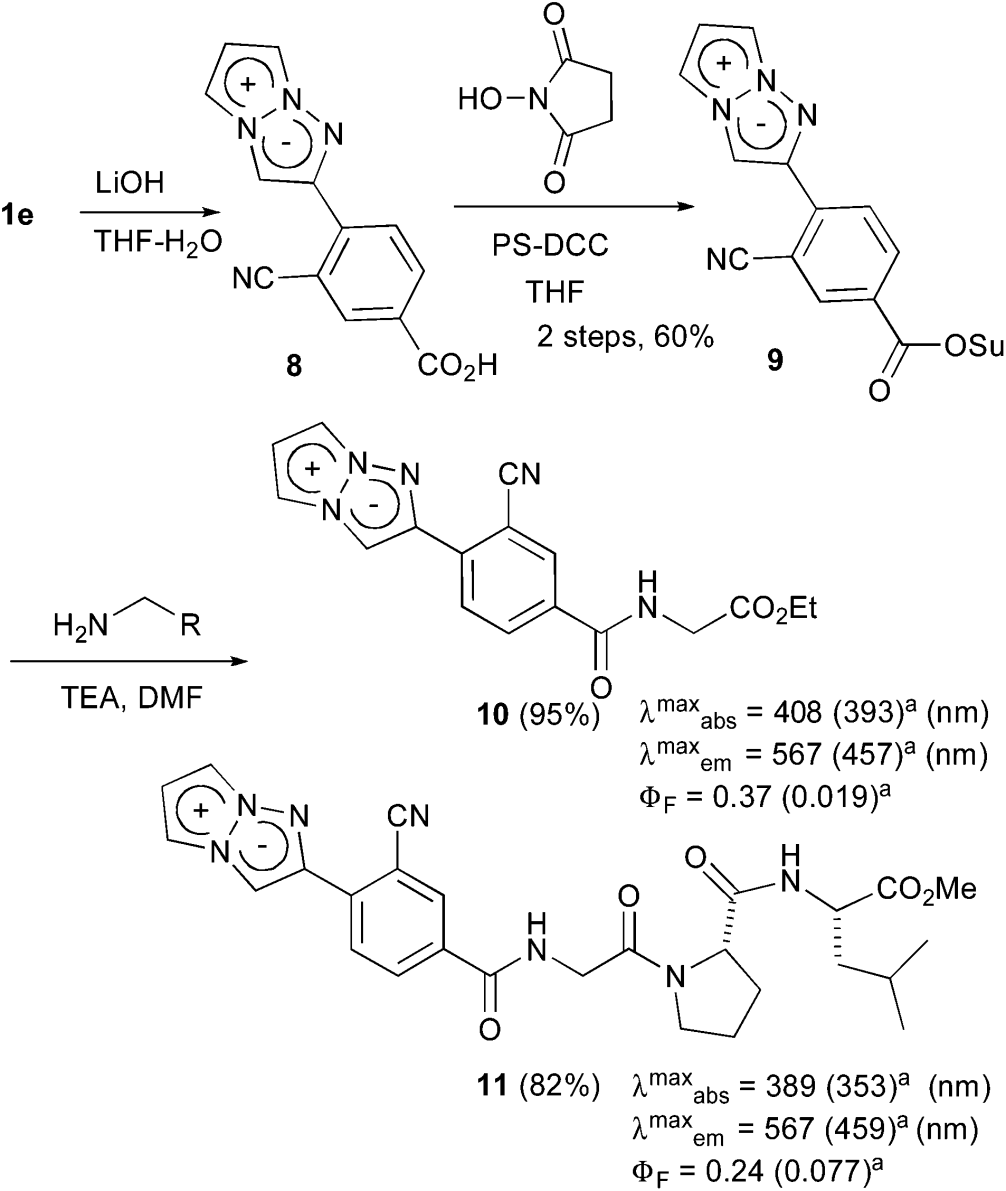
The synthesis of the fluorescent labelling reagent **11** and its application to the amino acids. ^*a*^Measured in water.

The actual fluorescence observation of **1e** inside cells encouraged us to develop **1e** as a fluorescent labelling reagent. Thus, the conversion of the methyl ester moiety into the *N*-hydroxysuccinimide ester as a binding site was attempted. The treatment of **1e** with 1.2 equiv. of lithium hydroxide afforded carboxylic acid **8**, which was directly used for the next condensation reaction. However, although the condensation reaction proceeded smoothly, the removal of the urea analogs generated from the condensing reagent was not straightforward due to the instability of the succinimide moiety of **9**. Finally, polymer-supported DCC was adopted as a useful condensing reagent to remove the urea by filtration, and the subsequent recrystallization gave the purified **9** in a 60% two-step yield. Having prepared the fluorescent labelling reagent **9**, the introduction of **9** into amino acids was examined (Scheme 3). The treatment of **9** with glycine ethyl ester in DMF produced labelled glycine **10** in 95% yield. The fluorescence-labelled **10** exhibited yellow emission (*λ*maxem = 567 nm) with a high quantum yield (*Φ*_F_ = 0.37)^17^ in deaerated dichloromethane. Furthermore, the introduction of the tri-peptide Gly-Pro-Leu was also examined, and the labelled tri-peptide **11** was obtained in 82% yield. The fluorescence observation of **11** showed a fluorescence maximum at 567 nm and an acceptable fluorescence quantum yield (*Φ*_F_ = 0.24)^17^ in deaerated dichloromethane. Therefore, the development of the 1,3a,6a-triazapentalene as a compact fluorescent labelling reagent emitting yellow-red light was achieved. Furthermore, although the labelled glycine **10** and tri-peptide **11** were dissolved well in an organic solvent,^21^ their fluorescence properties in water were also measured. Since the emission maxima of **10** and **11** shifted to shorter wavelengths with similar absorption maxima, the Stokes shifts became small in water as in the case of **1e**. The fluorescence quantum yields (*Φ*_F_) were also reduced to 0.019 (**10**) and 0.077 (**11**).^17^ These changes in the fluorescence properties according to the polarity of the environment might make the 1,3a,6a-triazapentalene useful as a fluorescent probe *in vivo* measurements.

### Theoretical investigation of the optical properties of the 1,3a,6a-triazapentalenes

In our preliminary communication, we first reported that the 1,3a,6a-triazapentalene skeleton without an additional fused ring system is a compact and highly fluorescent chromophore. However, the detailed mechanisms of the fluorescence have not yet been elucidated. In this work, quantum chemical calculations were performed to investigate the optical properties of the 1,3a,6a-triazapentalenes. Most of the theoretical calculations for the optical properties of dye molecules utilize the time-dependent density functional theory (TD-DFT), but in this work the high-level wavefunction-based approach using the complete active space second-order perturbation theory (CASPT2) method are also employed to provide a more reliable description of the excitation energies. The following synthetic 1,3a,6a-triazapentalenes were examined as the model substrates in this investigation: unsubstituted 1,3a,6a-triazapentalene **1a** as a basic structure, 2-(4-cyano)phenyl derivative **1b** as a standard analog described in the previous communication, and synthetic **1g**, **1e**, and **1f** as described in this article.

### Computational details

The equilibrium geometry in the electronic ground state (S_0_) is determined by the density functional theory (DFT) calculations using the B3LYP functionals, while the geometry optimization in the lowest ππ* excited state S_1_(ππ*) is performed by the time-dependent DFT (TD-DFT) calculations employing the coulomb attenuated B3LYP (CAM-B3LYP) functionals.^22^ The *C*_s_ symmetry constraint is imposed for **1a**, **1b**, **1g**, and **1e**, while no constraint is applied for **1f** because the twisted structure is more stable due to the steric hindrance. The choice to employ the CAM-B3LYP functionals is due to the significant charge-transfer character involved in excitation to the S_1_ state. The 6-31 + G(d,p) basis set is used in the DFT calculations and the equilibrium geometries are determined both in the gas phase and in dichloromethane. The solvent effects are taken into account by the polarizable continuum solvation model (PCM),^23^ where the radii are taken from the universal force field.^24^ After the geometry optimization, the vertical excitation and fluorescence energies are calculated at the S_0_ and S_1_ equilibrium structures (denoted as (S_0_)_min_ and (S_1_)_min_), respectively, by the TD-DFT(CAM-B3LYP) method. In PCM calculations, the linear-response method with a non-equilibrium solvation is employed to obtain the vertical excitation energies at (S_0_)_min_, while the equilibrium solvation is adapted for the calculation of the excitation energies during the S_1_ geometry optimization.

The excitation energy is also refined at the DFT-optimized geometries by the CASPT2 (ref. 25) method in order to obtain more reliable excitation energies. A level shift with a value of 0.3 is applied for the CASPT2 calculations.^26^ The notation of CASPT2 (*m*,*n*) is occasionally used, in which case the active space for a reference state-averaged complete active space self-consistent field (SA-CASSCF) wavefunction is composed of *m* electrons and *n* orbitals (SA-CASSCF (*m*,*n*)). The augmented correlation-consistent polarized double-zeta basis set (denoted as aug-cc-pVDZ) is employed in the CASPT2 calculations. For obtaining the oscillator strengths, the vertical excitation energies calculated by CASPT2 and the transition dipole moments calculated by SA-CASSCF are used.

For **1a**, the active space for the reference SA-CASSCF wavefunction is comprised of six π orbitals (four π orbitals are doubly-occupied and two are unoccupied in the closed-shell configuration), and it is therefore denoted as SA-CASSCF(8,6). **1a** possesses ten π orbitals and the lowest and highest π orbitals are excluded from the active space. This is justified by the larger active space calculation, which includes all π orbitals (which corresponds to SA-CASSCF(10,8), and the active orbitals at (S_0_)_min_ are shown in the ESI as Fig. S1†), where only a difference of ∼0.01 eV is observed in the S_1_ vertical excitation energies. The active space for the other chromophores is composed of twelve electrons distributed in ten π orbitals (SA-CASSCF(12,10)), and the active orbitals of **1b** at (S_0_)_min_ are shown in Fig. S2.† As seen in the figure, the active space of the SA-CASSCF(12,10) wavefunction includes orbitals that correspond to the active orbitals of SA-CASSCF(8,6) in **1a**. For all chromophores, the S_0_ and S_1_ states are averaged with equal weights in the SA-CASSCF calculations, except where otherwise noted.

The DFT and TD-DFT calculations are performed using the Gaussian09 program package^27^ while the CASPT2 calculations are carried out using the MOLPRO2010.1 program package.^28^

## Results and discussion on the optical properties

We begin by investigating the character of the excited states of **1a** and **1b** at (S_0_)_min_ in the gas phase, followed by the results and discussion on the optical properties of the other chromophores in the gas phase and in dichloromethane.

### 1. Simple 1,3a,6a-triazapentalene (**1a**)

**Fig. 5:**
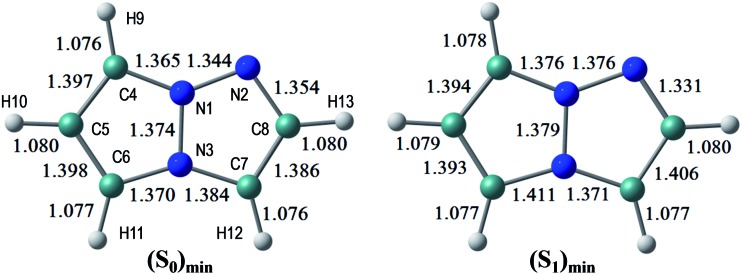
The equilibrium structures of **1a** in the S_0_ and S_1_ states in the gas phase. The bond lengths are given in units of Å.

The S_0_ and S_1_ equilibrium structures of **1a** in the gas phase are shown in Fig. 5, along with the bond lengths and the atomic numbering (note that this numbering is different from the previous sections and is only used in the theoretical section). The significant changes in geometry upon photo-excitation involve the bond elongation of N3–C6 (1.370 → 1.411 Å) and N1–N2 (1.344 → 1.376 Å).

The vertical excitation energies to the low-energy-lying ππ* states are shown in Table 3, where in the CASPT2 calculation the S_0_ and lowest three ππ* states are averaged with equal weights in the reference SA-CASSCF(8,6) wavefunction. It is noted that, although a couple of nπ* states are found between these ππ* states in the TD-DFT calculations, it is confirmed that the lowest-energy singlet excited-state is characterized by the ππ* excitation, and therefore only the ππ* states are examined in this investigation. The lowest ππ* excited-state, S_1_(ππ*), is viewed as the HOMO–LUMO transition (see the natural orbitals in Fig. S1 (ESI)†) and the CASPT2 excitation energy of 4.33 eV (286 nm) is in good agreement with the experimental value of 4.31 eV (288 nm), even though the experimental measurements are performed in dichloromethane. The second ππ* excited state is characterized by the HOMO → LUMO+1 transition, and it lies close to the first ππ* state in the CASPT2 calculation. The natural charges of the S_0_ and S_1_ states at (S_0_)_min_ and their differences are shown in Fig. S3.†

**Table 3:** The vertical excitation energies (Δ*E* in eV and nm) and oscillator strengths (*f* in a.u.) of **1a** for the low-lying ππ* states at (S_0_)_min_ ^*a*^

	TD-DFT (CAM-B3LYP)				CASPT2			
State	Δ*E* (eV)	Δ*E* (nm)	*f*	Transition	Δ*E* (eV)	Δ*E* (nm)	*f*	Transition
1	4.79	259	0.262	5π → 1π*	4.33	286	0.213	5π → 1π*
2	5.33	232	0.052	5π → 2π*	4.43	280	0.250	5π → 2π*
3	5.49	225	0.010	5π → 3π*	5.53	224	0.384	4π → 1π*

^*a*^The main orbital transition is also shown.

### 2. 2-(4-Cyano)phenyl-1,3a,6a-triazapentalene (**1b**)

**Fig. 6:**
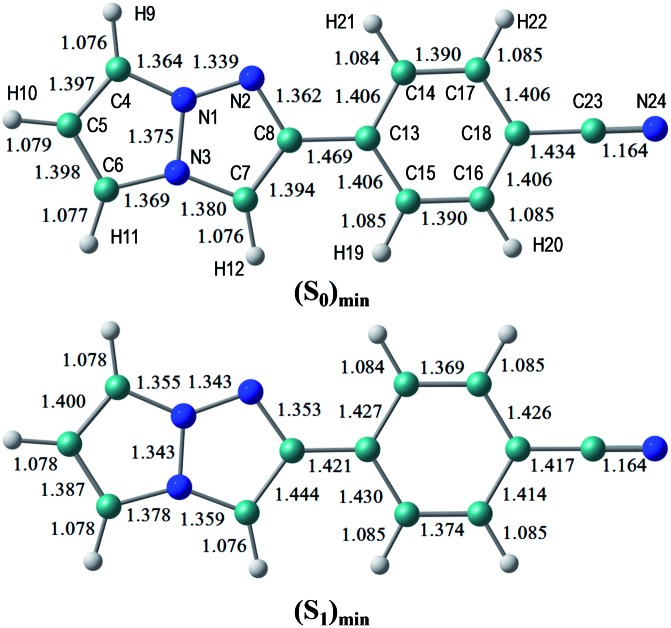
Equilibrium structures of **1b** in the S_0_ and S_1_ states in the gas phase. The bond lengths are given in units of Å.

The S_0_ and S_1_ equilibrium structures of **1b** in the gas phase are shown in Fig. 6, along with the bond lengths and atomic numbering. The transition to the S_1_ state involves the bond elongation of C7–C8 (1.394 → 1.444 Å) and shortening of the central C8–C13 bond (1.469 → 1.421 Å).

**Fig. 7:**
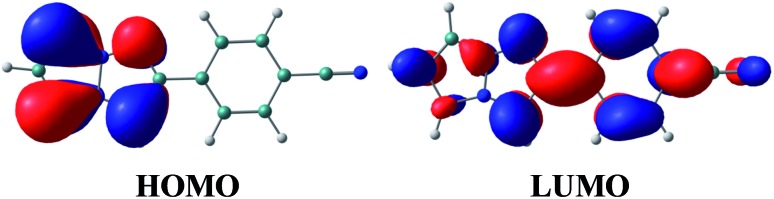
Natural orbitals of **1b** involved in the excitation to the S_1_ state.

The vertical excitation energies to the low-energy-lying ππ* states are shown in Table 4. In the CASPT2 calculation, the S_0_ and the lowest three ππ* states are averaged with equal weights in the reference SA-CASSCF(12,10) wavefunction. The vertical excitation energies to the S_1_(ππ*) state are 3.85 (322 nm) and 3.25 eV (381 nm) for the TD-DFT and CASPT2 calculations, respectively, and the CASPT2 excitation energy is in remarkably good agreement with the experimental value of 3.25 eV (381 nm) (note again that the experimental measurements are performed in dichloromethane). Excitation to the S_1_ state is characterized by the HOMO → LUMO transition (see Fig. 7 and also Fig. S2†), and as expected from the shape of the two relevant orbitals, the S_1_ transition involves charge transfer from the 1,3a,6a-triazapentalene skeleton to the substituted phenyl ring. This is clearly seen from the large dipole moment in the S_1_ state (19.47 debye) compared to that of the S_0_ state (7.11 debye) at (S_0_)_min_ (see Table S1†). The charge-transfer character of S_1_ is also clear from the natural charges, where the sums of the natural charges in the 1,3a,6a-triazapentalene skeleton (atoms from N1 to H12) are 0.022 and 0.542 in the S_0_ and S_1_ states, respectively (see also Table S1†). Since the S_1_ state exhibits a charge-transfer character, it may be possible to observe the twisted intramolecular charge transfer (TICT) state involving the rotation of the phenyl ring around the central C8–C13 bond. In order to check this, we performed frequency analysis at (S_1_)_min_ and confirmed that the planar geometry is the minimum energy structure in the S_1_ state.

**Table 4:** The vertical excitation energies (Δ*E* in eV and nm) and oscillator strengths (*f* in a.u.) of **1b** for the low-energy-lying ππ* states at (S_0_)_min_ ^*a*^

	TD-DFT (CAM-B3LYP)				CASPT2			
State	Δ*E* (eV)	Δ*E* (nm)	*f*	Transition	Δ*E* (eV)	Δ*E* (nm)	*f*	Transition
1	3.85	322	0.083	9π → 1π*	3.25	381	0.047	9π → 1π*
2	4.67	266	0.043	9π → 2π*, 8π → 1π*	3.89	319	0.755	9π → 2π*, 8π → 1π*
3	4.80	258	1.111	9π → 2π*, 8π → 1π*	4.17	298	0.012	9π → 2π*, 8π → 1π*

^*a*^The main orbital transition is also shown.

As seen in Table 4, the second and third ππ* states can be described as a mixing of two configurations, HOMO → LUMO+1 (9π → 2π*) and HOMO–1 → LUMO (8π → 1π*). It is noted that the HOMO → LUMO+1 (9π → 2π*) transition corresponds to the S_0_–S_1_ excitation of **1a**, while the S_0_–S_1_ transition of **1b** corresponds to the excitation to the second ππ* state of **1a** (see the natural orbitals given in Fig. S1 and S2†). Therefore, the electronic character of the S_1_ state is different between **1a** and **1b**.

### 3. Green fluorescence (**1g**), yellow fluorescence (**1e**), and red fluorescence (**1f**) derivatives and comparison with the experimental results

**Fig. 8:**
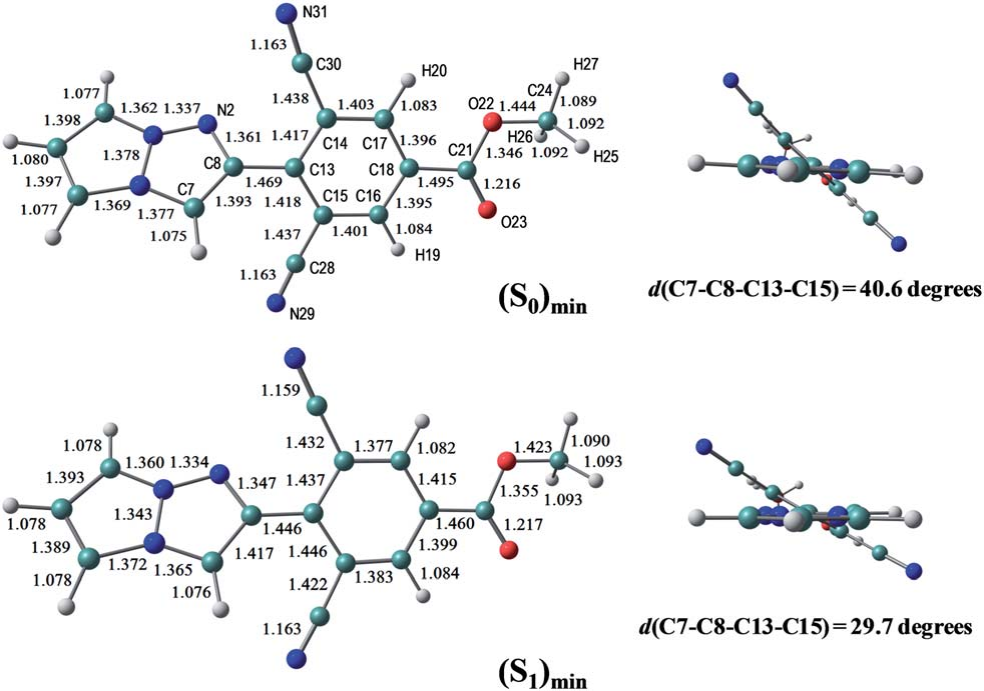
The equilibrium structures of **1f** in the S_0_ and S_1_ states in the gas phase. The bond lengths are given in units of Å.

The optimized structures of **1f** in the S_0_ and S_1_ states are shown in Fig. 8, where the dihedral angle of *d*(C7–C8–C13–C15) representing twisting of the phenyl ring around the central C8–C13 atoms is 40.6 degrees at (S_0_)_min_, and slightly decreases to 29.7 degrees at (S_1_)_min_. The other chromophores (**1g** and **1e)** maintain the planar geometry, and the Cartesian coordinates of the optimized structures are given in the ESI.†

Excitation to the S_1_ state involves the HOMO → LUMO transition, and all chromophores (**1g**, **1e**, and **1f**) exhibit the same charge-transfer character. The vertical excitation and fluorescence energies are summarized in Table 5 and Table 6, respectively. We note here that in this table a slight discrepancy is found in the S_1_(ππ*) vertical excitation energies of CASPT2 for **1a** and **1b** with respect to the values shown in Table 3 and 4, since in Table 5 only the S_0_ and S_1_ states are averaged with equal weights in the reference SA-CASSCF wavefunction.

**Table 5:** The vertical excitation energies (Δ*E* in eV and nm) and oscillator strengths (*f* in a.u.) for the S_1_ state calculated by TD-DFT and CASPT2 at (S_0_)_min_

	TD-DFT (in gas)			TD-DFT (in dichloromethane)			CASPT2 (in gas)			Exp.
	Δ*E* (eV)	Δ*E* (nm)	*f*	Δ*E* (eV)	Δ*E* (nm)	*f*	Δ*E* (eV)	Δ*E* (nm)	*f*	Δ*E* (nm)
**1a**	4.79	259	0.262	4.62	268	0.276	4.26	291 (300)^*a*^	0.351	288
**1b**	3.85	322	0.083	3.66	339	0.143	3.26	380 (397)	0.058	381
**1g**	3.91	317	0.096	3.73	332	0.159	3.32	374 (389)	0.057	376
**1e**	3.51	353	0.048	3.33	372	0.076	2.97	418 (437)	0.051	420
**1f**	3.21	387	0.033	3.11	398	0.050	2.76	450 (461)	0.040	466

^*a*^The number in parenthesis is an estimate in dichloromethane.

**Table 6:** The vertical fluorescence energies (Δ*E* in eV and nm) and oscillator strengths (*f* in a.u.) for the S_1_ state calculated by TD-DFT and CASPT2 at (S_1_)_min_

	TD-DFT (in gas)			TD-DFT (in dichloromethane)			CASPT2 (in gas)			Exp.
	Δ*E* (eV)	Δ*E* (nm)	*f*	Δ*E* (eV)	Δ*E* (nm)	*f*	Δ*E* (eV)	Δ*E* (nm)	*f*	Δ*E* (nm)
**1a**	4.62	268	0.276	4.33	287	0.446	4.04	307 (326)^*a*^	0.382	389
**1b**	3.46	358	0.137	3.18	390	0.345	2.88	430 (462)	0.092	509
**1g**	3.48	356	0.164	3.19	389	0.372	2.91	426 (459)	0.101	510
**1e**	3.14	394	0.072	2.91	425	0.191	2.63	471 (502)	0.078	572
**1f**	2.82	440	0.046	2.68	463	0.134	2.33	533 (556)	0.060	632

^*a*^The number in parenthesis is an estimate in dichloromethane.

The CASPT2 calculations are performed only in the gas phase, and therefore we estimate the excitation energies in dichloromethane using the solvatochromic shifts of TD-DFT calculations (the estimated values are shown in parenthesis).

**Fig. 9:**
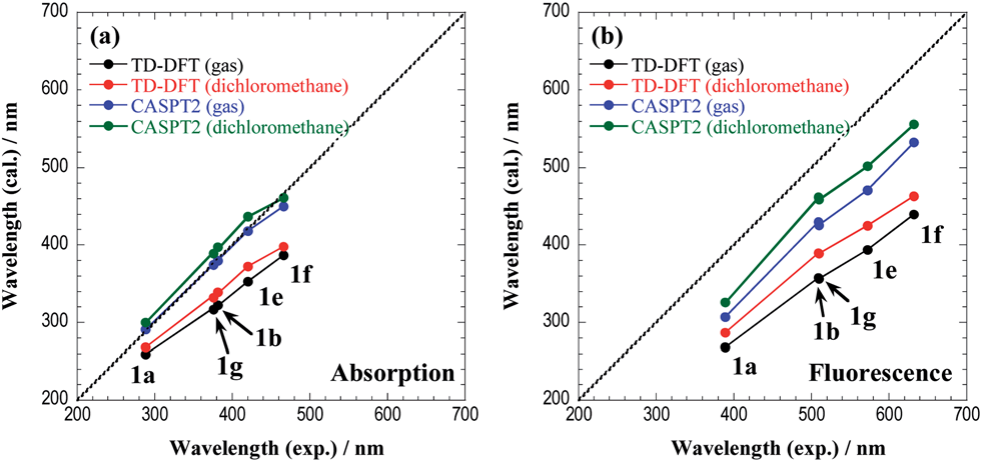
The comparison of (a) the absorption and (b) the fluorescence wavelengths between the theoretical calculations and experimental results. The central line indicates a perfect theory/experiment match.

Fig. 9 shows the comparison of absorption and fluorescence wavelengths between the theoretical calculations and experimental results. Although the calculated fluorescence wavelengths are shorter than the experimental values, the figure clearly demonstrates a good correlation between the two values. The overestimation of the fluorescence energies may be attributed to the insufficient treatment of the solvent environments, because excitation involves a significant charge-transfer character. The explicit treatment of the solvent molecules in the framework of the QM/MM approach or the state-specific approach^29,30^ would be appropriate for a more quantitative description of the fluorescence energies.

In the ESI (Tables S1 and S2†), the sums of the natural charges in the 1,3a,6a-triazapentalene skeleton and the dipole moments in the S_0_ and S_1_ states at (S_0_)_min_ and (S_1_)_min_ are given for all chromophores. It is noteworthy that there is a clear correlation between the wavelengths and the natural charges (also the dipole moments) in the S_1_ state, where a larger charge separation induces longer absorption and fluorescence wavelengths. It is also noted that the absorption and fluorescence wavelengths are longer when measured in dichloromethane than when measured in the gas phase because the charge-transfer state is more stabilized in polar solvents.

Finally, we comment that the CASPT2 method is more reliable than the TD-DFT approach, but the computational cost is much more expensive. As seen in the present work, the TD-DFT method predicts slightly higher excitation energies than those by CASPT2, but the correlation with experimental results is surprisingly good. Therefore, for chromophores of a larger size, where the computational costs of CASPT2 calculations are prohibitive, the TD-DFT method can be reliably used to predict the optical properties of 1,3a,6a-triazapentalenes.

## Conclusions

The fluorescence wavelengths of 1,3a,6a-triazapentalenes were extended to the red color region. Based on the noteworthy correlation of the fluorescence wavelength with the inductive effect of the 2-substituent, electron deficient 2-(2-cyano-4-methoxycarbonylphenyl)-1,3a,6a-triazapentalene and 2-(2,6-dicyano-4-methoxycarbonylphenyl)-1,3a,6a-triazapentalene were synthesized. They exhibited yellow and red fluorescence and a large Stokes shift respectively, and the 1,3a,6a-triazapentalene system enabled the same fluorescent chromophore to cover the entire region of visible wavelengths. The potential applications of the 1,3a,6a-triazapentalene system as fluorescent probes in the fields of the life sciences were investigated, and the 1,3a,6a-triazapentalene system was clearly proven to be useful as a fluorescent reagent for living cells. The *N*-hydroxysuccinimide ester derivative of yellow fluorescent 1,3a,6a-triazapentalene as a compact labelling reagent was confirmed to be able to readily label the amino group. Finally, quantum chemical calculations were performed to investigate the optical properties of the 1,3a,6a-triazapentalenes. These calculations revealed that excitation involves significant charge-transfer from the 1,3a,6a-triazapentalene skeleton to the 2-substitutent. The calculated absorption and fluorescence wavelengths showed a good correlation with the experimental ones, which allows us to design substituents that exhibit the desired optical properties.
