# Supplementary material for: Synthesis of yellow and red fluorescent 1,3a,6a-triazapentalenes and the theoretical investigation of their optical properties
Source: Chem Sci. 2014 Oct 23;6(2):1083–93. doi: 10.1039/c4sc02780a (PMC5811129; doi:10.1039/c4sc02780a)

Electronic Supplementary Material (ESI) for Chemical Science.
This journal is © The Royal Society of Chemistry 2014

Supporting Information

Synthesis of Yellow and Red Fluorescent 1,3a,6a-Triazapentalene and Theoretical Investigation of Optical Properties.

Kosuke Namba[†]*, Ayumi Osawa[§], Akira Nakayama[#]*, Akane Mera[§], Fumi Tano[§], Yoshiro Chuman[‡], Eri Sakuda[‡], Tetsuya Taketsugu[‡], Kazuyasu Sakaguchi[‡], Noboru Kitamura[‡] and Keiji Tanino[‡]*

[†]*Depertment of Pharmaceutical Science, The University of Tokushima, 1-78 Shomachi. Tokushima 770-8505, Japan*

[‡]*Department of Chemistry, Faculty of Science, Hokkaido University, Kita-ku, Sapporo 060-0810, Japan*

[§]*Graduate School of Chemical Sciences and Engineering, Hokkaido University, Sapporo 060-0810, Japan*

[#]*Catalysis Research Center, Hokkaido University, Sapporo 001-0021, Japan*

E-mail: namba@mail.sci.hokudai.ac.jp; nakayama@cat.hokudai.ac.jp; and ktanino@sci.hokudai.ac.jp

• Experimental Detail for the Synthesis of 1,3a,6a-Triazapentalenes S2–S10

• Absorption and Fluorescence spectra of triazapentalenes S11–S15

• Molecular orbitals, natural charges, dipole moments, and Cartesian coordinates of triazapentalenes (**1a**, **1b**, **1g**, **1e**, and **1f**). S16–S28

• ^{1}H NMR and ^{13}C NMR spectra S29–S58

General Method and Procedure.

All the reactions were carried out under an argon atmosphere. Tetrahydrofuran (THF) was freshly prepared by distillation from benzophenone ketyl before use. Triethylamine (TEA) was distilled from CaH_2 under argon atmosphere and stored over NaOH. Other anhydrous solvents and reagents were commercial grade and used as supplied.

NMR spectra were recorded on a JEOL JNM-ECA-500 (500 MHz) or a JEOL JNM-AL400 (400 MHz). Chemical shifts were reported in parts per million (ppm). For ^{1}H NMR spectra ($CDCl_3$ and CD_2Cl_2), tetramethylsilane and the residual solvent peak were used as the internal reference (0.00, 7.26 and 5.32 ppm), whereas the central solvent peak were used as the reference (77.0 and 39.5 ppm) for ^{13}C NMR spectra ($CDCl_3$ and DMSO-d_6). Mass spectra were recorded on a JEOL JMS-T-100GCV (FD) a Thermo Scientific Exactive (ESI) or a Waters Micromass LCT Premier (ESI). Infrared (IR) spectra were recorded on a JASCO FT/IR-4100 or a JASCO FT/IR-4200 spectrometer using NaCl plate. Analytical thin layer chromatography (TLC) was performed with E. Merck pre-coated TLC plates, silica gel 60F-254, layer thickness 0.25 mm. Flash chromatography was performed on Kanto Chemical 60 N (0.04–0.05 mm) mesh silica gel. Absorption spectra were recorded on a Hitachi U-3300 or a JASCO V-600 spectrometer and corrected fluorescence spectra were recorded on a Hitachi F-4500 or a JASCO FP-8200 spectrofluorometer. Sample solutions were degassed thoroughly by purging with an Ar gas stream for 30 min prior to the experiments and then sealed in their cells. Fluorescence quantum yields were estimated by using 9,10-diphenylanthracene (9,10-DPA) in cyclohexane ($\Phi_F = 0.91$) or rhodamine B in ethanol ($\Phi_F = 0.94$) as a standard.

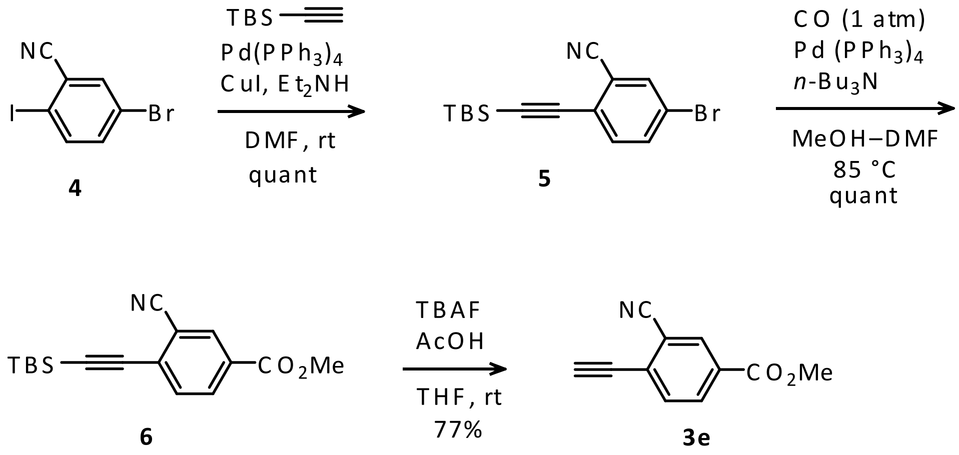

Scheme S1. Synthesis of **3e**

5-bromo-2-((*tert*-butyldimethylsilyl)ethynyl)benzonitrile (5)

To a solution of **4** (24.9 g, 81.0 mmol), TBS-acetylene (15.1 mL, 81.0 mmol), and Et_2NH (25.1 mL, 243 mmol) in anhydrous DMF (405 mL) were added CuI (771 mg, 4.05 mmol) and $Pd(PPh_3)_4$ (936 mg, 0.81 mmol) under Ar atmosphere at room temperature. After stirred for 16 h, the mixture was quenched with 1 M HCl at 0 °C and extracted with hexane (twice). The combined organic layers were washed with water and brine, dried over $MgSO_4$, filtrated, and concentrated under reduced pressure. The residue was purified by silica-gel column chromatography to give **5** (26.5 g, quant) as a yellow solid: mp 69–72 °C; ^{1}H NMR (500 MHz, $CDCl_3$) δ 7.75 (d, *J* = 2.0 Hz, 1H), 7.65 (dd, *J* = 8.5, 2.0 Hz, 1H), 7.42 (d, *J* = 8.0 Hz, 1H), 1.01 (s, 9H), 0.22 (s, 6H); ^{13}C NMR (125 MHz, $CDCl_3$) δ 135.4, 135.0, 133.6, 125.8, 122.0, 117.1, 115.9, 102.2, 100.0, 25.9, 16.5, –5.0; IR (neat) 3099, 3070, 2927, 2888, 2859, 2230, 2165, 1469, 1249, 825 cm^{-1}; HRMS (ESI) m/z $[M+Na]^+$ calcd for $[C_{15}H_{18}NBrSi+Na]^+$ 342.0284, found 342.0284.

Methyl 4-((*tert*-butyldimethylsilyl)ethynyl)-3-cyanobenzoate (6)

To a solution of **5** (13.9 g, 43.5 mmol) and *n*-Bu_3N (31 mL, 130 mmol) in MeOH (36 mL) and DMF (181 mL) was added $Pd(PPh_3)_4$ (2.5 g, 2.18 mmol) under Ar atmosphere at room temperature. After the flask was substituted with CO gas, the reaction mixture was heated to 85 °C for 14 h. After cooled to room temperature, the mixture was quenched with 1M HCl, diluted with hexane and filtrated through Celite. The filtrate was separated and the aqueous layer was extracted with hexane (x1). The combined organic layers were washed with water and brine, dried over $MgSO_4$, filtered, and concentrated under reduced pressure. The residue was purified by silica-gel column chromatography to give **6** (13.0 g, quant) as a yellow oil: ^{1}H NMR (500 MHz, $CDCl_3$) δ 8.29 (d, *J* = 1.5 Hz, 1H), 8.16 (dd, *J* = 8.5, 1.5 Hz, 1H), 7.64 (d, *J* = 9.0 Hz,

1H), 3.95 (s, 3H), 1.03 (s, 9H), 0.24 (s, 6H); ^{13}C NMR (125 MHz, $CDCl_3$) δ 164.6, 133.6, 132.9, 132.7, 130.9, 130.0, 116.5, 116.0, 104.6, 100.4, 52.7, 26.0, 16.6, –4.9; IR (neat) 3072, 3006, 2952, 2928, 2894, 2858, 2234, 2162, 1727, 1601, 1299, 1257, 828 cm^{-1}; HRMS (ESI) m/z $[M+Na]^+$ calcd for $[C_{17}H_{21}O_2NSi+Na]^+$ 322.1234, found 322.1233.

Methyl 3-cyano-4-ethynylbenzoate (3e)

To a solution of **6** (12.9 g, 43.1 mmol) and acetic acid (3.0 mL, 51.7 mmol) in THF (216 mL) was added TBAF (47.4 mL, 1 M solution in THF, 47.4 mmol) at room temperature. After stirred for 5 min, the mixture was diluted with water, extracted with ether (x2). The combined organic layers were washed with brine, dried over $MgSO_4$, filtered, and concentrated under reduced pressure. The residue was recrystalized from EtOAc to give **3e** (6.2 g, 77%) as orange crystals: mp 111–113 °C; ^{1}H NMR (500 MHz, $CDCl_3$) δ 8.30 (d, J = 1.5 Hz, 1H), 8.19 (dd, J = 8.0, 2.0 Hz, 1H), 7.68 (d, J = 8.0 Hz, 1H), 3.95 (s, 3H), 3.65 (s, 1H); ^{13}C NMR (125 MHz, $CDCl_3$) δ 164.5, 133.6, 133.1, 133.1, 130.7, 129.7, 116.3, 116.2, 86.8, 79.0, 52.8; IR (neat) 3249, 2236, 2110, 1714, 1298, 1287, 1258 cm^{-1}; HRMS (ESI) m/z $[M+H]^+$ calcd for $[C_{11}H_8NO_2]^+$ 186.0550, found 186.0552.

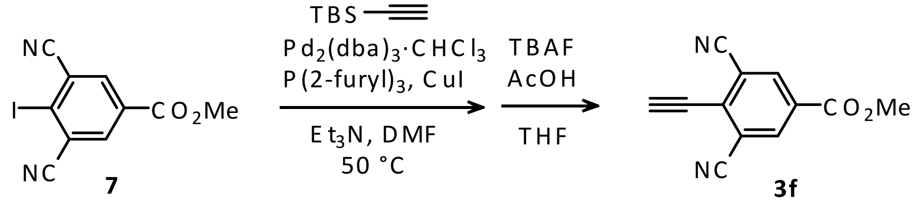

7 3f

Scheme S2. Synthesis of **3f**

Methyl 3,5-dicyano-4-ethynylbenzoate (3f)

To a solution of **7** (511 mg, 1.64 mmol), TBS-acetylene (460 μL, 2.46 mmol), and Et_3N (691 μL, 4.92 mmol) in anhydrous DMF (16.4 mL) were added CuI (62.5 mg, 0.328 mmol), P(2-furyl)$_3$ (76.2 mg, 0.328 mmol), $Pd_2(dba)_3{\cdot}CHCl_3$ (170 mg, 0.164 mmol) under Ar atmosphere at room temperature. After stirred at 50 °C for 2 h, the mixture was quenched with 1 M HCl at room temperature and extracted with ether (twice). The combined organic layers were washed with water (x2) and brine, dried over $MgSO_4$, bleached with activated charcoal, filtrated through Celite, and concentrated under reduced pressure. The crude product was dissolved in THF (16.4 mL). To the solution was added acetic acid (940 μL, 16.4 mmol) and TBAF (1.8 mL, 1 M solution in THF, 1.80 mmol). After stirred at room temperature for 30 min, the reaction mixture was quenched with 1 M HCl and extracted with ether (x3). The combined organic layers were washed with brine, dried over $MgSO_4$, filtered, and concentrated under reduced

pressure. The residue was recrystalized from EtOAc to give **3f** (192 mg, 56% for 2 steps) as orange crystals: dec. 157 °C; ^{1}H NMR (500 MHz, $CDCl_3$) δ 8.50 (s, 2H), 4.10 (s, 1H), 4.01 (s, 3H); ^{13}C NMR (125 MHz, $CDCl_3$) δ 136.7, 132.6, 131.5, 117.9, 114.8, 93.7, 76.1, 53.4; IR (neat) 3261, 3070, 2231, 2109, 1729, 1319, 1228 cm^{-1}; HRMS (FD) m/z $[M]^+$ calcd for $[C_{12}H_6N_2O_2]^+$ 210.0429, found 210.0434.

Typical procedure for the click reaction

To a solution of **2** (52.1 mg, 0.137 mmol) and alkyne **3** (0.114 mmol) in THF (11 mL) were added TEA (80.0 μL, 0.570 mmol), 570 μL of the ligand·copper solution (0.01 M, 5.70 μmol) at room temperature. The mixture was stirred for 3 h and concentrated under reduced pressure. The residue was purified by silica gel column chromatography to give **1**.

Preparation of ligand·copper solution

To a solution of bis[2-(*N*,*N*-dimethylaminoethyl)]ether (19.0 μL, 0.10 mmol) in THF (10 mL) was added copper(I) iodide (19 mg, 0.10 mmol) at room temperature. The mixture was stirred until homogeneous.

2-(3-cyanophenyl)-1,3a,6a-triazapentalene (1c)

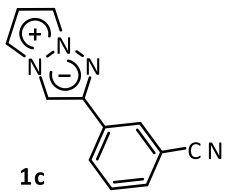

1c

Green solid; mp 118–123 °C (recrystalized from ether); ^{1}H NMR (500 MHz, $CDCl_3$) δ 8.04 (t, *J* = 1.8 Hz, 1H), 7.98 (ddd, *J* = 8.0, 1.7, 1.1 Hz, 1H), 7.59 (ddd, *J* = 8.0, 1.7, 1.2 Hz, 1H), 7.51 (t, *J* = 8.0 Hz, 1H), 7.44 (d, *J* = 2.3 Hz, 1H), 7.42 (s, 1H), 7.16 (d, *J* = 2.9 Hz, 1H), 6.65 (t, *J* = 2.9 Hz, 1H); ^{13}C NMR (125 MHz, $CDCl_3$) δ 145.3, 132.8, 131.4, 129.8, 129.4, 129.2, 118.6, 112.8, 109.2, 102.6, 101.4, 93.5; IR (neat) 3150, 2227, 1435, 1378, 1144, 969, 844 cm^{-1}; HRMS (ESI) m/z $[M+H]^+$ calcd for $[C_{12}H_8N_4]^+$ 209.0822, found 209.0824. UV/Vis (CH_2Cl_2): λ_{max} (log ε) = 327 (3.45), 282 (4.15), 274 (4.16), 260 (4.20) nm. FL (CH_2Cl_2): λ_{max} = 493 nm; Φ_F = 0.24 (reference to 9,10-DPA; excited at 370 nm).

2-(2-cyanophenyl)-1,3a,6a-triazapentalene (1d)

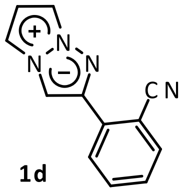

1d

Light green solid; mp 85–87 °C (recrystalized from ether); ^{1}H NMR (500 MHz, $CDCl_3$) δ 8.09 (dd, *J* = 8.0, 1.1 Hz, 1H), 7.93 (d, *J* = 1.2 Hz, 1H), 7.74 (dd, *J* = 8.0, 1.1 Hz, 1H), 7.66 (td, *J* = 8.0, 1.2 Hz, 1H), 7.46 (d, *J* = 2.9 Hz, 1H), 7.43 (td, *J* = 8.0, 1.2 Hz, 1H), 7.19 (d, *J* = 2.8 Hz, 1H), 6.68 (t, *J* = 2.9 Hz, 1H); ^{13}C NMR (125 MHz, $CDCl_3$) δ 143.4, 134.4, 133.9, 133.0, 128.4, 128.1, 119.1, 109.5, 109.0, 102.3, 101.4, 96.0; IR (neat) 3152, 2223, 1474, 1424, 949 cm^{-1}; HRMS (ESI) m/z $[M+Na]^+$ calcd for $[C_{12}H_8N_3O_2+Na]^+$ 231.0641, found 231.0642. UV/Vis

(CH_2Cl_2): λ_{max} (log ε) = 376 (3.11), 287 (4.19), ~270 (4.10), 260 (4.12) nm. FL (CH_2Cl_2): λ_{max} = 515 nm; Φ_F = 0.24 (reference to 9,10-DPA; excited at 370 nm).

2-(4-(methoxycarbonyl)phenyl)-1,3a,6a-triazapentalene (1g)

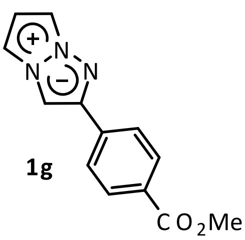

1g

Green solid; mp 148–153 °C; ^{1}H NMR (500 MHz, $CDCl_3$) δ 8.09 (d, *J* = 8.6 Hz, 2H), 7.84 (d, *J* = 8.6 Hz, 2H), 7.47 (s, 1H), 7.45 (d, *J* = 2.8 Hz, 1H), 7.15 (d, *J* = 2.8 Hz, 1H), 6.64 (t, *J* = 2.9 Hz, 1H), 3.94 (s, 1H); ^{13}C NMR (125 MHz, $CDCl_3$) δ 166.8, 146.4, 135.8, 130.0. 129.6, 125.5, 109.2, 102.4, 101.1, 94.0, 52.1; IR (neat) 3152, 3134, 3120, 1710, 1433, 1417, 1280, 1104 cm^{-1}; HRMS (ESI) m/z $[M+Na]^+$ calcd for $[C_{13}H_{11}N_3O_2+Na]^+$ 264.0744, found 264.0745. UV/Vis (CH_2Cl_2): λ_{max} (log ε) = 376 (3.09), 287 (4.14) nm. FL (CH_2Cl_2): λ_{max} = 521 nm; Φ_F = 0.44 (reference to 9,10-DPA; excited at 370 nm).

2-(2-cyano-4-(methoxycarbonyl)phenyl) -1,3a,6a-triazapentalene (1e)

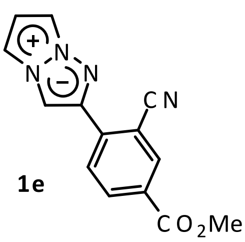

1e

Light brown powder; ^{1}H NMR (500 MHz, $CDCl_3$) δ 8.40 (d, *J* = 1.5 Hz, 1H), 8.28 (dd, *J* = 8.5, 1.5 Hz, 1H), 8.22 (d, *J* = 8.5 Hz, 1H), 8.06 (d, *J* = 1.0 Hz, 1H), 7.48 (d, *J* = 2.5 Hz, 1H), 7.23 (d, *J* = 3.0 Hz, 1H), 6.71 (dd, *J* = 3.0, 2.5 Hz, 1H), 3.97 (s, 3H); ^{13}C NMR (125 MHz, $CDCl_3$) δ 164.9, 142.4, 138.2, 135.2, 133.7, 129.8, 128.5, 118.3, 110.0, 109.0, 102.5, 101.8, 96.7, 52.6; IR (neat) 3170, 3155, 2226, 1716, 1296 cm^{-1}; HRMS (ESI) m/z $[M+Na]^+$ calcd for $[C_{14}H_{10}N_4O_2+Na]^+$ 289.0696, found 289.0698. UV/Vis (CH_2Cl_2): λ_{max} (log ε) = 420 (2.80), 285 (3.98) nm. FL (CH_2Cl_2): λ_{max} = 572 nm; Φ_F = 0.34 (reference to rhodamine B; excited at 400 nm).

2-(2,6-dicyano-4-(methoxycarbonyl)phenyl) -1,3a,6a-triazapentalene (1f)

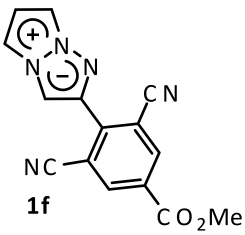

1f

Dark brown solid; dec. 195 °C; ^{1}H NMR (500 MHz, $CDCl_3$) δ 8.61 (s, 2H), 7.80 (s, 2H), 7.59 (d, *J* = 2.0 Hz, 1H), 7.30 (d, *J* = 2.5 Hz, 1H), 6.77 (t, *J* = 2.5 Hz, 1H), 4.03 (s, 3H); ^{13}C NMR (125 MHz, $CDCl_3$) δ 163.1, 140.5, 139.4, 138.5, 130.9, 116.3, 113.9, 110.3, 103.4, 102.2, 97.4, 53.3; IR (neat) 3179, 3158, 3141, 3064, 2231, 1732, 1717, 1558, 1541, 1521, 1507, 1315, 1232 cm^{-1}; HRMS (ESI) m/z $[M+Na]^+$ calcd for $[C_{15}H_9N_5O_2+Na]^+$ 314.0648, found 314.0648. UV/Vis (CH_2Cl_2): λ_{max} (log ε) = 466 (3.20), 286 (4.42) nm. FL (CH_2Cl_2): λ_{max} = 632 nm; Φ_F = 0.096 (reference to rhodamine B; excited at 430 nm).

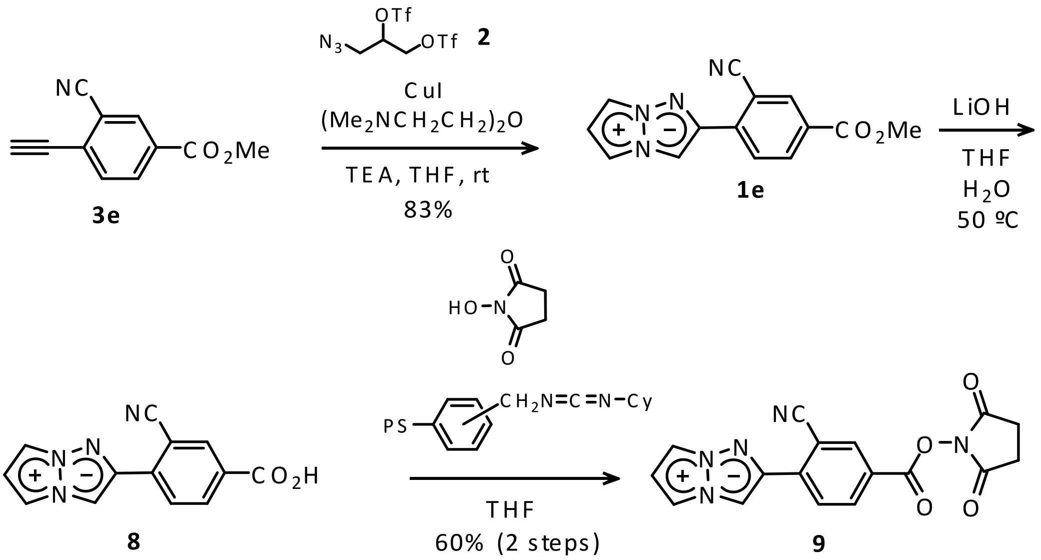

Scheme S3. Synthesis of *N*-succinimidyl ester **11**

Typical procedure for large-scale synthesis of 1e.

To a solution of azide **2** (5.10 g, 13.4 mmol) and alkyne **3e** (2.25 g, 12.2 mmol) in THF (610 mL) were added the homogeneous solution of CuI (116 mg, 0.610 mmol), bis [2-(*N*,*N*-dimethylaminoethyl)]ether (115 μL, 0.610 mmol) and Et_3N (8.6 mL, 61.0 mmol) in THF (61 mL) at room temperature. After the resulting mixture was stirred for 24 h, the solvent was removed to less than one third under reduced pressure and then aq 5% NH_3 was poured to precipitate a powder. The powder was separated by filtration and dried *in vacuo* to afford **1e** (2.7 g, 83%) as a light brown powder.

2-(2-cyano-4-(((2,5-dioxopyrrolidin-1-yl)oxy)carbonyl)phenyl)-1,3a,6a-triaza-pentalene (9)

To a solution of **1e** (2.70 g, 10.1 mmol) in H_2O (50 mL) and THF (101 mL) was added $LiOH{\cdot}H_2O$ (510 mg, 12.1 mmol) at room temperature. After stirred at 50 °C for 30 min, the mixture was washed with ether (x1), acidified by 1M HCl to participate yellow solid. The solid was dissolved in THF/EtOAc mixture and the organic layer was separated. After the aqueous layer was extracted with THF/EtOAc (x2), the combined organic layers were dried over $MgSO_4$, filtered, and concentrated *in vacuo*. The residue was dehydrated by toluene azeotropy and dissolved in THF (54 mL). To the mixture were added *N*-hydroxysuccinimide (1.23 g, 10.7 mmol) and *N*-cyclohexylcarbodiimidomethyl polystylene resin (8.7 g, 13.0 mmol, TCI No. C2141,

1.5 mmol/g loading) at room temperature. After the reaction mixture was stirred for 1 h, solid materials were removed by filtration and the residue was washed with THF. The filtrate was concentrated *in vacuo* to form red powder. This powder was dispersed to hexane, filtered, washed with ether and CH_2Cl_2 to give **11** (2.1 g, 56% for 2 steps) as a red powder: dec. 200 °C; ^{1}H NMR (500 MHz, CD_2Cl_2) δ 8.50 (d, J = 1.5 Hz, 1H), 8.35 (dd, J = 8.0, 1.5 Hz, 1H), 8.31 (d, J = 8.5 Hz, 1H), 8.11 (s, 1H), 7.51 (d, J = 2.5 Hz, 1H), 7.29 (d, J = 3.0 Hz, 1H), 6.75 (dd, J = 3.0, 2.5 Hz, 1H), 2.91 (brs, 4H); ^{13}C NMR (100 MHz, DMSO-d_6) δ 170.1, 160.2, 141.4, 139.5, 136.0, 134.2, 129.2, 124.1, 117.4, 110.5, 109.1, 103.3, 102.9, 97.4, 25.5; IR (neat) 3181, 3141, 2225, 1773, 1737, 1200 cm^{-1}; HRMS (ESI) m/z $[M+Na]^+$ calcd for $[C_{17}H_{12}N_5O_4+Na]^+$ 350.0884, found 350.0886. UV/Vis (CH_2Cl_2): λ_{max} (log ε) = 446 (3.38), 287 (4.47) nm. FL (CH_2Cl_2): λ_{max} = 624 nm (fluorescence quantum yield was not estimated because of gradual degradation of **9** under the measurement condition.)

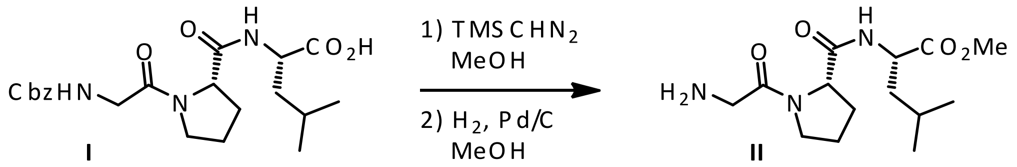

Scheme S4. Synthesis of tripeptide **II**

To a solution of **I** (104.9 mg, 0.250 mmol) in MeOH (2.5 mL) was added $TMSCHN_2$ (1.3 mL, 2.60 mmol, 2.0 M ether solution) at room temperature. After stirred for 1 h, the mixture was concentrated *in vacuo*. The residue was dissolved in MeOH (2.5 mL) and to the resulting solution was added 10% Pd/C (12 mg). After stirred for 24 h under H_2 atmosphere, the suspension was diluted with $CHCl_3$, filtered through Celite, and concentrated *in vacuo*. The residue was purified by silica-gel column chromatography to give **II** (86.7 mg, quant) as a colorless amorphous solid: ^{1}H NMR (400 MHz, $CDCl_3$) δ 7.96 (d, J = 7.2 Hz, 1H), 4.65 (dd, J = 7.6, 2.4 Hz, 1H), 4.42 (ddd, J = 10.0, 7.6, 5.2 Hz, 1H), 4.13 (d, J = 16.0 Hz, 1H), 3.98 (d, J = 15.6 Hz, 1H), 3.71 (s, 3H), 3.73–3.60 (m, 1H), 3.51 (dd, J = 16.8, 8.8 Hz, 1H), 2.23–1.85 (m, 5H), 1.72–1.50 (m, 2H), 0.93 (d, J = 6.4 Hz, 3H), 0.88 (d, J = 6.4 Hz, 3H); ^{13}C NMR (400 MHz, $CDCl_3$) δ 174.0, 172.1, 167.1, 60.8, 52.4, 51.4, 46.8, 41.8, 39.4, 29.8, 25.0, 24.3, 22.8, 21.4; IR (neat) 3148 (br), 3054, 2958, 2874, 1740, 1654, 1540, 1228, 1209, 669 (br) cm^{-1}; HRMS (ESI) m/z $[M+Na]^+$ calcd for $[C_{14}H_{25}N_3O_4+Na]^+$ 322.1743, found 322.1743.

Condensation of amines with 9

Labeled glycine ethyl ester 10

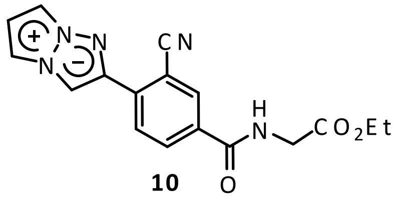

10

To a solution of glycine ethyl ester hydrochloride (5.5 mg, 0.0394 mmol), Et_3N (27.7 μL, 0.197 mmol) in DMF (80 μL) was added **9** (15.2 mg, 0.0451 mmol) at room temperature. After stirred for 15 min, the mixture was filtered through silica-gel pad ($CHCl_3$/EtOAc = 1:1). The filtrate was concentrated under reduced pressure and contaminated DMF was removed by azeotrope with toluene to afford pure **12** (12.6 mg, 95%) as a yellow amorphous powder: ^{1}H NMR (400 MHz, $CDCl_3$) δ 8.22 (d, J = 6.8 Hz, 1H), 8.20 (s, 1H), 8.05 (dd, 1H), 8.04 (d, J = 1.2 Hz), 7.48 (d, J = 2.8 Hz, 1H), 7.22 (d, J = 3.2 Hz, 1H), 6.78 (brt, 1H), 6.71 (t, J = 2.8 Hz, 1H), 4.29 (q, J = 7.2 Hz, 2H), 4.26 (d, J = 4.8 Hz, 2H), 1.33 (t, J = 7.2 Hz, 3H); ^{13}C NMR (100 MHz, $CDCl_3$) δ 169.8, 164.9, 142.4, 137.3, 133.3, 133.0, 131.2, 128.7, 118.3, 109.9, 109.2, 102.5, 101.8, 96.6, 61.9, 42.0, 14.1; IR (neat) 3394, 3343, 3158, 2982, 2932, 2228, 1747, 1651, 1608, 1540, 1511, 1208 cm^{-1}; HRMS (ESI) m/z $[M+Na]^+$ calcd for $[C_{17}H_{15}N_5O_3+Na]^+$ 360.1073, found 360.1072. UV/Vis (CH_2Cl_2): λ_{max} (log ε) = 387 (3.40), 284 (4.30) nm. FL (CH_2Cl_2): λ_{max} = 563 nm; Φ_F = 0.37 (reference to rhodamine B; excited at 400 nm).

Labeled tripeptide 11

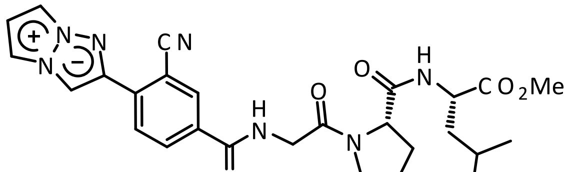

11

To a solution of tripeptide **II** (11.0 mg, 0.0367 mmol), Et_3N (25.8 μL, 0.184 mmol) in DMF (73 μL) was added **9** (12.8 mg, 0.0367 mmol) at room temperature. After stirred for 10 min, the mixture was directly purified by silica-gel column chromatography to give **11** (16.1 mg, 82%) as a yellow amorphous solid: ^{1}H NMR (400 MHz, $CDCl_3$) δ 8.24 (s, 1H), 8.18 (d, J = 8.4 Hz, 1H), 8.06 (d, J = 8.0 Hz, 1H), 8.02 (s, 1H), 7.48 (d, J = 2.8 Hz, 1H), 7.45 (brs, 1H), 7.22 (d, J = 2.8 Hz, 1H), 6.86 (d, J = 8.0 Hz, 1H), 6.70 (t, J = 2.8 Hz, 1H), 4.64–4.50 (m, 2H), 4.30 (dd, J = 17.6, 4.4 Hz, 1H), 4.22 (dd, J = 18.0, 4.0 Hz, 1H), 3.74 (s, 3H), 3.73–3.63 (m, 1H), 3.54 (dd, J = 15.6, 8.0 Hz, 1H), 2.40–2.28 (m, 1H), 2.26–1.95 (m, 3H), 1.82–1.50 (m, 3H), 0.92 (d, J = 5.6 Hz, 3H), 0.92 (d, J = 5.2 Hz, 3H); ^{13}C NMR (100 MHz, $CDCl_3$) δ 173.2, 170.5, 167.9, 164.7, 142.5, 137.2, 133.3, 133.1, 131.2, 128.6, 118.4, 109.9, 109.2, 102.5, 101.8, 96.6, 60.3, 52.3, 51.1, 46.5, 42.6, 41.3, 28.0, 25.0, 24.8, 22.7, 22.0; IR (neat) 3307, 3154, 3069, 2956,

2228, 1742, 1646, 1541, 1314, 1203 cm^{-1}; HRMS (ESI) m/z $[M+Na]^+$ calcd for $[C_{27}H_{31}N_7O_5+Na]^+$ 556.2284, found 556.2283. UV/Vis (CH_2Cl_2): λ_{max} (log ε) = 379 (3.34), 283 (4.26) nm. FL (CH_2Cl_2): λ_{max} = 561 nm; Φ_F = 0.24 (reference to rhodamine B; excited at 400 nm).

2-(4-(methoxycarbonyl)phenyl) -4-phenyl-1,3a,6a-triazapentalene (III)

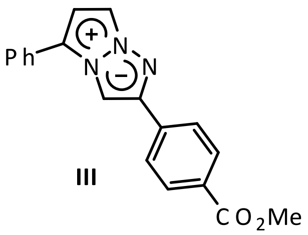

III

Green solid; mp. 124–127 °C; ^{1}H NMR (400 MHz, $CDCl_3$) δ 8.11 (d, *J* = 10.8 Hz, 2H), 7.90 (d, *J* = 10.8 Hz, 2H), 7.86 (s, 1H), 7.60 (d, *J* = 10.0 Hz, 2H), 7.58 (d, *J* = 3.6 Hz, 1H), 7.49 (t, *J* = 10.0 Hz, 2H), 7.32–7.24 (m, 1H), 6.88 (d, *J* = 4.0 Hz, 1H), 3.94 (s, 3H); ^{13}C NMR (125 MHz, $CDCl_3$) δ 166.7, 147.0, 135.6, 130.0, 129.8, 129.7, 129.2, 126.3, 125.6, 123.4, 115.5, 106.7, 104.4, 94.5, 52.1; IR (neat) 3155, 1950, 1715, 1277 cm^{-1}; HRMS (ESI) m/z $[M+Na]^+$ calcd for $[C_{19}H_{15}N_3O_2Na]^+$ 340.1062, found 340.1064. UV/Vis (CH_2Cl_2): λ_{max} (log ε) = 345 (4.35), 279 (4.44) nm. FL (CH_2Cl_2): λ_{max} = 548 nm; Φ_F = 0.36 (reference to rhodamine B; excited at 400 nm).

2-(2-cyano-4-(methoxycarbonyl)phenyl) -4-phenyl1,3a,6a-triazapentalene (IV)

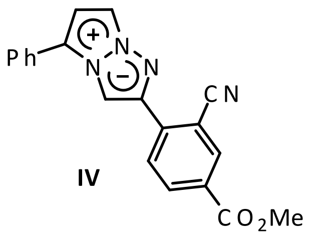

IV

Orange solid; dec. 216 °C; ^{1}H NMR (400 MHz, $CDCl_3$) δ 8.44 (s, 1H), 8.39 (s, 1H), 8.31 (d, *J* = 8.0 Hz, 1H), 8.24 (d, *J* = 8.0 Hz, 1H), 7.66–7.59 (m, 3H), 7.50 (t, *J* = 7.6 Hz, 2H), 7.30 (t, *J* = 7.6 Hz, 1H), 6.95 (d, *J* = 2.8 Hz, 1H), 3.98 (s, 3H); ^{13}C NMR (125 MHz, $CDCl_3$) δ 164.9, 143.1, 138.0, 135.3, 133.8, 130.2, 129.4, 129.4, 128.7, 126.8, 123.7, 118.3, 116.3, 109.3, 107.6, 104.5, 96.9, 52.7; IR (neat) 3155, 2960, 2228, 1733, 1300 cm^{-1}; HRMS (ESI) m/z $[M+H]^+$ calcd for $[C_{20}H_{15}N_4O_2]^+$ 343.1195, found 343.1198. UV/Vis (CH_2Cl_2): λ_{max} (log ε) = 432 (3.66), 336 (4.58), 282 (4.48) nm. FL (CH_2Cl_2): λ_{max} = 613 nm; Φ_F = 0.070 (reference to rhodamine B; excited at 400 nm).

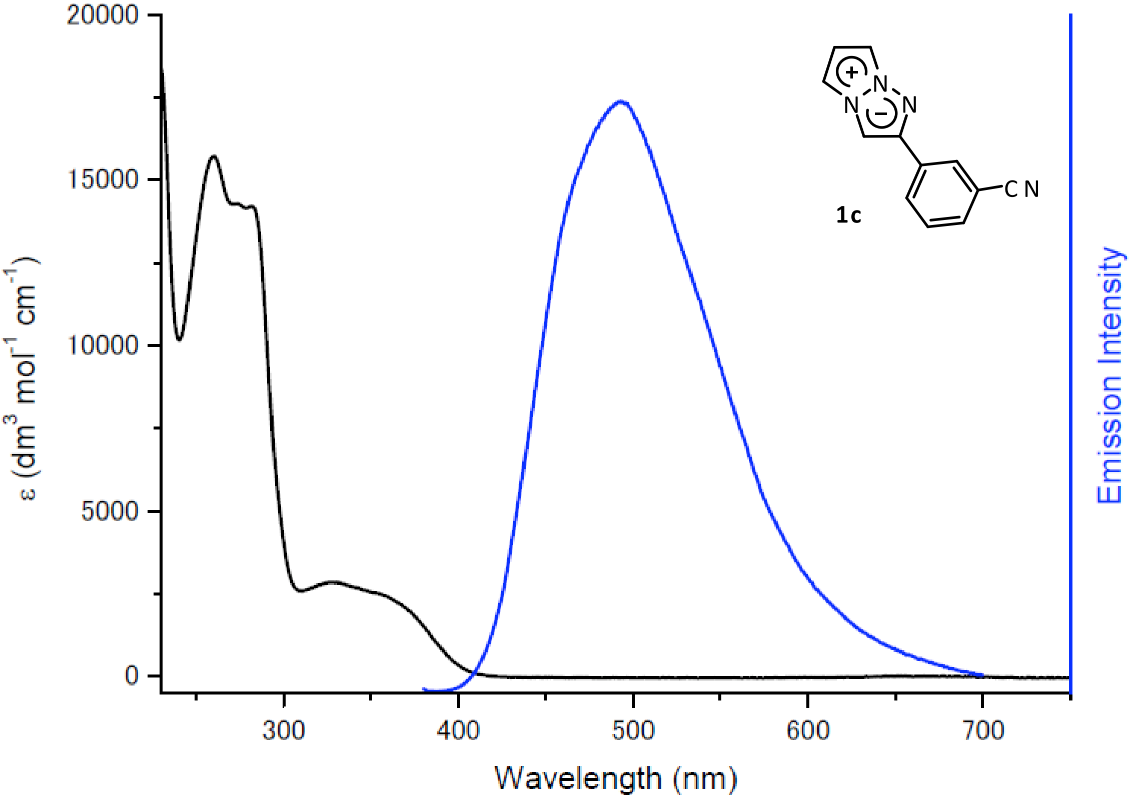
1c
CN
ε (dm^3 mol^{-1} cm^{-1})
Emission Intensity
Wavelength (nm)

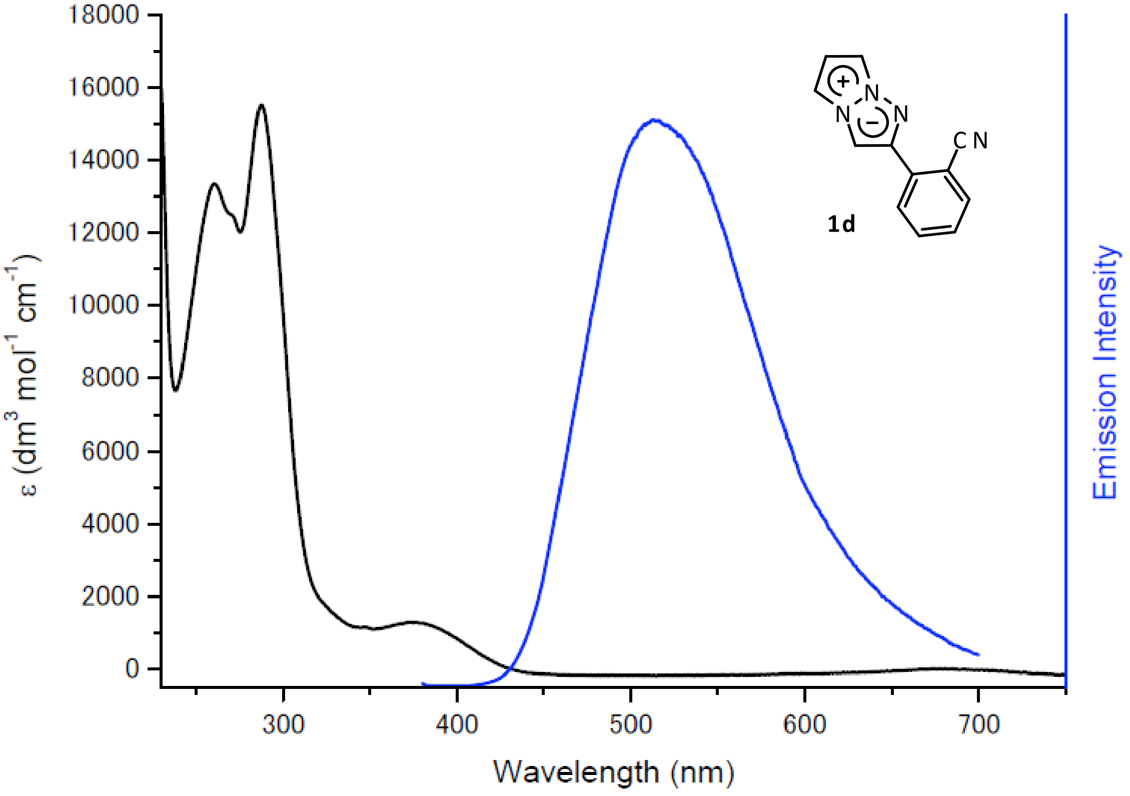
1d
CN
ε (dm^3 mol^{-1} cm^{-1})
Emission Intensity
Wavelength (nm)

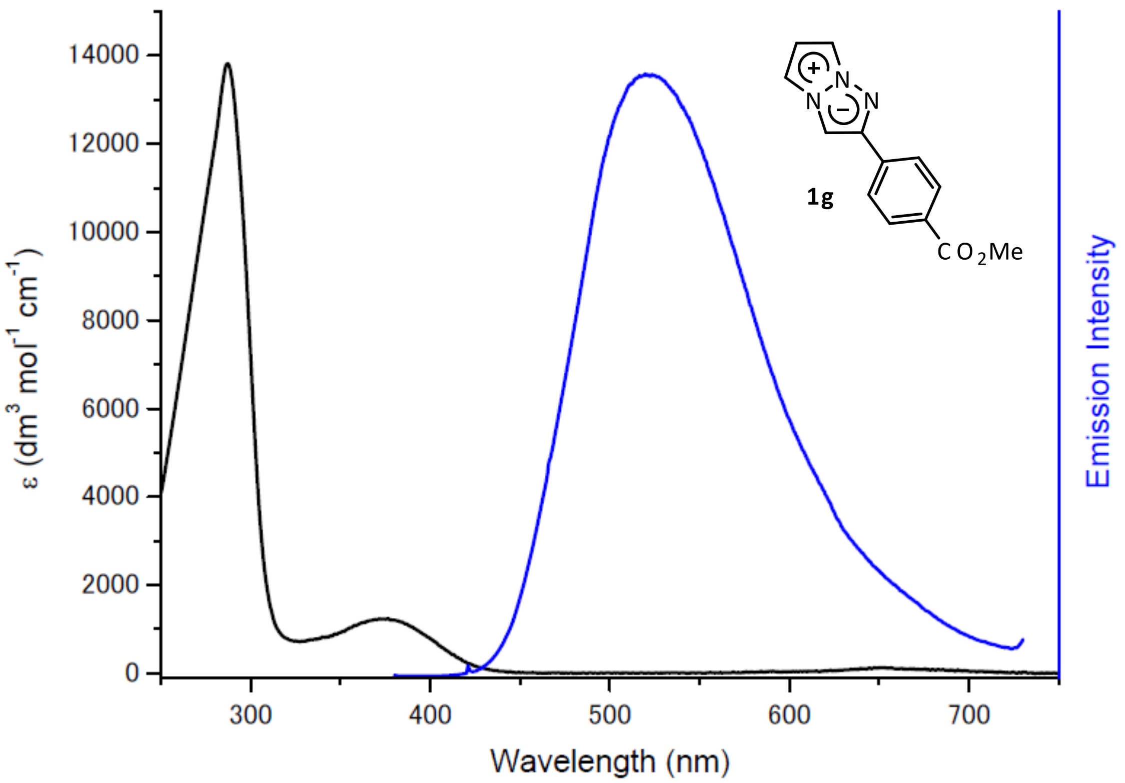
14000
12000
10000
8000
6000
4000
2000
0
300
400
500
600
700
Wavelength (nm)
ε (dm^3 mol^{-1} cm^{-1})
Emission Intensity
1g
CO$_2$Me

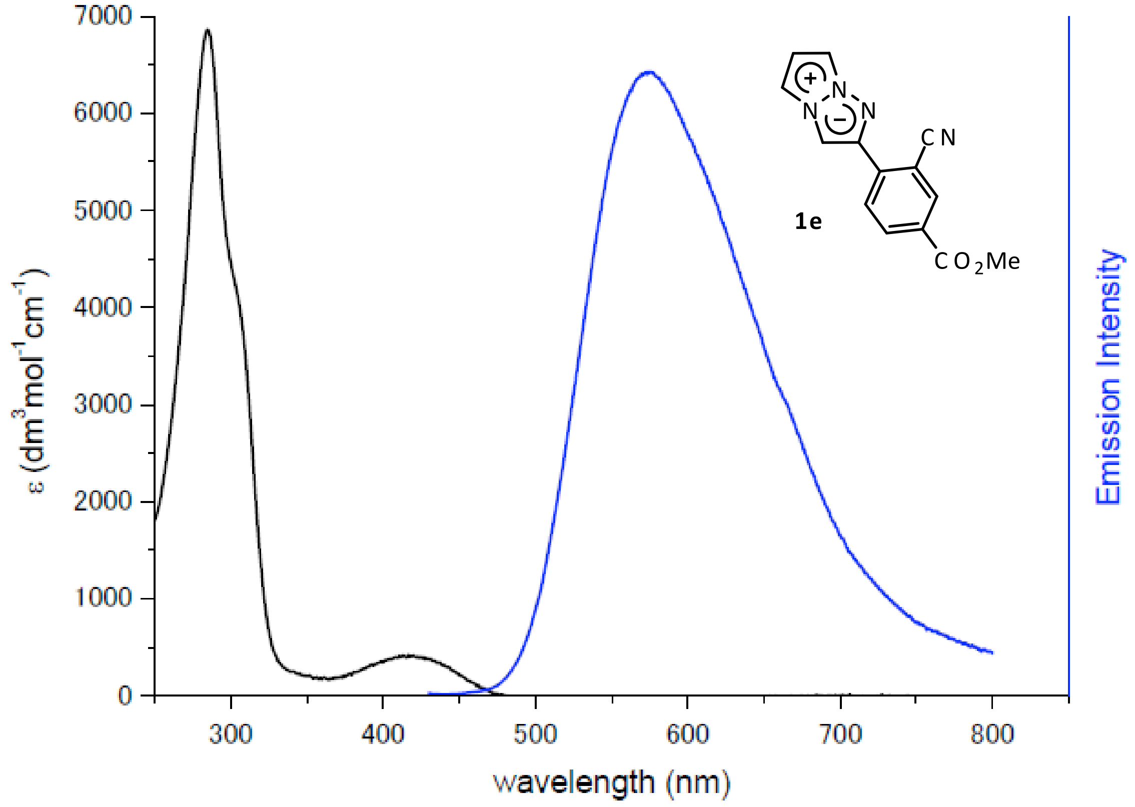
7000
6000
5000
4000
3000
2000
1000
0
300
400
500
600
700
800
wavelength (nm)
ε (dm^{3}mol^{-1}cm^{-1})
Emission Intensity
1e
CN
CO$_2$Me

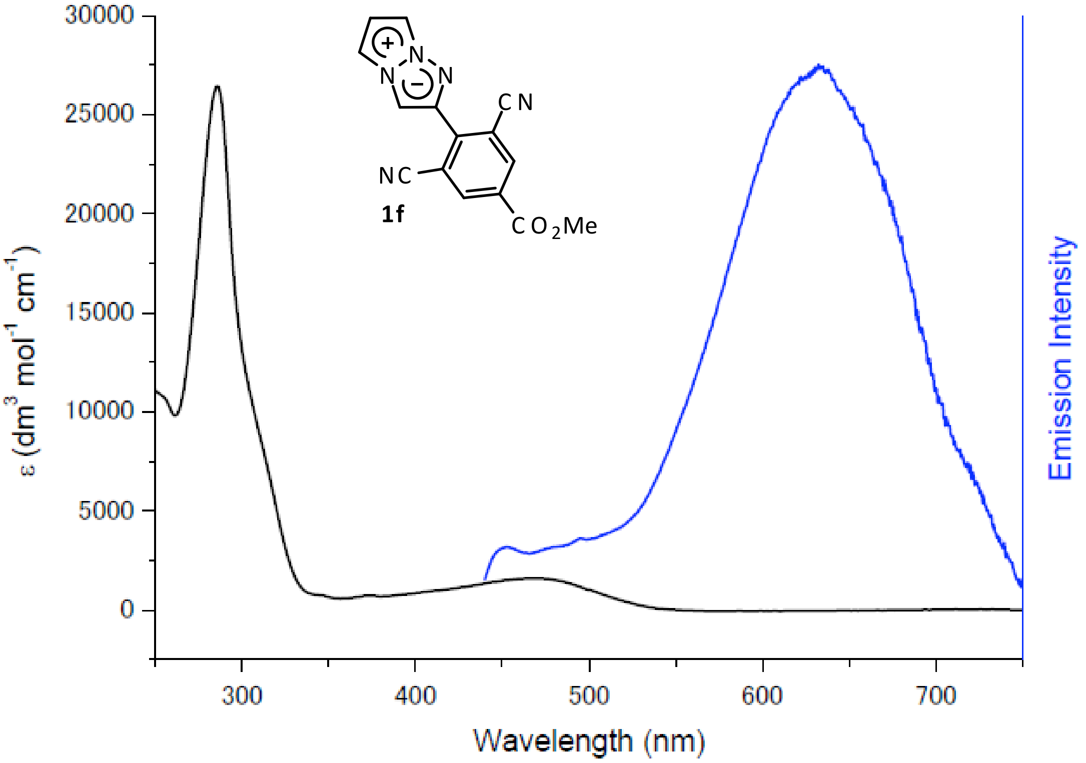
30000
25000
20000
15000
10000
5000
0
300
400
500
600
700
Wavelength (nm)
ε (dm3 mol-1 cm-1)
Emission Intensity
N
N
N
CN
NC
1f
CO2Me

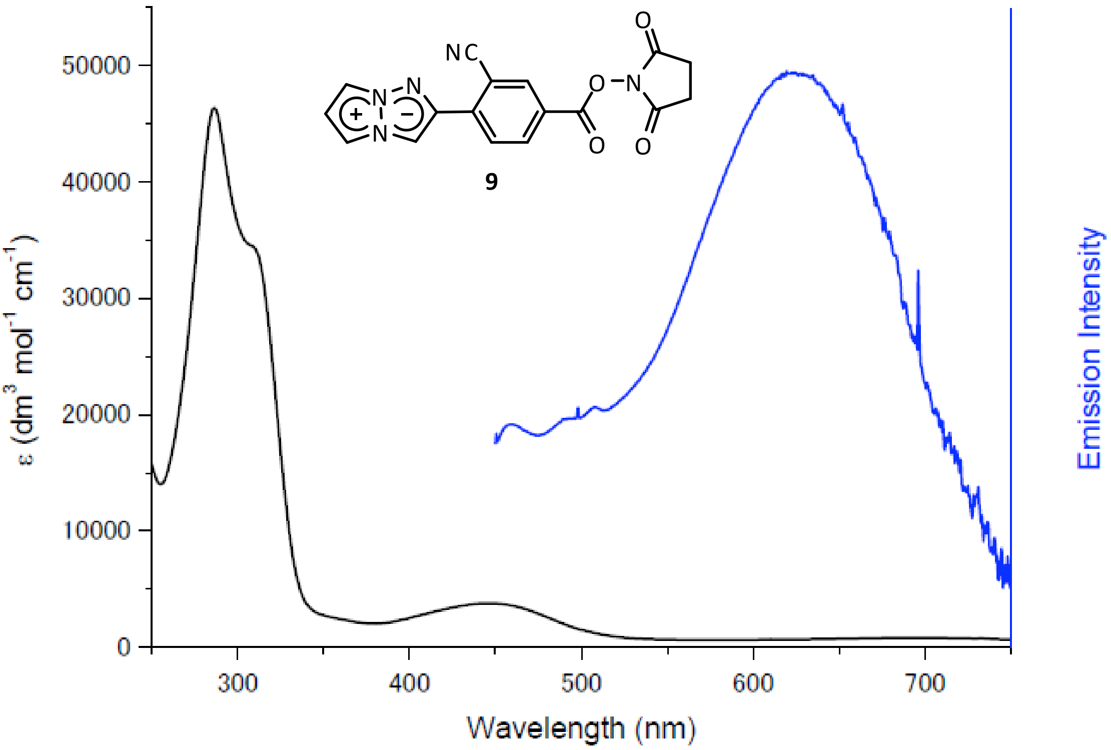
50000
40000
30000
20000
10000
0
300
400
500
600
700
Wavelength (nm)
ε (dm3 mol-1 cm-1)
Emission Intensity
NC
O
O
N
O
O
9

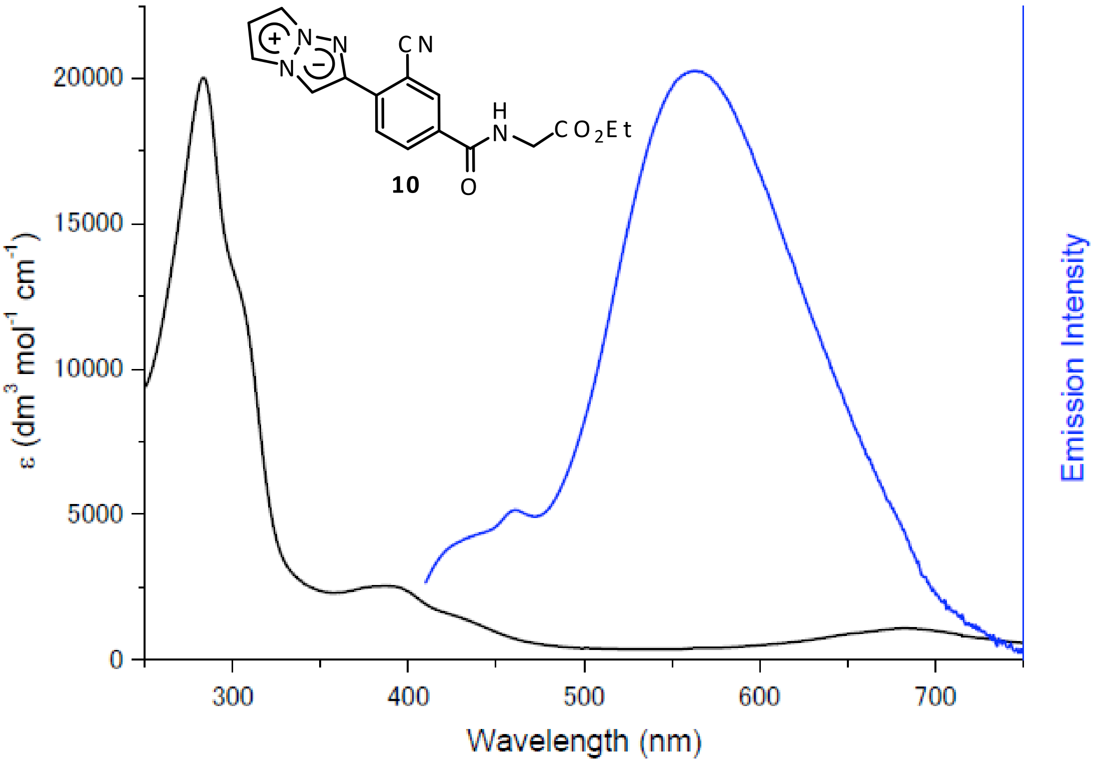

CN
H
N
CO2Et
10
O
ε (dm3 mol-1 cm-1)
20000
15000
10000
5000
0
300
400
500
600
700
Wavelength (nm)
Emission Intensity

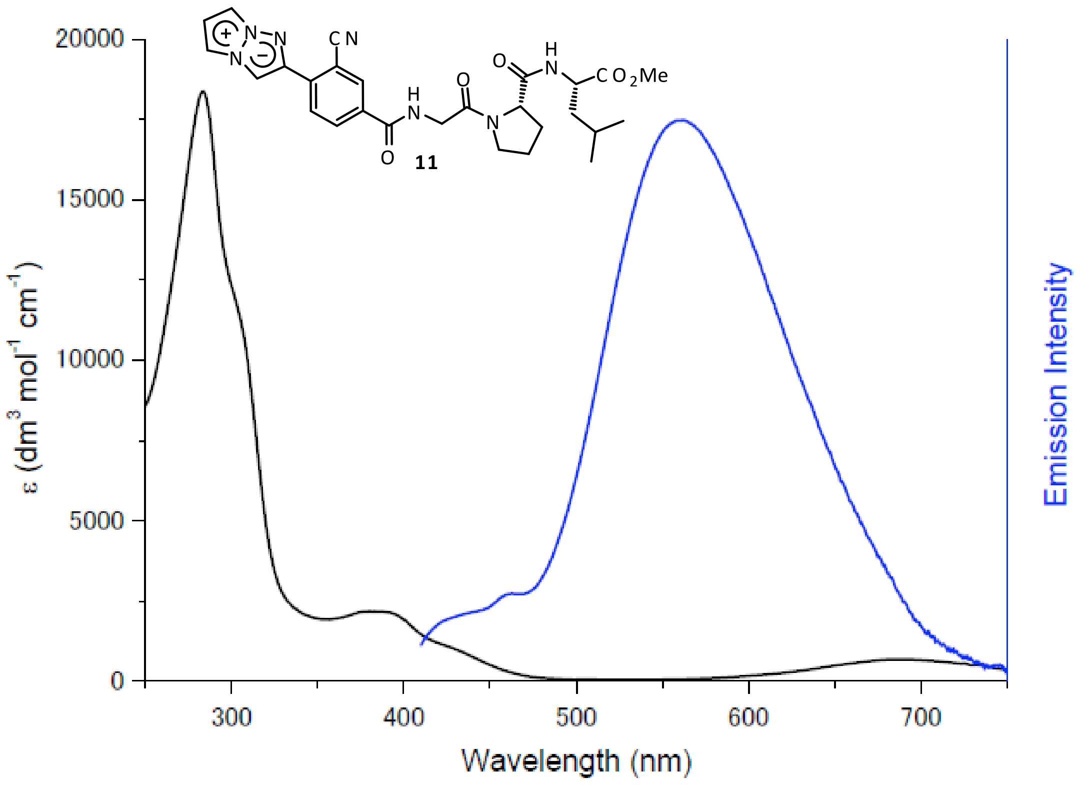

CN
H
N
O
N
H
N
CO2Me
O
11
ε (dm3 mol-1 cm-1)
20000
15000
10000
5000
0
300
400
500
600
700
Wavelength (nm)
Emission Intensity

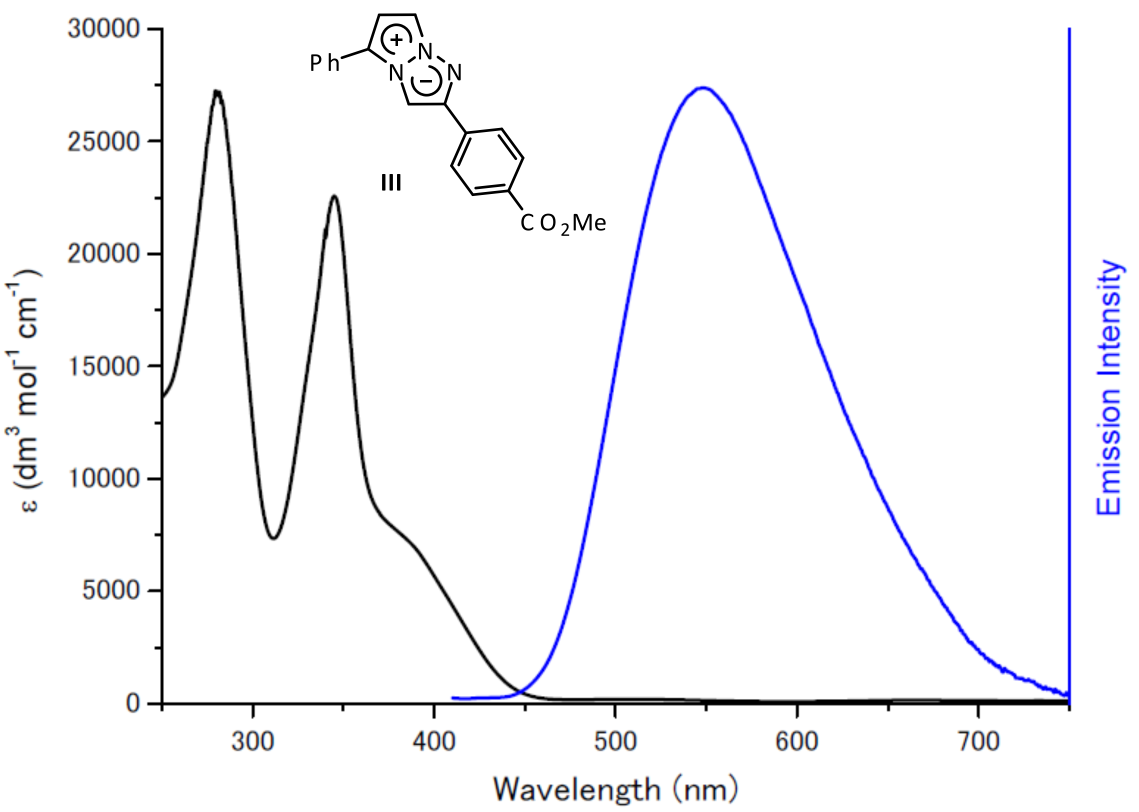
III
Ph
N
N
N
CO2Me
ε (dm3 mol-1 cm-1)
Emission Intensity
Wavelength (nm)
30000
25000
20000
15000
10000
5000
0
300
400
500
600
700

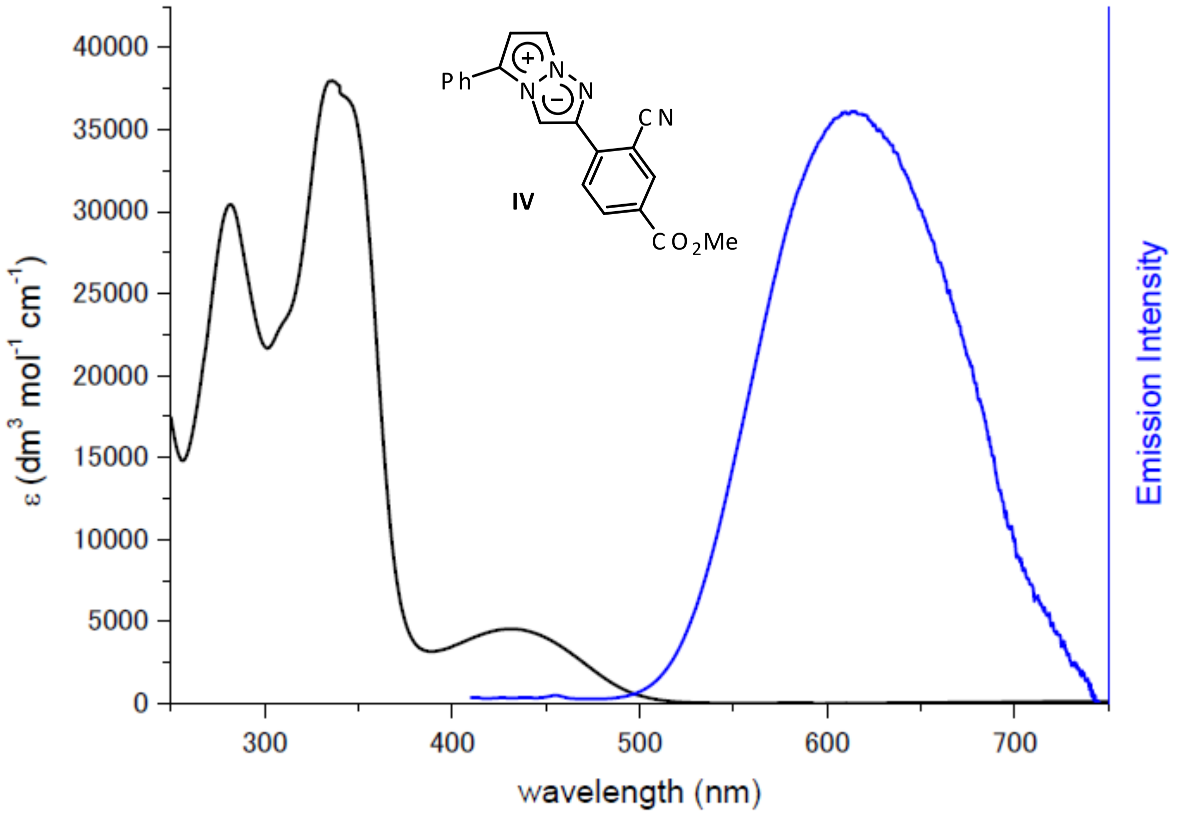
IV
Ph
N
N
N
CN
CO2Me
ε (dm3 mol-1 cm-1)
Emission Intensity
wavelength (nm)
40000
35000
30000
25000
20000
15000
10000
5000
0
300
400
500
600
700

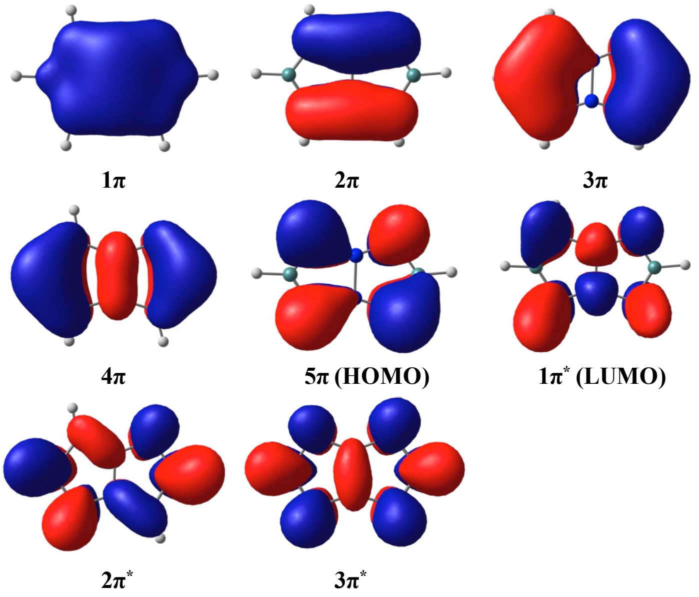

Figure S1. Active orbitals of **1a** at $(S_0)_{min}$ in the SA-CASSCF(10,8) calculation.

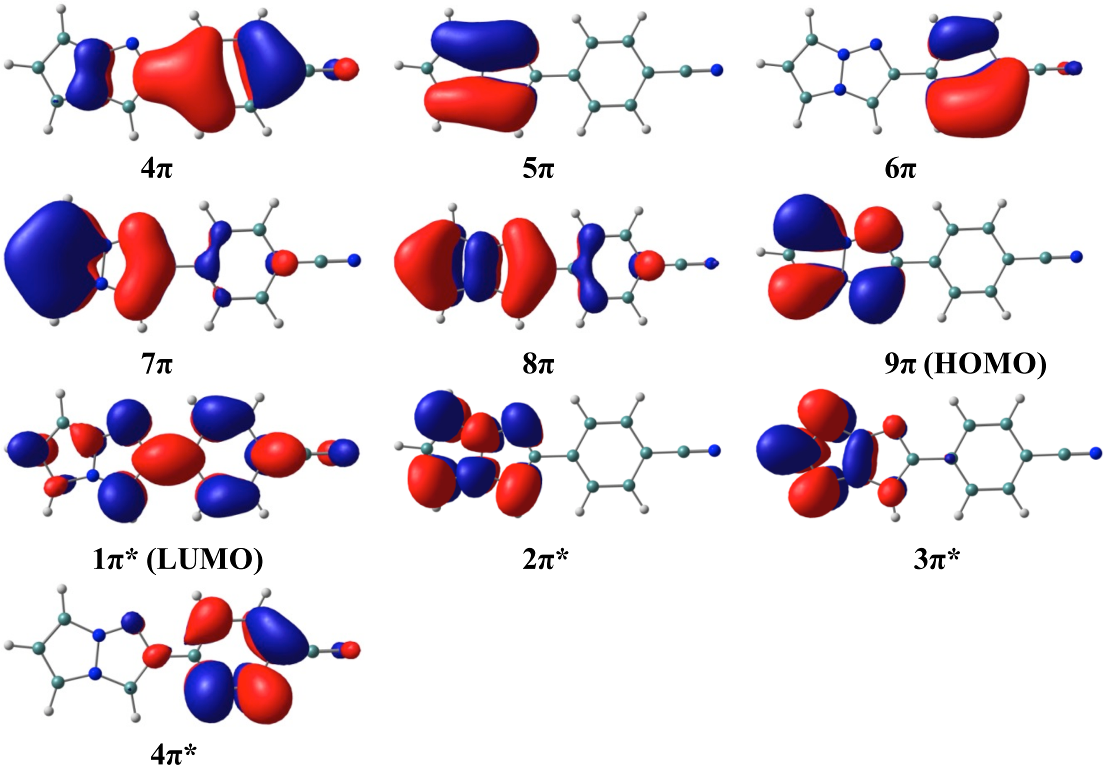

Figure S2. Active orbitals of **1b** at $(S_0)_{min}$ in the SA-CASSCF(12,10) calculation.

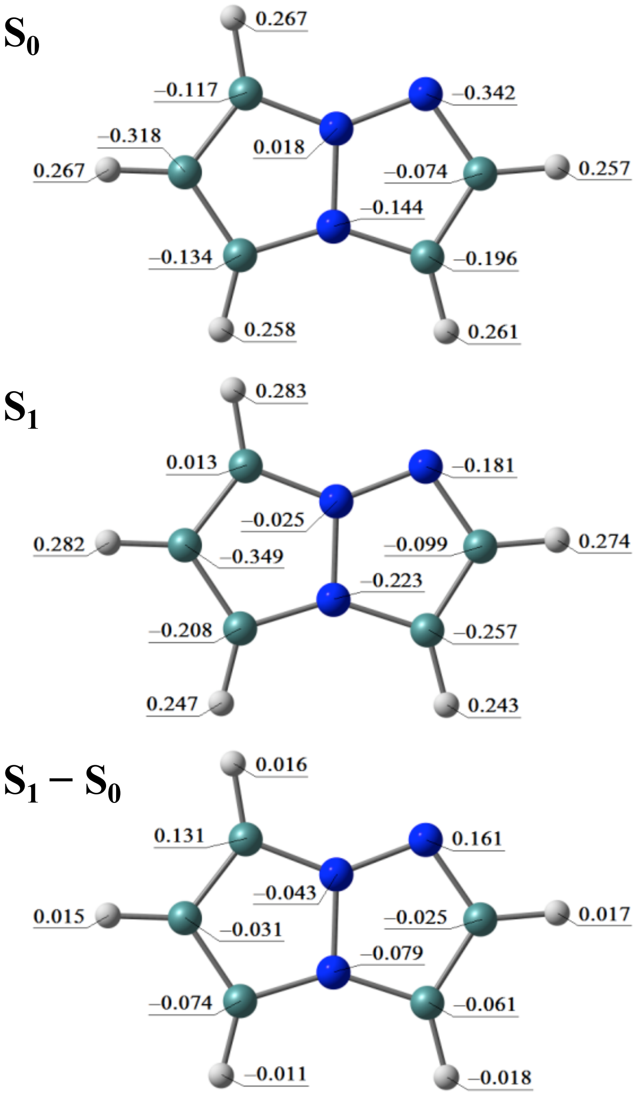

Figure S3. Natural charges of **1a** in the S_0 and S_1 states and their differences at $(S_0)_{min}$ in gas phase.

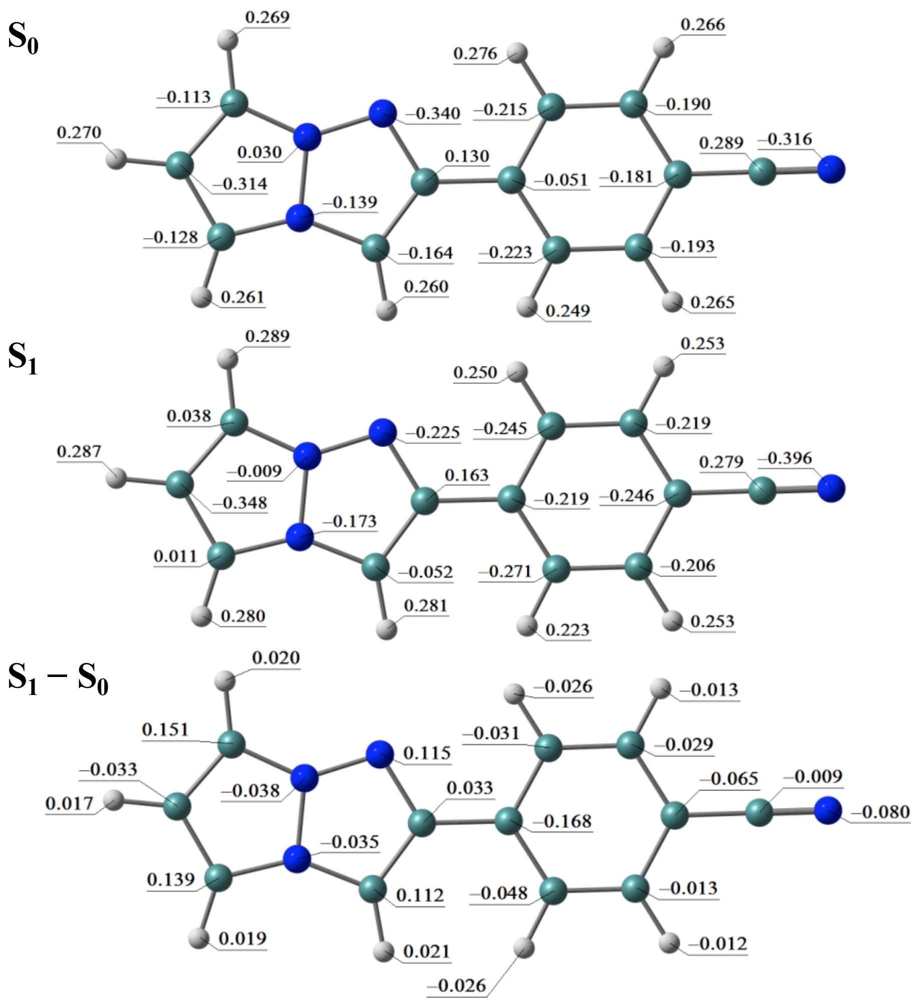

Figure S4. Natural charges of **1b** in the S_0 and S_1 states and their differences at $(S_0)_{min}$ in gas phase.

Table SI. Sum of the Natural Charges in the 1,3a,6a-Triazapentalene Skeleton and Dipole Moments (in Debye) in the S_0 and S_1 States at $(S_0)_{min}$ and $(S_1)_{min}$ in the Gas Phase.

	Charge (S_0)	Charge (S_1)	μ (S_0)	μ (S_1)
**1a**				
at $(S_0)_{min}$	–	–	2.50	4.67
at $(S_1)_{min}$	–	–	2.44	1.15
**1b**				
at $(S_0)_{min}$	0.022	0.542	7.11	19.47
at $(S_1)_{min}$	0.026	0.507	6.96	18.34
**1g**				
at $(S_0)_{min}$	0.013	0.527	2.68	15.66
at $(S_1)_{min}$	0.019	0.501	2.93	14.97
**1e**				
at $(S_0)_{min}$	0.049	0.639	4.36	15.47
at $(S_1)_{min}$	0.053	0.621	4.15	14.71
**1f**				
at $(S_0)_{min}$	0.066	0.730	1.07	13.57
at $(S_1)_{min}$	0.064	0.703	0.68	12.74

Table SII. Sum of the Natural Charges in the 1,3a,6a-Triazapentalene Skeleton and Dipole Moments (in Debye) in the S_0 and S_1 States at $(S_0)_{min}$ and $(S_1)_{min}$ in Dichloromethane.

	Charge (S_0)	Charge (S_1)	μ (S_0)	μ (S_1)
**1a**				
at $(S_0)_{min}$	–	–	3.34	2.66
at $(S_1)_{min}$	–	–	3.24	2.72
**1b**				
at $(S_0)_{min}$	0.030	0.603	8.81	24.33
at $(S_1)_{min}$	0.040	0.564	8.95	23.07
**1g**				
at $(S_0)_{min}$	0.022	0.585	3.56	19.52
at $(S_1)_{min}$	0.034	0.561	4.27	19.06
**1e**				
at $(S_0)_{min}$	0.056	0.694	5.42	19.14
at $(S_1)_{min}$	0.067	0.668	5.51	18.38
**1f**				
at $(S_0)_{min}$	0.066	0.793	2.00	16.52
at $(S_1)_{min}$	0.083	0.746	1.39	15.71

Table SIII. Cartesian Coordinates (in Å) of **1a** at $(S_0)_{min}$ and $(S_1)_{min}$ in the Gas Phase.

	$(S_0)_{min}$			$(S_1)_{min}$		
	x	*y*	*z*	*x*	*y*	*z*
N1	−0.00057	−0.64502	0.00000	−0.00538	−0.64858	0.00000
N2	−1.24620	−1.14933	0.00000	−1.28575	−1.15342	0.00000
N3	0.01905	0.72879	0.00000	0.00719	0.73064	0.00000
C4	1.27943	−1.11948	0.00000	1.28843	−1.11649	0.00000
C5	2.11548	−0.00090	0.00000	2.12167	0.00157	0.00000
C6	1.32216	1.15036	0.00000	1.35056	1.16200	0.00000
C7	−1.30390	1.13512	0.00000	−1.30368	1.13330	0.00000
C8	−2.02987	−0.04554	0.00000	−2.04517	−0.06091	0.00000
H9	1.47271	−2.17846	0.00000	1.49519	−2.17415	0.00000
H10	3.19492	−0.02134	0.00000	3.20027	−0.03020	0.00000
H11	1.57204	2.19812	0.00000	1.60461	2.20875	0.00000
H12	−1.58662	2.17318	0.00000	−1.60244	2.16837	0.00000
H13	−3.10438	−0.15393	0.00000	−3.12128	−0.14930	0.00000

Table SIV. Cartesian Coordinates (in Å) of **1a** at $(S_0)_{min}$ and $(S_1)_{min}$ in Dichlomethane.

	$(S_0)_{min}$			$(S_1)_{min}$		
	x	*y*	*z*	*x*	*y*	*z*
N1	0.00159	−0.64855	0.00000	−0.00548	−0.64828	0.00000
N2	−1.24918	−1.15186	0.00000	−1.29159	−1.15696	0.00000
N3	0.01920	0.72440	0.00000	0.00263	0.73345	0.00000
C4	1.28176	−1.12165	0.00000	1.29450	−1.12038	0.00000
C5	2.11628	−0.00008	0.00000	2.12321	0.00234	0.00000
C6	1.32031	1.14930	0.00000	1.35477	1.16053	0.00000
C7	−1.30419	1.13403	0.00000	−1.30530	1.13044	0.00000
C8	−2.03257	−0.04201	0.00000	−2.05112	−0.05871	0.00000
H9	1.48087	−2.18005	0.00000	1.50714	−2.17722	0.00000
H10	3.19592	−0.01884	0.00000	3.20252	−0.02724	0.00000
H11	1.56519	2.19846	0.00000	1.61000	2.20728	0.00000
H12	−1.58309	2.17336	0.00000	−1.60873	2.16481	0.00000
H13	−3.10786	−0.14492	0.00000	−3.12830	−0.13849	0.00000

Table SV. Cartesian Coordinates (in Å) of **1b** at $(S_0)_{min}$ and $(S_1)_{min}$ in the Gas Phase.

	$(S_0)_{min}$			$(S_1)_{min}$		
	x	*y*	*z*	*x*	*y*	*z*
N1	2.75523	0.64651	0.00000	2.77631	0.63267	0.00000
N2	1.47816	1.04852	0.00000	1.51030	1.08209	0.00000
N3	2.88283	−0.72226	0.00000	2.85777	−0.70763	0.00000
C4	3.99282	1.21911	0.00000	4.01810	1.17539	0.00000
C5	4.91390	0.16844	0.00000	4.92598	0.11028	0.00000
C6	4.21410	−1.04126	0.00000	4.18917	−1.06431	0.00000
C7	1.59978	−1.23044	0.00000	1.58659	−1.18935	0.00000
C8	0.76690	−0.11256	0.00000	0.73305	−0.02497	0.00000
H9	4.10405	2.28984	0.00000	4.15091	2.24474	0.00000
H10	5.98824	0.27346	0.00000	6.00100	0.19013	0.00000
H11	4.54570	−2.06607	0.00000	4.48683	−2.09994	0.00000
H12	1.41271	−2.28956	0.00000	1.37536	−2.24409	0.00000
C13	−0.70215	−0.07755	0.00000	−0.68791	−0.00518	0.00000
C14	−1.37665	1.15658	0.00000	−1.41981	1.21928	0.00000
C15	−1.46013	−1.26161	0.00000	−1.42601	−1.23017	0.00000
C16	−2.84963	−1.22068	0.00000	−2.80023	−1.22280	0.00000
C17	−2.76590	1.20766	0.00000	−2.78882	1.21870	0.00000
C18	−3.51587	0.01786	0.00000	−3.51941	−0.00563	0.00000
H19	−0.96431	−2.22692	0.00000	−0.91191	−2.18610	0.00000
H20	−3.42490	−2.14041	0.00000	−3.34688	−2.15996	0.00000
H21	−0.79659	2.07229	0.00000	−0.88193	2.16063	0.00000
H22	−3.27772	2.16424	0.00000	−3.33278	2.15696	0.00000
C23	−4.94901	0.06504	0.00000	−4.93627	−0.00465	0.00000
N24	−6.11286	0.10225	0.00000	−6.10072	−0.00365	0.00000

Table SVI. Cartesian Coordinates (in Å) of **1b** at $(S_0)_{min}$ and $(S_1)_{min}$ in Dichloromethane.

	$(S_0)_{min}$			$(S_1)_{min}$		
	x	*y*	*z*	*x*	*y*	*z*
N1	2.76069	0.65279	0.00000	2.77528	0.64330	0.00000
N2	1.48213	1.06167	0.00000	1.49876	1.09331	0.00000
N3	2.87875	−0.71668	0.00000	2.85255	−0.70340	0.00000
C4	4.00181	1.21645	0.00000	4.01061	1.17729	0.00000
C5	4.91548	0.15731	0.00000	4.91770	0.10476	0.00000
C6	4.20666	−1.04668	0.00000	4.18317	−1.06503	0.00000
C7	1.59424	−1.22030	0.00000	1.59001	−1.18751	0.00000
C8	0.76627	−0.10129	0.00000	0.73561	−0.02157	0.00000
H9	4.12507	2.28628	0.00000	4.15608	2.24513	0.00000
H10	5.99077	0.25460	0.00000	5.99274	0.18434	0.00000
H11	4.52760	−2.07505	0.00000	4.47554	−2.10215	0.00000
H12	1.40537	−2.27933	0.00000	1.38537	−2.24461	0.00000
C13	−0.70255	−0.06863	0.00000	−0.68152	−0.01071	0.00000
C14	−1.38359	1.16289	0.00000	−1.41831	1.21735	0.00000
C15	−1.45453	−1.25806	0.00000	−1.42632	−1.23515	0.00000
C16	−2.84376	−1.22438	0.00000	−2.79753	−1.22887	0.00000
C17	−2.77297	1.20888	0.00000	−2.78598	1.21934	0.00000
C18	−3.51403	0.01297	0.00000	−3.52061	−0.00569	0.00000
H19	−0.95578	−2.22113	0.00000	−0.90914	−2.18861	0.00000
H20	−3.41164	−2.14834	0.00000	−3.34156	−2.16786	0.00000
H21	−0.81356	2.08464	0.00000	−0.87856	2.15780	0.00000
H22	−3.28720	2.16391	0.00000	−3.32630	2.16042	0.00000
C23	−4.94585	0.05351	0.00000	−4.93040	−0.00193	0.00000
N24	−6.11067	0.08644	0.00000	−6.09848	0.00252	0.00000

Table SVII. Cartesian Coordinates (in Å) of **1g** at $(S_0)_{min}$ and $(S_1)_{min}$ in the Gas Phase.

	$(S_0)_{min}$			$(S_1)_{min}$		
	x	*y*	*z*	*x*	*y*	*z*
N1	2.97119	2.15281	0.00000	2.97507	2.18381	0.00000
N2	2.34703	0.96824	0.00000	2.40238	0.96675	0.00000
N3	2.09873	3.21400	0.00000	2.07913	3.18568	0.00000
C4	4.25392	2.61740	0.00000	4.23286	2.68369	0.00000
C5	4.16838	4.01182	0.00000	4.11503	4.07938	0.00000
C6	2.81961	4.37814	0.00000	2.76299	4.38317	0.00000
C7	0.82986	2.67190	0.00000	0.84371	2.62212	0.00000
C8	1.02394	1.29120	0.00000	1.07039	1.19454	0.00000
H9	5.08652	1.93515	0.00000	5.08626	2.02590	0.00000
H10	5.00532	4.69369	0.00000	4.92865	4.78654	0.00000
H11	2.33338	5.33921	0.00000	2.23660	5.32331	0.00000
H12	−0.04868	3.29205	0.00000	−0.05531	3.21274	0.00000
C13	0.00139	0.23500	0.00000	0.07886	0.17595	0.00000
C14	0.38286	−1.11798	0.00000	0.42438	−1.20670	0.00000
C15	−1.36951	0.54914	0.00000	−1.30762	0.52274	0.00000
C16	−2.32918	−0.45645	0.00000	−2.27393	−0.45398	0.00000
C17	−0.57674	−2.12624	0.00000	−0.54833	−2.17229	0.00000
C18	−1.94288	−1.80514	0.00000	−1.92806	−1.82276	0.00000
H19	−1.69163	1.58579	0.00000	−1.61683	1.56369	0.00000
H20	−3.38684	−0.21560	0.00000	−3.32642	−0.19218	0.00000
H21	1.43814	−1.36660	0.00000	1.47058	−1.49256	0.00000
H22	−0.27062	−3.16605	0.00000	−0.27273	−3.22004	0.00000
C23	−3.01177	−2.84059	0.00000	−2.99460	−2.80675	0.00000
O24	−2.51778	−4.10139	0.00000	−2.53730	−4.09254	0.00000
O25	−4.20647	−2.60034	0.00000	−4.19506	−2.56184	0.00000
C26	−3.49630	−5.15610	0.00000	−3.54501	−5.09670	0.00000
H27	−4.12670	−5.09183	0.89025	−4.17774	−5.01303	0.88700
H28	−4.12670	−5.09183	−0.89025	−4.17774	−5.01303	−0.88700
H29	−2.92260	−6.08264	0.00000	−3.01432	−6.04886	0.00000

Table SVIII. Cartesian Coordinates (in Å) of **1g** at $(S_0)_{min}$ and $(S_1)_{min}$ in Dichloromethane.

	$(S_0)_{min}$			$(S_1)_{min}$		
	x	*y*	*z*	*x*	*y*	*z*
N1	2.97915	2.15213	0.00000	2.98337	2.17298	0.00000
N2	2.35552	0.96292	0.00000	2.39958	0.95020	0.00000
N3	2.10187	3.20941	0.00000	2.08278	3.17926	0.00000
C4	4.25911	2.62195	0.00000	4.23202	2.67159	0.00000
C5	4.16609	4.01752	0.00000	4.11182	4.07191	0.00000
C6	2.81577	4.37685	0.00000	2.76558	4.37775	0.00000
C7	0.83316	2.66597	0.00000	0.85013	2.62620	0.00000
C8	1.02871	1.28750	0.00000	1.07241	1.19816	0.00000
H9	5.09790	1.94659	0.00000	5.09184	2.02182	0.00000
H10	5.00001	4.70334	0.00000	4.92755	4.77665	0.00000
H11	2.32236	5.33443	0.00000	2.23506	5.31580	0.00000
H12	−0.04514	3.28723	0.00000	−0.04384	3.22578	0.00000
C13	0.00442	0.23315	0.00000	0.07363	0.19136	0.00000
C14	0.37892	−1.12262	0.00000	0.41434	−1.19797	0.00000
C15	−1.36616	0.55287	0.00000	−1.31677	0.53449	0.00000
C16	−2.32924	−0.44972	0.00000	−2.28210	−0.44040	0.00000
C17	−0.58435	−2.12757	0.00000	−0.55755	−2.16249	0.00000
C18	−1.95020	−1.80148	0.00000	−1.94177	−1.81740	0.00000
H19	−1.68464	1.58996	0.00000	−1.62339	1.57543	0.00000
H20	−3.38369	−0.19604	0.00000	−3.33136	−0.16555	0.00000
H21	1.43152	−1.38189	0.00000	1.46023	−1.48493	0.00000
H22	−0.27792	−3.16712	0.00000	−0.27556	−3.20891	0.00000
C23	−3.01780	−2.83704	0.00000	−2.99620	−2.80301	0.00000
O24	−2.52833	−4.09265	0.00000	−2.53936	−4.08662	0.00000
O25	−4.21663	−2.59274	0.00000	−4.20707	−2.56498	0.00000
C26	−3.49697	−5.16249	0.00000	−3.53388	−5.10944	0.00000
H27	−4.12327	−5.10667	0.89287	−4.16390	−5.03948	0.88940
H28	−4.12327	−5.10667	−0.89287	−4.16390	−5.03948	−0.88940
H29	−2.91103	−6.08035	0.00000	−2.98780	−6.05196	0.00000

Table SIX. Cartesian Coordinates (in Å) of **1e** at $(S_0)_{min}$ and $(S_1)_{min}$ in the Gas Phase.

	$(S_0)_{min}$			$(S_1)_{min}$		
	x	*y*	*z*	*x*	*y*	*z*
N1	0.58409	3.63624	0.00000	0.55370	3.65758	0.00000
N2	1.01971	2.37408	0.00000	1.03791	2.41129	0.00000
N3	−0.78754	3.72379	0.00000	−0.78718	3.69241	0.00000
C4	1.11989	4.88983	0.00000	1.05222	4.92187	0.00000
C5	0.04081	5.77925	0.00000	−0.04233	5.78668	0.00000
C6	−1.14696	5.04558	0.00000	−1.19032	5.00683	0.00000
C7	−1.26392	2.43135	0.00000	−1.22294	2.40253	0.00000
C8	−0.12492	1.62572	0.00000	−0.04151	1.60006	0.00000
H9	2.18660	5.03409	0.00000	2.11620	5.09126	0.00000
H10	0.11436	6.85629	0.00000	−0.00321	6.86408	0.00000
H11	−2.18160	5.34484	0.00000	−2.23675	5.26433	0.00000
H12	−2.31717	2.21947	0.00000	−2.26842	2.14428	0.00000
C13	0.00106	0.15998	0.00000	0.08710	0.16581	0.00000
C14	1.29339	−0.39728	0.00000	1.37492	−0.42207	0.00000
C15	−1.09740	−0.74078	0.00000	−1.05603	−0.72554	0.00000
C16	−0.88606	−2.12790	0.00000	−0.84238	−2.10533	0.00000
C17	1.49856	−1.77113	0.00000	1.56079	−1.77638	0.00000
C18	0.40631	−2.65239	0.00000	0.43058	−2.65229	0.00000
H19	−1.73252	−2.80555	0.00000	−1.69173	−2.77991	0.00000
H20	2.14003	0.27829	0.00000	2.23937	0.23269	0.00000
H21	2.50732	−2.16746	0.00000	2.55834	−2.19593	0.00000
C22	0.55994	−4.13545	0.00000	0.54564	−4.11037	0.00000
O23	1.85495	−4.51944	0.00000	1.83698	−4.52886	0.00000
O24	−0.36980	−4.92089	0.00000	−0.38368	−4.89647	0.00000
C25	2.08637	−5.94195	0.00000	2.00569	−5.94018	0.00000
H26	1.64759	−6.39742	0.89073	1.54868	−6.38701	0.88639
H27	1.64759	−6.39742	−0.89073	1.54868	−6.38701	−0.88639
H28	3.16965	−6.05748	0.00000	3.08232	−6.11220	0.00000
C29	−2.46078	−0.29680	0.00000	−2.39475	−0.26543	0.00000
N30	−3.57618	0.03670	0.00000	−3.48452	0.14942	0.00000

Table SX. Cartesian Coordinates (in Å) of **1e** at $(S_0)_{min}$ and $(S_1)_{min}$ in Dichloromethane.

	$(S_0)_{min}$			$(S_1)_{min}$		
	x	*y*	*z*	*x*	*y*	*z*
N1	0.58923	3.64112	0.00000	0.57044	3.65901	0.00000
N2	1.02534	2.37528	0.00000	1.04783	2.39973	0.00000
N3	−0.78244	3.72744	0.00000	−0.77484	3.70035	0.00000
C4	1.12386	4.89404	0.00000	1.07219	4.91120	0.00000
C5	0.04274	5.78281	0.00000	−0.02366	5.78555	0.00000
C6	−1.14399	5.04759	0.00000	−1.17351	5.01800	0.00000
C7	−1.25923	2.43447	0.00000	−1.22353	2.42127	0.00000
C8	−0.12267	1.62771	0.00000	−0.04321	1.60437	0.00000
H9	2.19041	5.04268	0.00000	2.13590	5.08498	0.00000
H10	0.11541	6.86003	0.00000	0.02544	6.86233	0.00000
H11	−2.17962	5.34411	0.00000	−2.21880	5.28038	0.00000
H12	−2.31357	2.23001	0.00000	−2.27312	2.18543	0.00000
C13	−0.00060	0.16211	0.00000	0.06516	0.17869	0.00000
C14	1.28943	−0.40098	0.00000	1.35718	−0.42028	0.00000
C15	−1.09981	−0.73869	0.00000	−1.07325	−0.72109	0.00000
C16	−0.89221	−2.12683	0.00000	−0.86199	−2.10284	0.00000
C17	1.49192	−1.77524	0.00000	1.54077	−1.77418	0.00000
C18	0.39824	−2.65526	0.00000	0.41116	−2.65588	0.00000
H19	−1.74280	−2.79854	0.00000	−1.71788	−2.76895	0.00000
H20	2.14152	0.26708	0.00000	2.22262	0.23220	0.00000
H21	2.50139	−2.16918	0.00000	2.54119	−2.18768	0.00000
C22	0.55374	−4.13814	0.00000	0.53673	−4.10978	0.00000
O23	1.84182	−4.52009	0.00000	1.82220	−4.52502	0.00000
O24	−0.38178	−4.92359	0.00000	−0.39640	−4.90571	0.00000
C25	2.09344	−5.94327	0.00000	2.03187	−5.93993	0.00000
H26	1.66421	−6.40143	0.89318	1.59190	−6.39299	0.89033
H27	1.66421	−6.40143	−0.89318	1.59190	−6.39299	−0.89033
H28	3.17758	−6.04234	0.00000	3.11192	−6.07635	0.00000
C29	−2.46497	−0.30282	0.00000	−2.42252	−0.28909	0.00000
N30	−3.58739	0.00747	0.00000	−3.54028	0.04541	0.00000

Table SXI. Cartesian Coordinates (in Å) of **1f** at $(S_0)_{min}$ and $(S_1)_{min}$ in the Gas Phase.

	$(S_0)_{min}$			$(S_1)_{min}$		
	x	*y*	*z*	*x*	*y*	*z*
N1	−3.44266	0.73344	0.20872	−3.45529	0.75270	0.12262
N2	−2.33662	−0.00988	0.31504	−2.37410	−0.02530	0.18583
N3	−3.17720	2.03723	−0.14794	−3.15211	2.04982	−0.05050
C4	−4.78609	0.56917	0.36509	−4.80391	0.59514	0.20214
C5	−5.36862	1.81003	0.09288	−5.36299	1.86430	0.07362
C6	−4.36014	2.72184	−0.22626	−4.32054	2.76902	−0.08066
C7	−1.80967	2.13409	−0.28116	−1.79399	2.12524	−0.16369
C8	−1.33396	0.85746	0.00775	−1.32449	0.79760	−0.00448
H9	−5.19511	−0.38830	0.63862	−5.23232	−0.38408	0.33983
H10	−6.42557	2.02765	0.12246	−6.41426	2.10238	0.09616
H11	−4.38726	3.76570	−0.49026	−4.31000	3.83966	−0.20394
H12	−1.32497	3.05380	−0.55561	−1.28068	3.05982	−0.30863
C13	0.05449	0.37855	−0.00458	0.03889	0.31672	−0.00459
C14	0.37682	−0.90628	−0.50781	0.38692	−0.99505	−0.47572
C15	1.12195	1.16571	0.49653	1.12987	1.15268	0.44622
C16	2.43878	0.68766	0.50226	2.42930	0.67864	0.44859
C17	1.69695	−1.38049	−0.48633	1.69130	−1.43716	−0.46445
C18	2.73002	−0.58878	0.01937	2.73961	−0.61162	0.00679
H19	3.24149	1.30195	0.89465	3.23798	1.30924	0.80070
H20	1.91267	−2.36612	−0.88022	1.92133	−2.42804	−0.83442
C21	4.15155	−1.05011	0.06028	4.13613	−1.03653	0.03902
O22	4.29854	−2.29679	−0.42616	4.30587	−2.30242	−0.41442
O23	5.06925	−0.37497	0.48480	5.06780	−0.35466	0.42453
C24	5.64340	−2.82169	−0.42329	5.65070	−2.76686	−0.40454
H25	6.29444	−2.19311	−1.03465	6.28235	−2.13951	−1.03791
H26	6.03036	−2.86030	0.59724	6.05689	−2.75690	0.60963
H27	5.56126	−3.82216	−0.84559	5.61625	−3.78537	−0.79100
C28	0.89817	2.47753	1.03841	0.89482	2.47043	0.92585
N29	0.77768	3.54846	1.47675	0.64766	3.54982	1.28122
C30	−0.60076	−1.76408	−1.12232	−0.59037	−1.87863	−1.03798
N31	−1.28926	−2.51930	−1.67752	−1.35869	−2.60321	−1.51464

Table SXII. Cartesian Coordinates (in Å) of **1f** at $(S_0)_{min}$ and $(S_1)_{min}$ in Dichloromethane.

	$(S_0)_{min}$			$(S_1)_{min}$		
	x	*y*	*z*	*x*	*y*	*z*
N1	−3.44763	0.75203	0.26158	−3.46944	0.75196	0.12477
N2	−2.34185	0.00579	0.41168	−2.38147	−0.03204	0.21533
N3	−3.17453	2.01826	−0.20446	−3.15536	2.04807	−0.06625
C4	−4.78952	0.61649	0.44942	−4.81128	0.60287	0.17689
C5	−5.36355	1.83823	0.08218	−5.36392	1.87714	0.00810
C6	−4.34993	2.70781	−0.32518	−4.31994	2.77315	−0.14203
C7	−1.80687	2.09229	−0.36331	−1.80154	2.12694	−0.15252
C8	−1.33809	0.84194	0.02020	−1.33378	0.79382	0.03423
H9	−5.20843	−0.30735	0.81102	−5.25787	−0.36651	0.32796
H10	−6.41779	2.06961	0.10818	−6.41499	2.11594	0.00156
H11	−4.36721	3.72606	−0.67640	−4.30023	3.84049	−0.29084
H12	−1.32186	2.97372	−0.74412	−1.29536	3.05466	−0.35571
C13	0.05137	0.36352	0.01656	0.02189	0.32306	0.03841
C14	0.38601	−0.90812	−0.50716	0.37945	−0.99974	−0.41606
C15	1.10885	1.14303	0.54436	1.12086	1.15685	0.47739
C16	2.42993	0.67546	0.54328	2.42506	0.69705	0.45824
C17	1.70803	−1.37665	−0.49376	1.69024	−1.43041	−0.42417
C18	2.73285	−0.58611	0.03034	2.74201	−0.59445	0.01193
H19	3.22378	1.28694	0.95622	3.22573	1.33914	0.80728
H20	1.93237	−2.35264	−0.90583	1.91801	−2.42517	−0.78606
C21	4.15753	−1.04180	0.06200	4.13726	−1.01890	0.01283
O22	4.31062	−2.27072	−0.44752	4.30565	−2.28094	−0.43211
O23	5.07094	−0.36520	0.50342	5.08090	−0.32560	0.37319
C24	5.65651	−2.80381	−0.46245	5.65097	−2.76940	−0.46213
H25	6.30471	−2.16527	−1.06534	6.26699	−2.15306	−1.11924
H26	6.04324	−2.87036	0.55606	6.08015	−2.76929	0.54134
H27	5.56613	−3.79299	−0.90712	5.58457	−3.78548	−0.84652
C28	0.86672	2.42858	1.13857	0.90329	2.45649	1.01692
N29	0.73723	3.47116	1.63857	0.72331	3.51879	1.45462
C30	−0.59452	−1.75334	−1.13253	−0.58156	−1.91167	−0.95475
N31	−1.30510	−2.48866	−1.68728	−1.30967	−2.68586	−1.42141

\\Bot025\data\Šw¶\‘ààV\NMR-TAPSu-trans\1043-2-1.als
5-1H

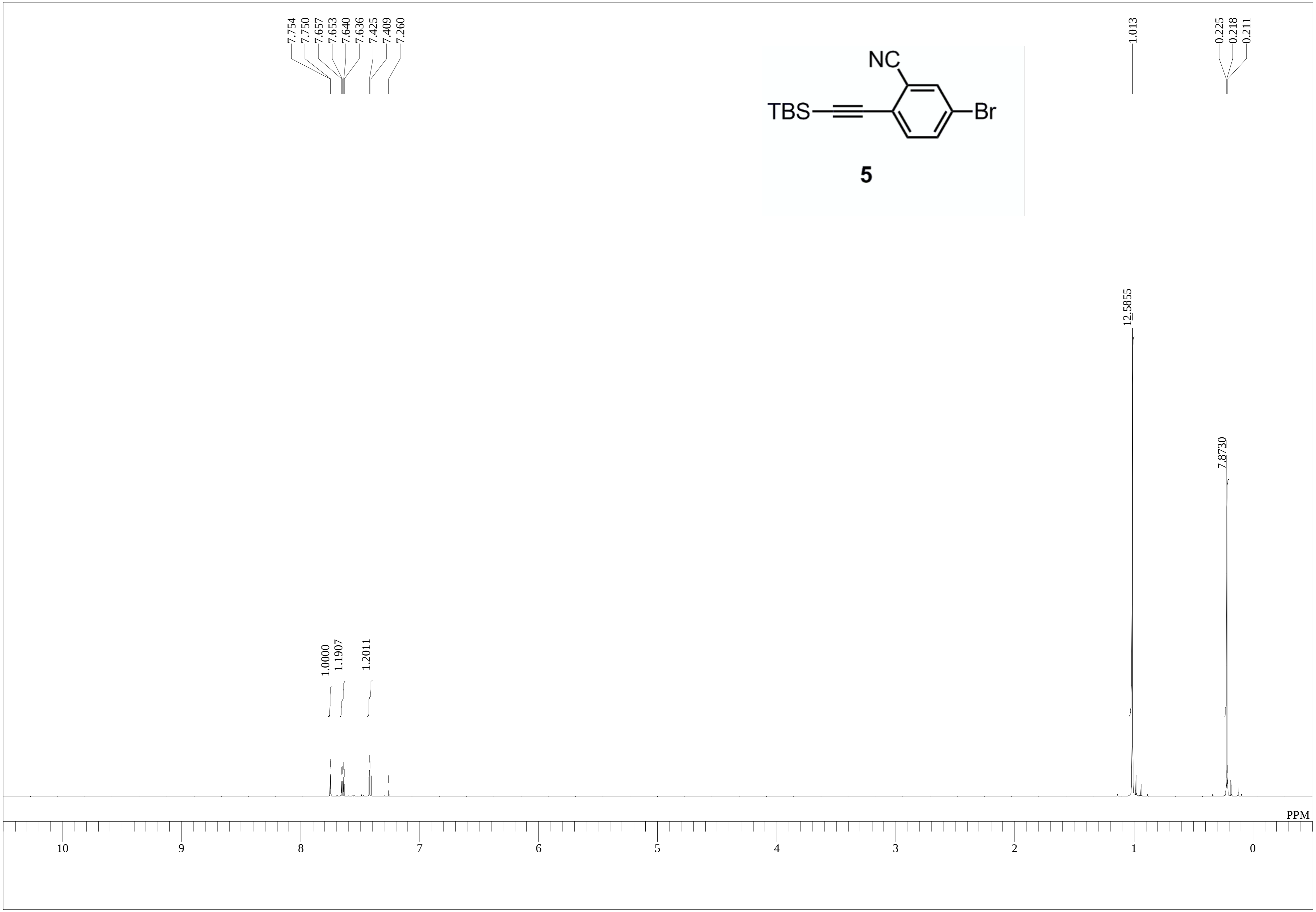

\\Bot025\data\Šw¶\‘ååV\NMR-TAPSu-trans\1043-13C-1.als
5-13C

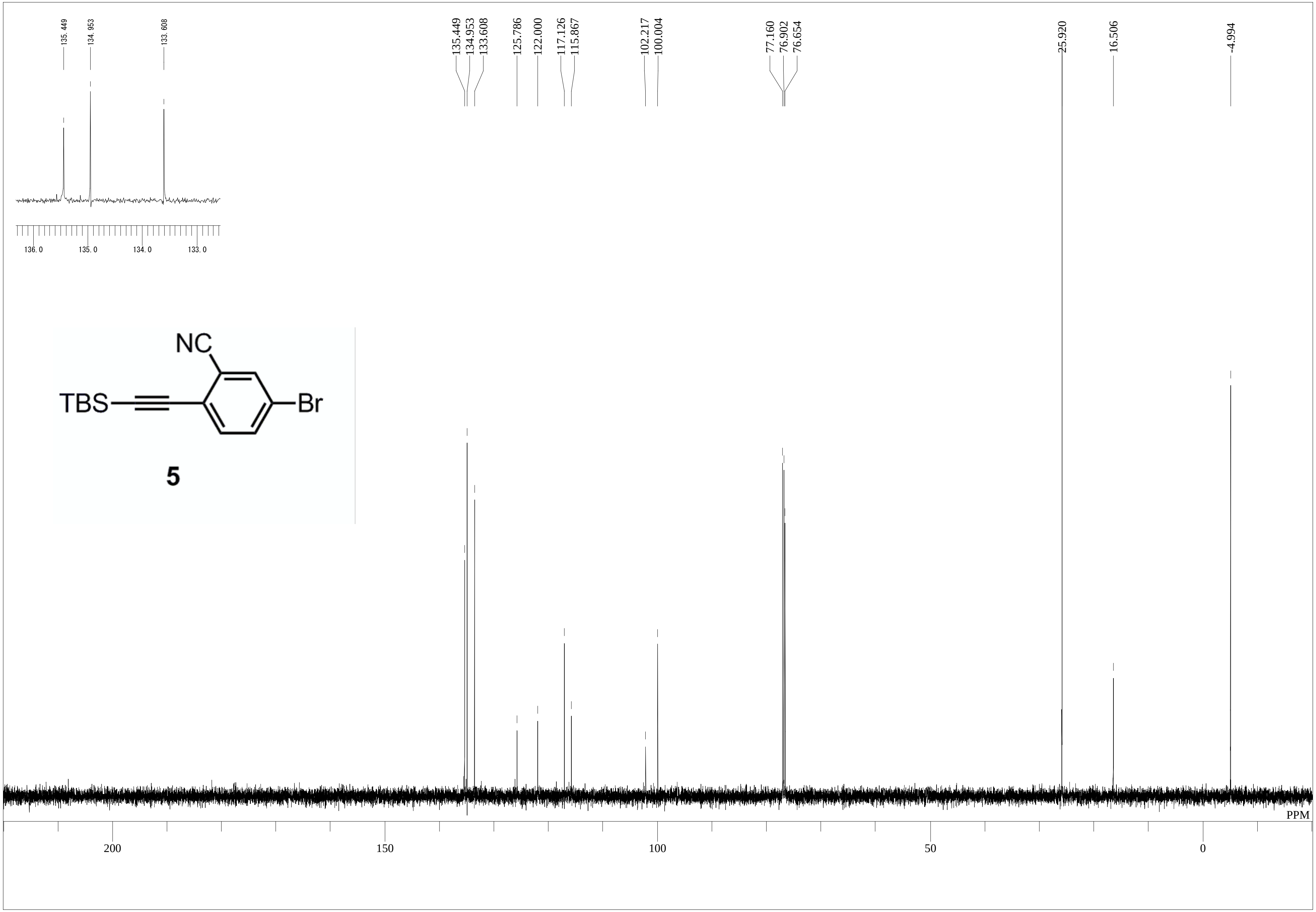

\\Bot025\data\Šwľ¶\'ååV\NMR-TAPSu-trans\'Ç‰Á\1232-cc-1.als
6-1H

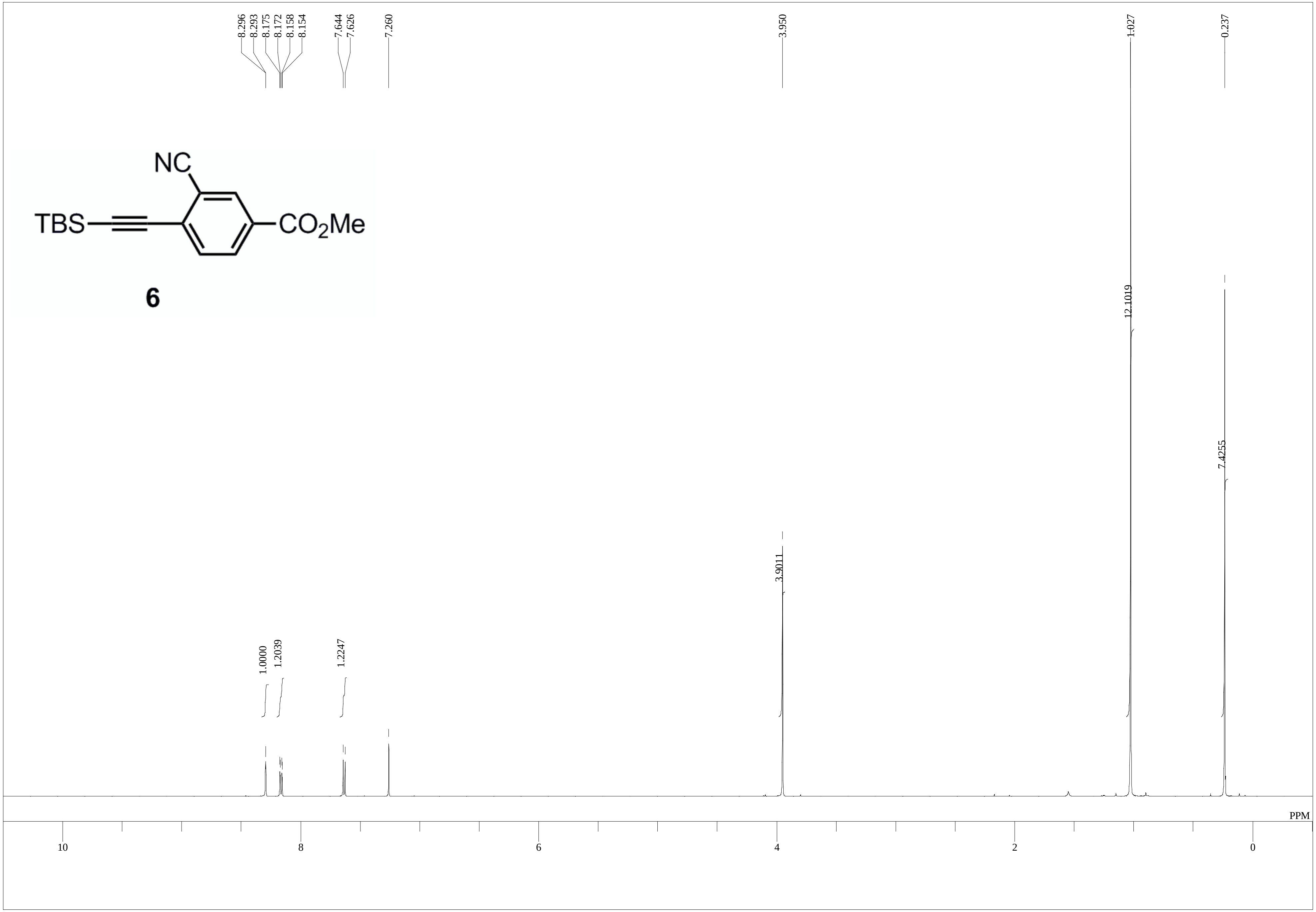

\\Bot025\data\Šw¶\‘ååV\NMR-TAPSu-trans\1232-13C-1.als
6-13C

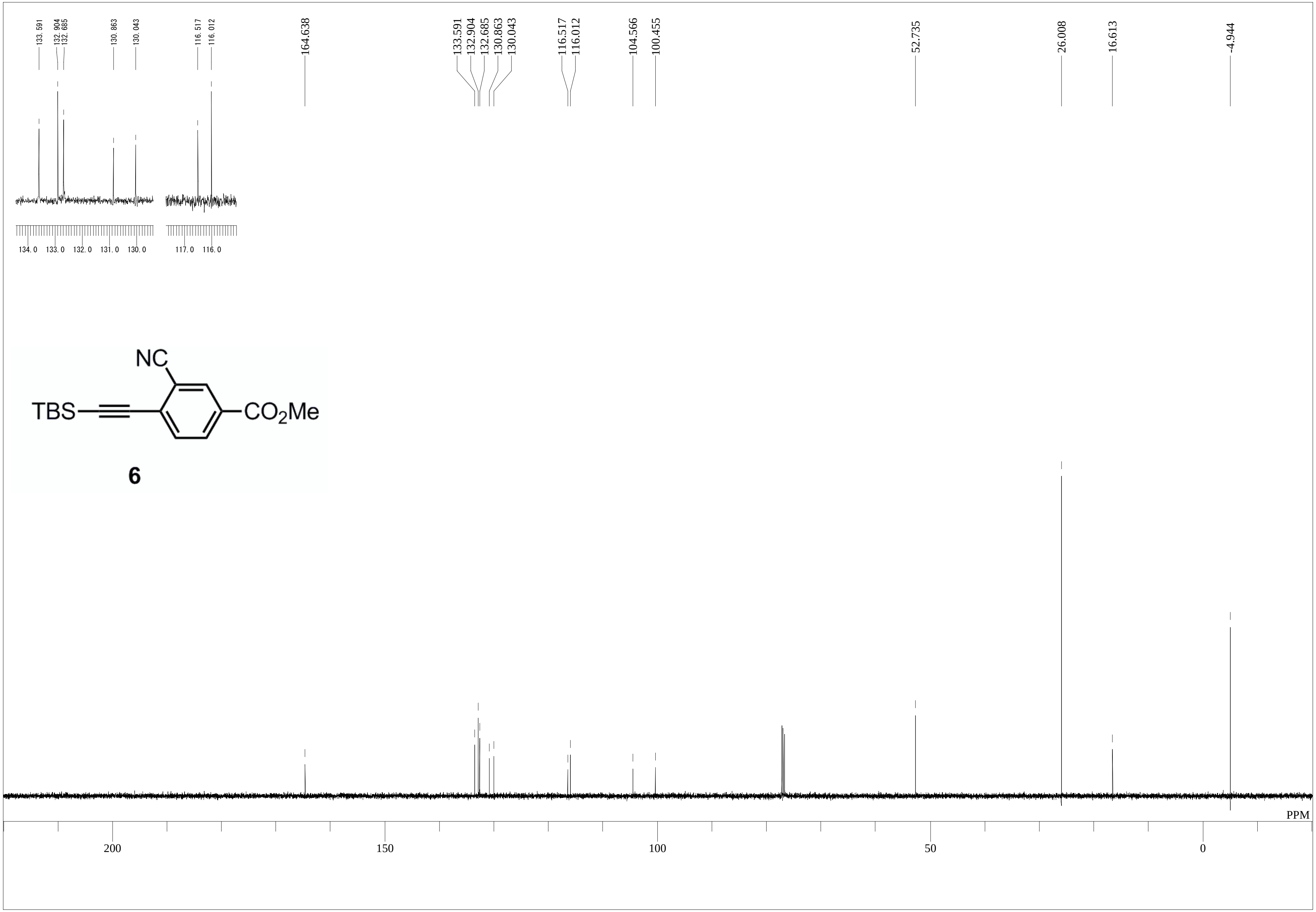

\\Bot025\data\Šw¶\‘ååV\NMR-TAPSu-trans\alkyne.als
3b-1H

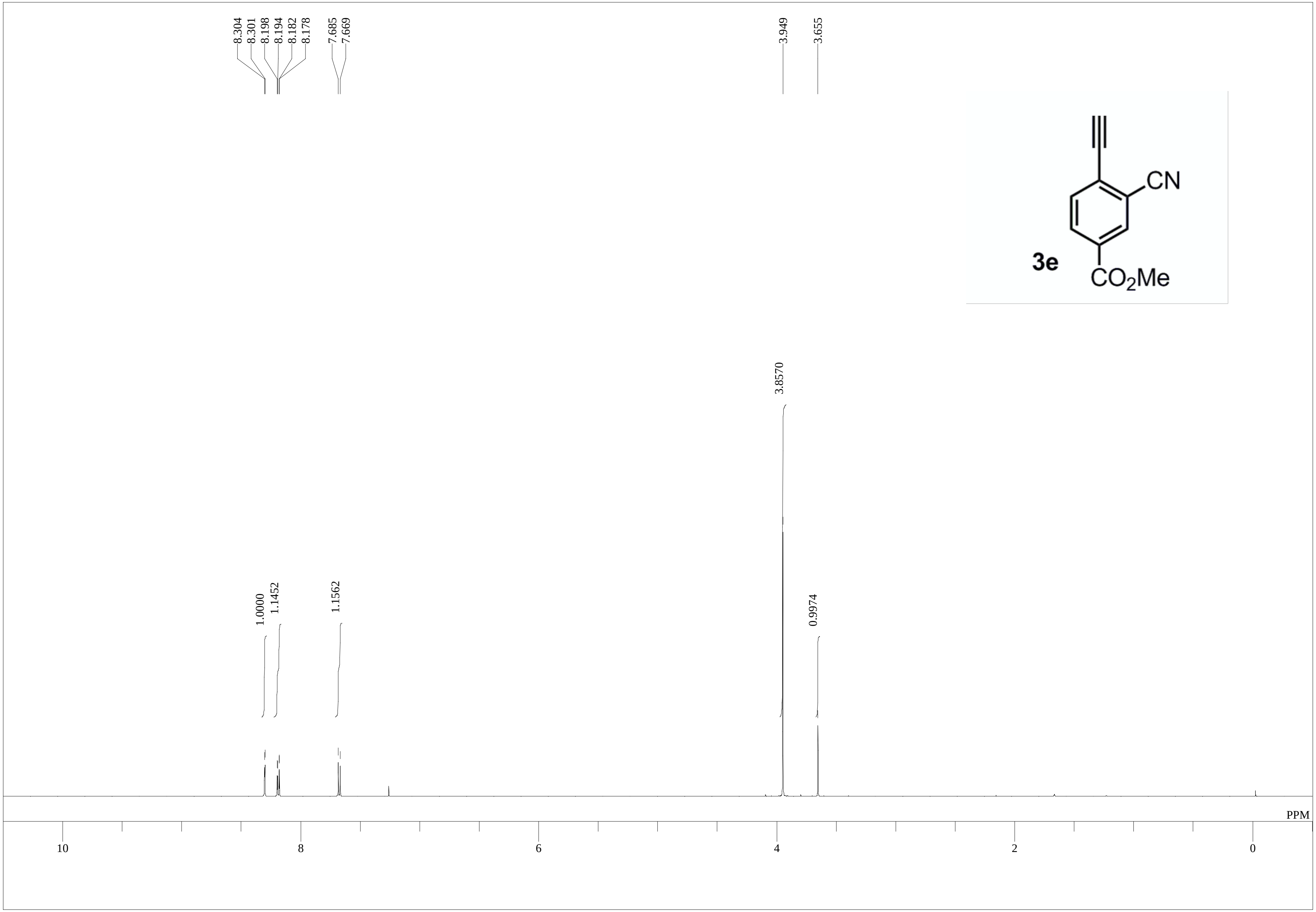

\\Bot025\data\Šw¶\‘ååV\NMR-TAPSu-trans\alkyne-13C-1.als
3b-13C

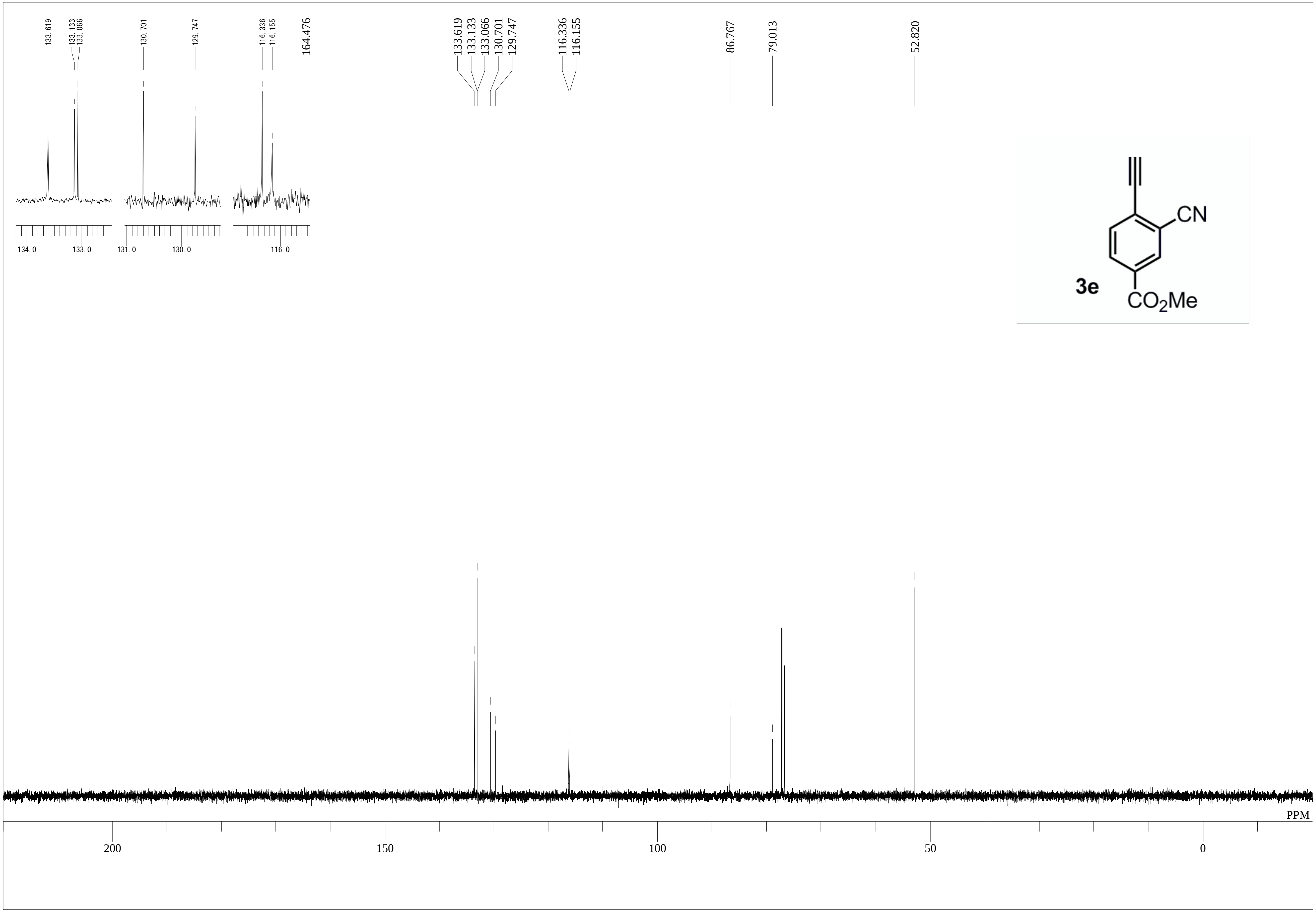

\\Bot025\data\Šw¶\‘åàV\NMR-TAPSu-trans\1225-1.als
3c-1H

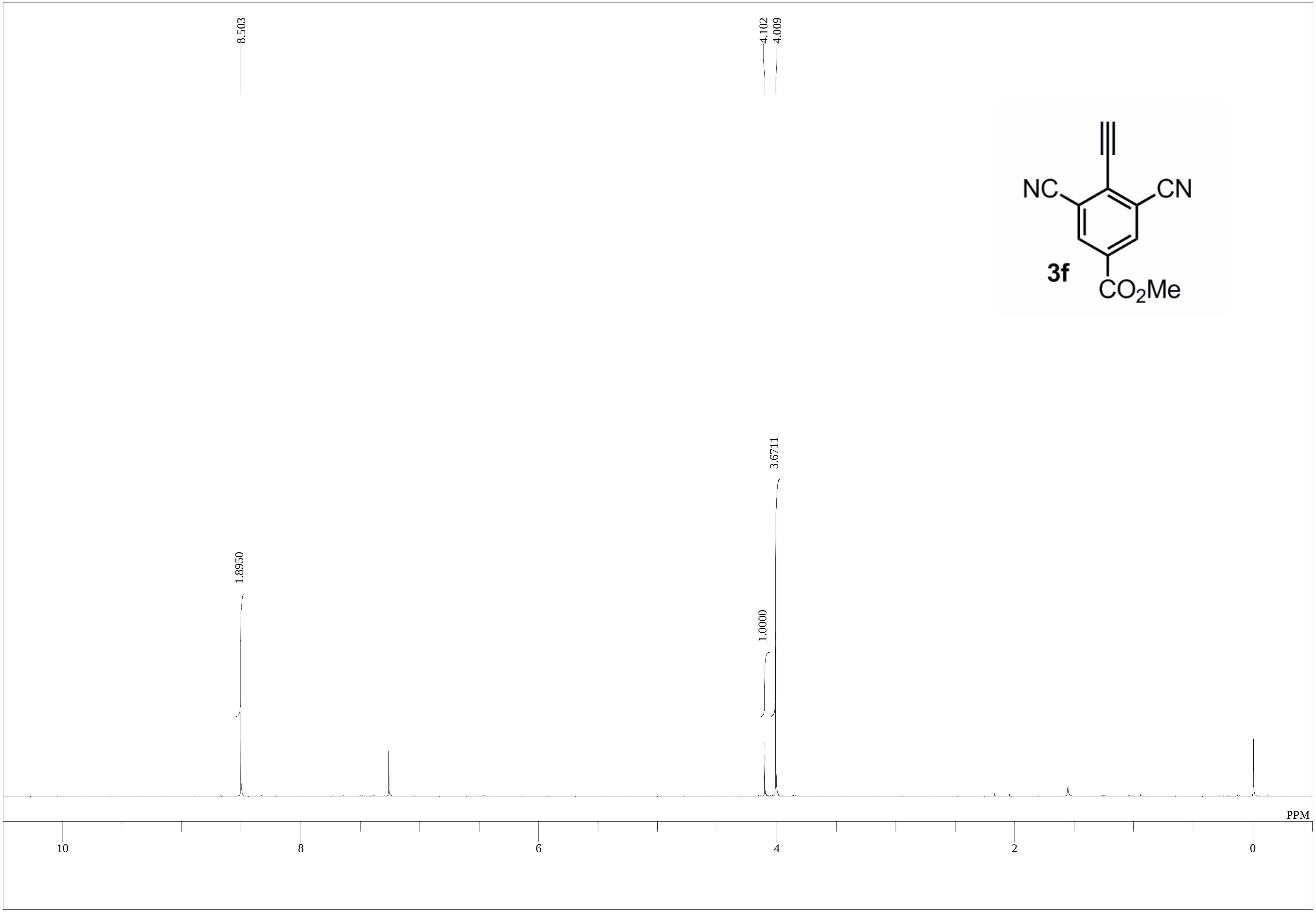

\\Bot025\data\Šw▯¶\'åàV\NMR-TAPSu-trans\1225-13C_copy-1.als
3c-13C

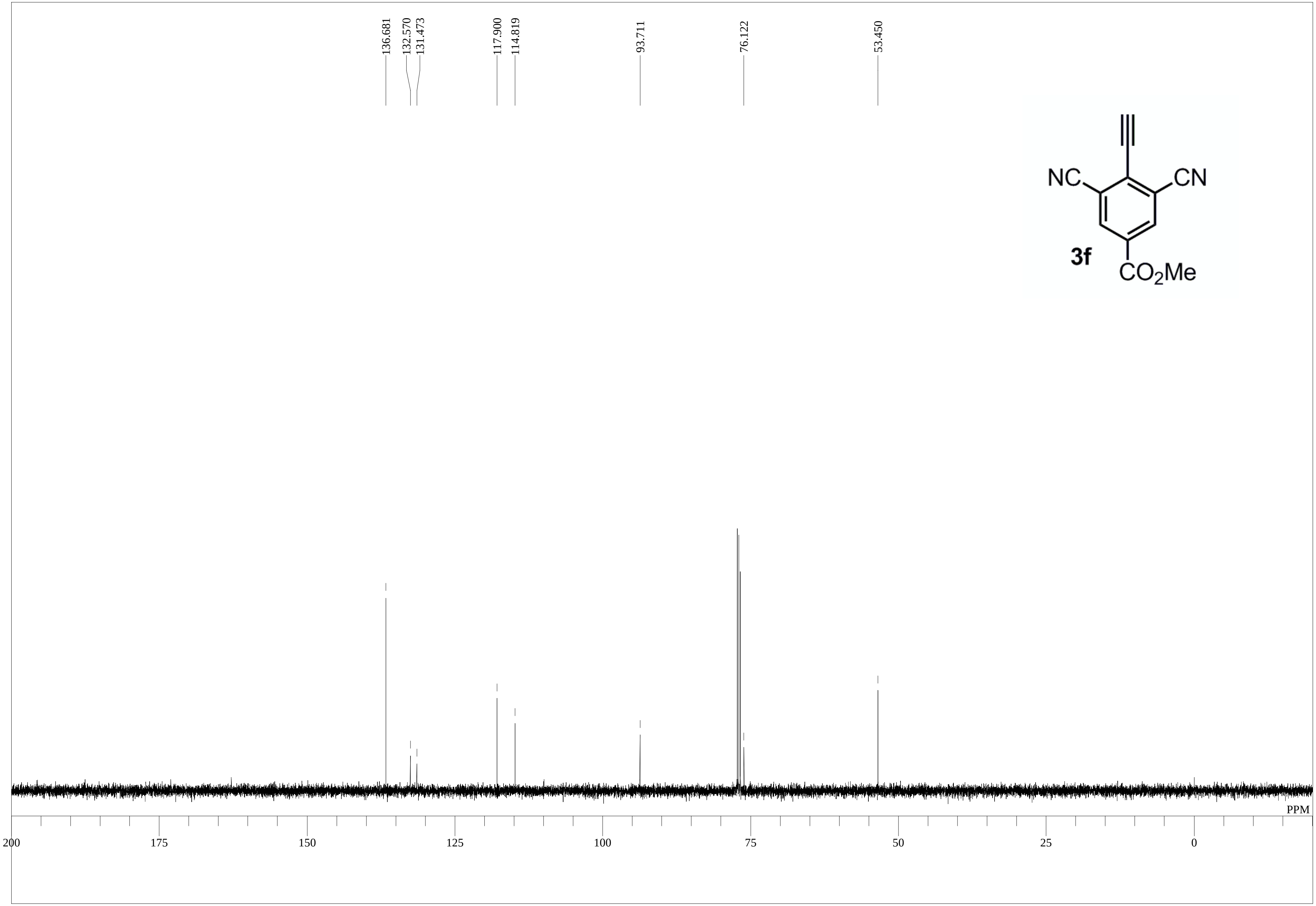

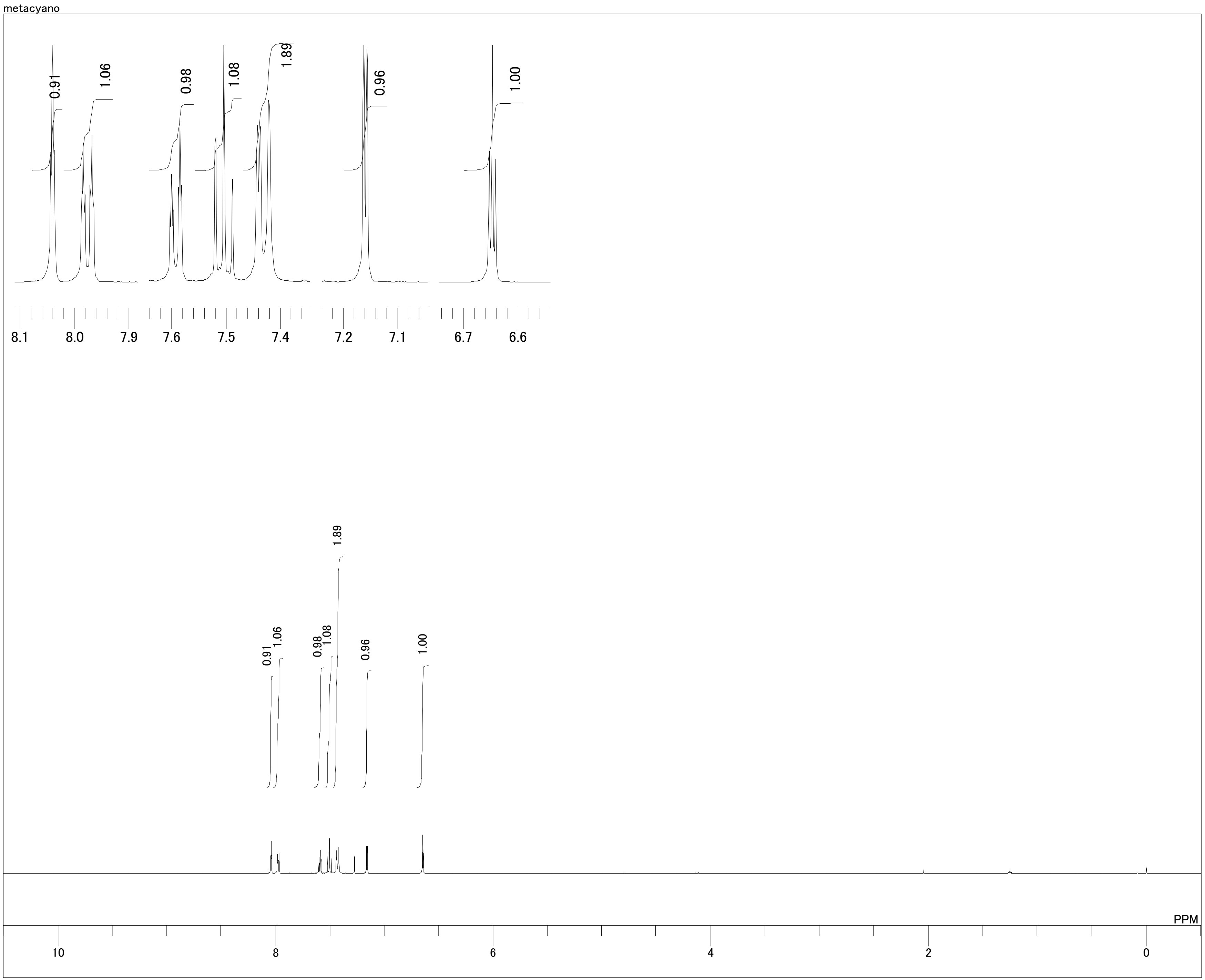

DFILE	C:¥Documents and Settings¥All Users¥Doc
COMNT	metacyano
DATIM	07-12-2012 16:46:16
OBNUC	1H
EXMOD	single_pulse.ex2
OBFRQ	500.16 MHz
OBSET	2.41 KHz
OBFIN	6.01 Hz
POINT	16384
FREQU	9384.38 Hz
SCANS	7
ACQTM	1.7459 sec
PD	1.0000 sec
PW1	6.50 usec
IRNUC	1H
CTEMP	19.9 c
SLVNT	CDCL3
EXREF	0.00 ppm
BF	0.12 Hz
RGAIN	44

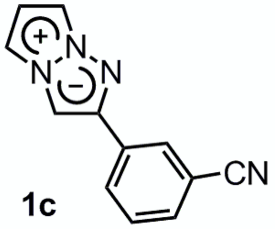

1c

metacyano-13C

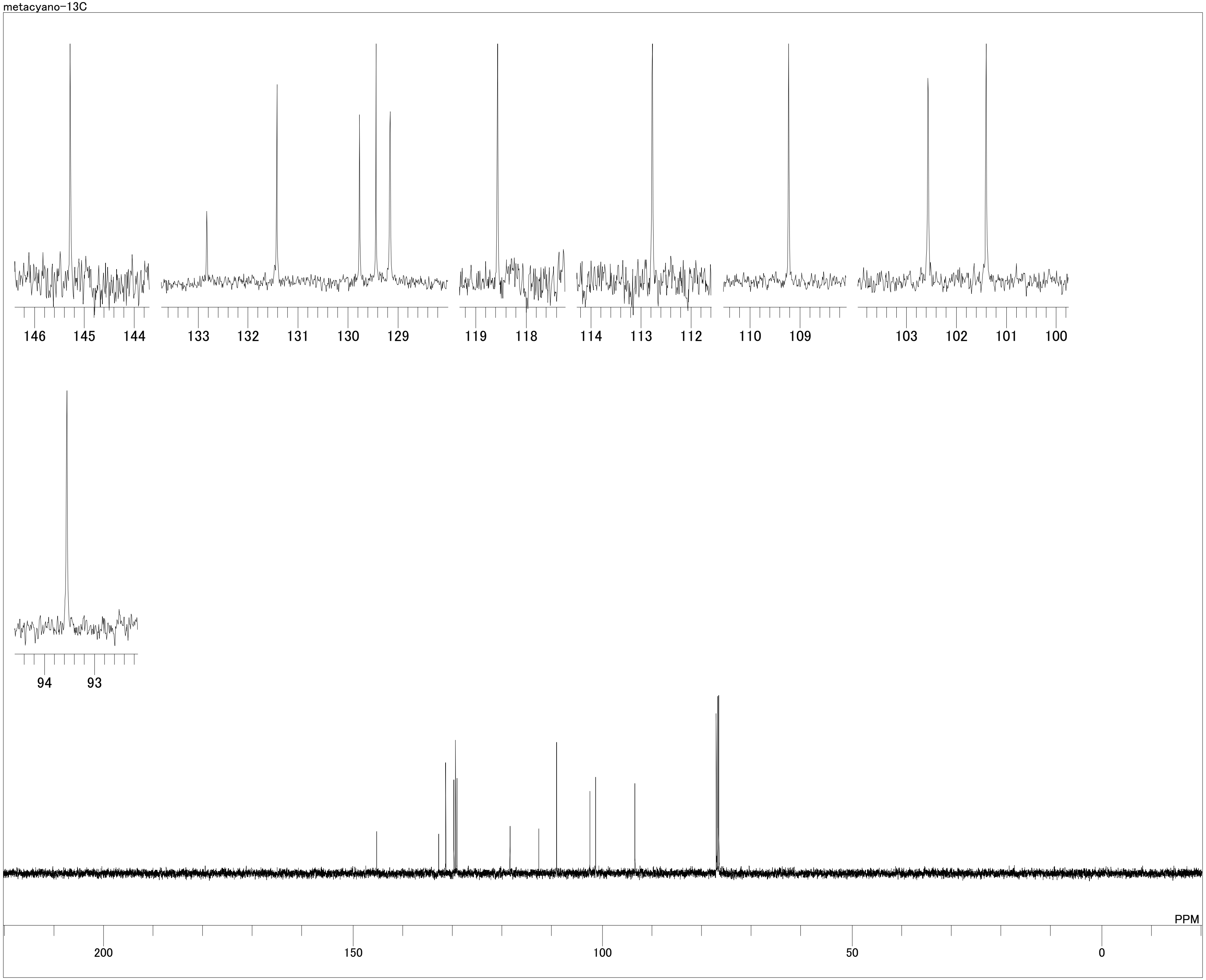

DFILE	C:¥Documents and Settings¥All Users¥Doc
COMNT	metacyano-13C
DATIM	07-12-2012 16:49:01
OBNUC	13C
EXMOD	single_pulse_dec
OBFRQ	125.77 MHz
OBSET	7.87 KHz
OBFIN	4.21 Hz
POINT	32768
FREQU	39308.18 Hz
SCANS	32
ACQTM	0.8336 sec
PD	2.0000 sec
PW1	3.83 usec
IRNUC	1H
CTEMP	20.4 c
SLVNT	CDCL3
EXREF	77.00 ppm
BF	1.20 Hz
RGAIN	60

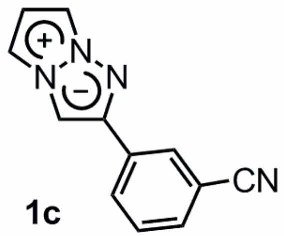

1c

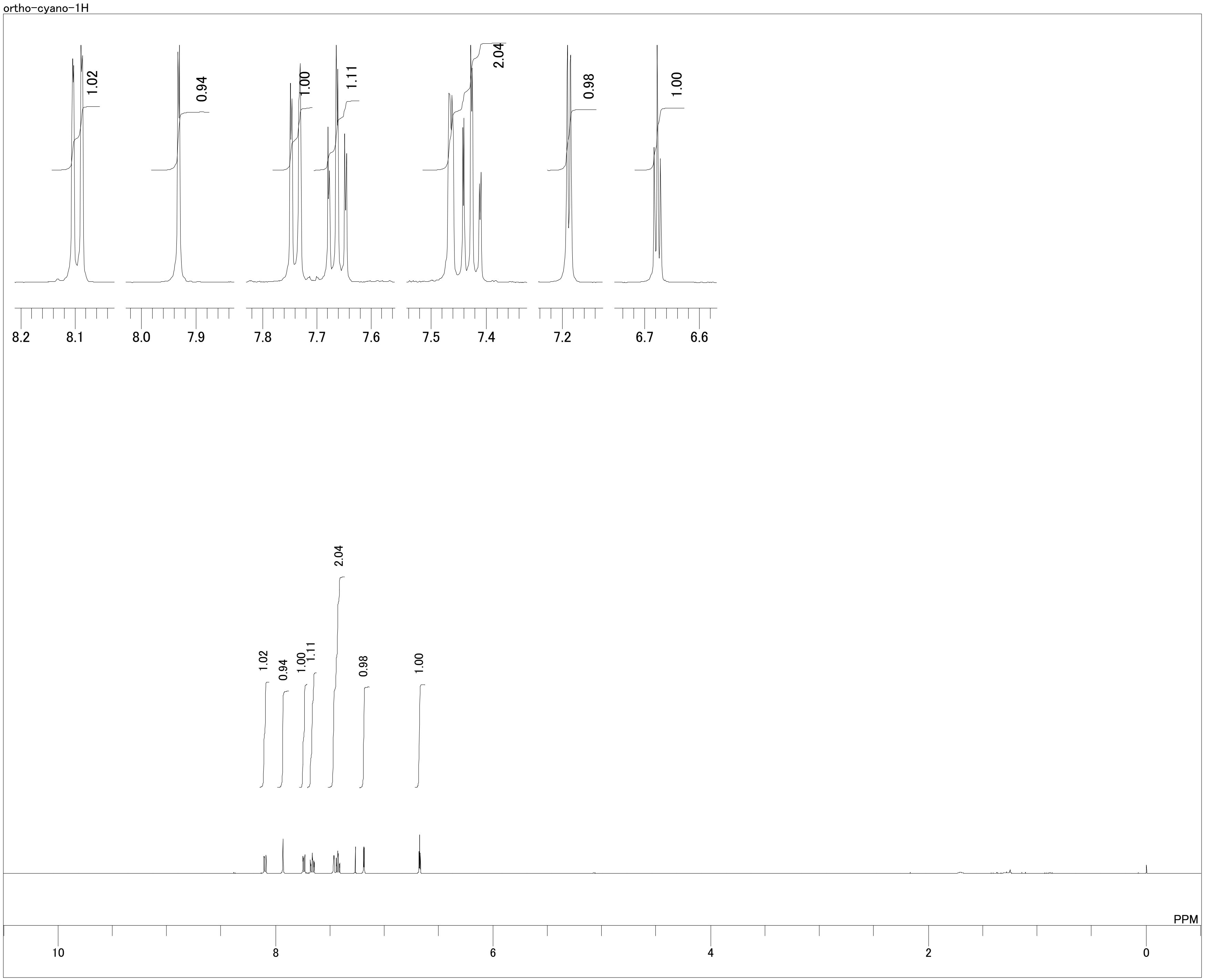

DFILE C:¥Documents and Settings¥All Users¥Doc
COMNT ortho-cyano-1H
DATIM 14-12-2012 11:25:28
OBNUC 1H
EXMOD single_pulse.ex2
OBFRQ 500.16 MHz
OBSET 2.41 KHz
OBFIN 6.01 Hz
POINT 16384
FREQU 9384.38 Hz
SCANS 8
ACQTM 1.7459 sec
PD 1.0000 sec
PW1 6.50 usec
IRNUC 1H
CTEMP 20.5 c
SLVNT CDCL3
EXREF 0.00 ppm
BF 0.12 Hz
RGAIN 48

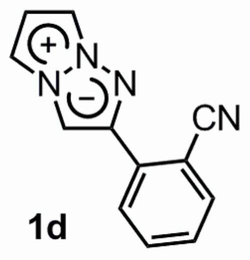

ortho-cyano-13C

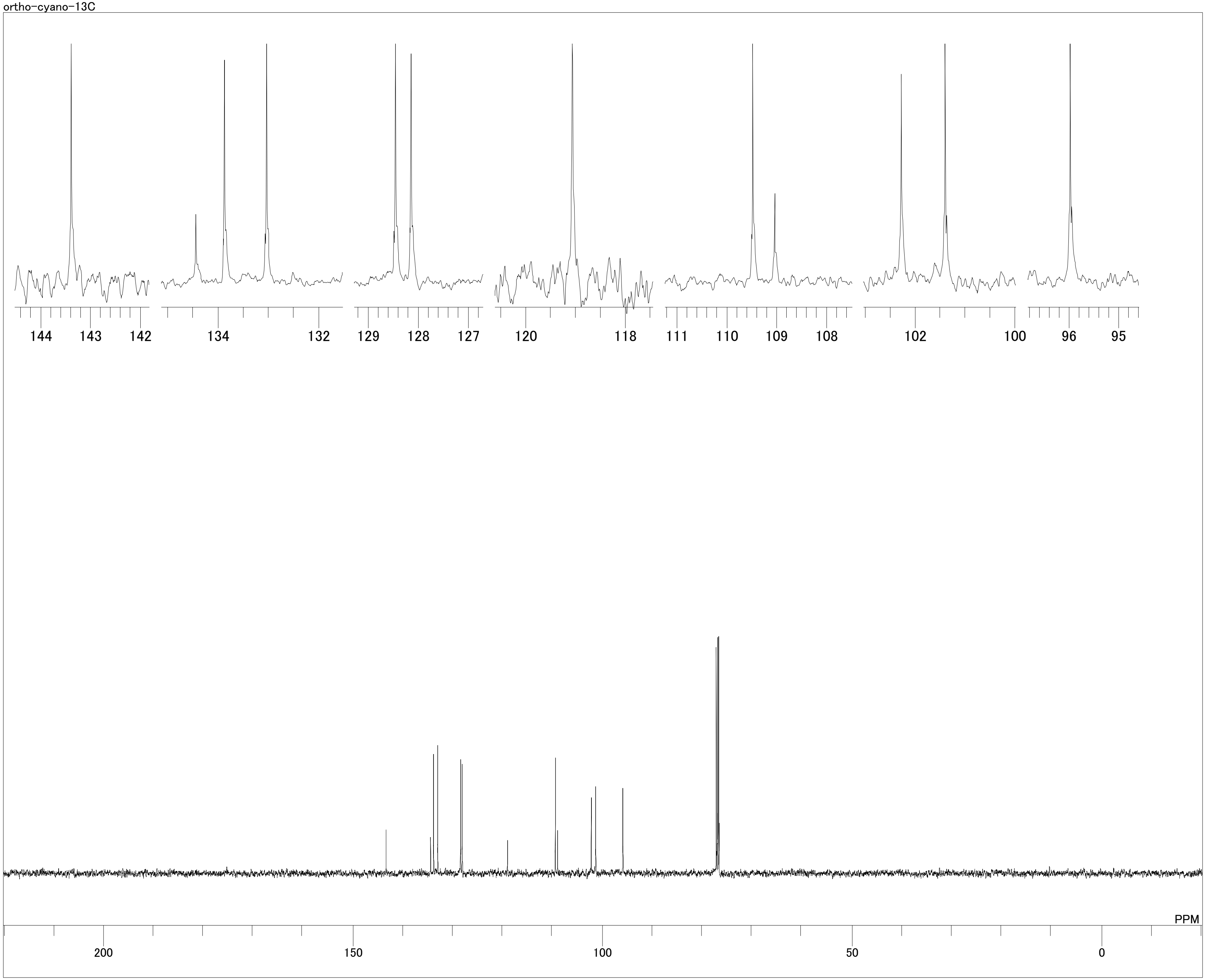

DFILE C:¥Documents and Settings¥All Users¥Doc
COMNT ortho-cyano-13C
DATIM 07-12-2012 22:53:06
OBNUC 13C
EXMOD single_pulse_dec
OBFRQ 125.77 MHz
OBSET 7.87 KHz
OBFIN 4.21 Hz
POINT 32768
FREQU 39308.18 Hz
SCANS 20
ACQTM 0.8336 sec
PD 2.0000 sec
PW1 3.83 usec
IRNUC 1H
CTEMP 20.8 c
SLVNT CDCL3
EXREF 77.00 ppm
BF 1.20 Hz
RGAIN 60

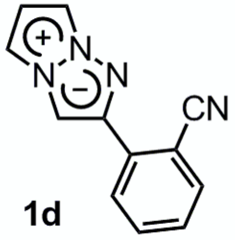

1d

\\Bot025\data\Šw¶\‘åàV\NMR-TAPSu-trans\yellow-1.als
1b-1H

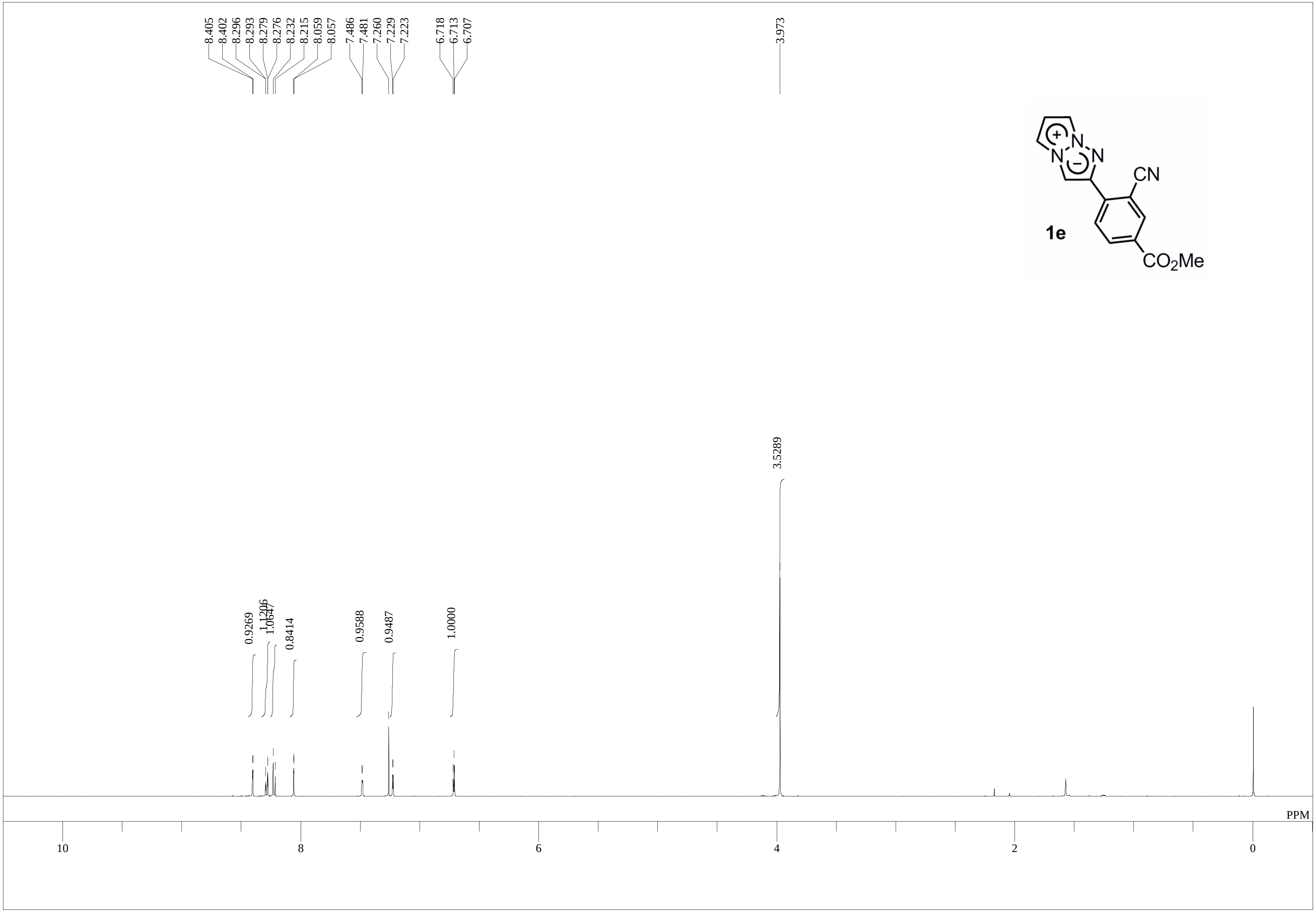

\\Bot025\data\Šw¶\‘åàV\NMR-TAPSu-trans\yellow-13C-1.als
1b-13C

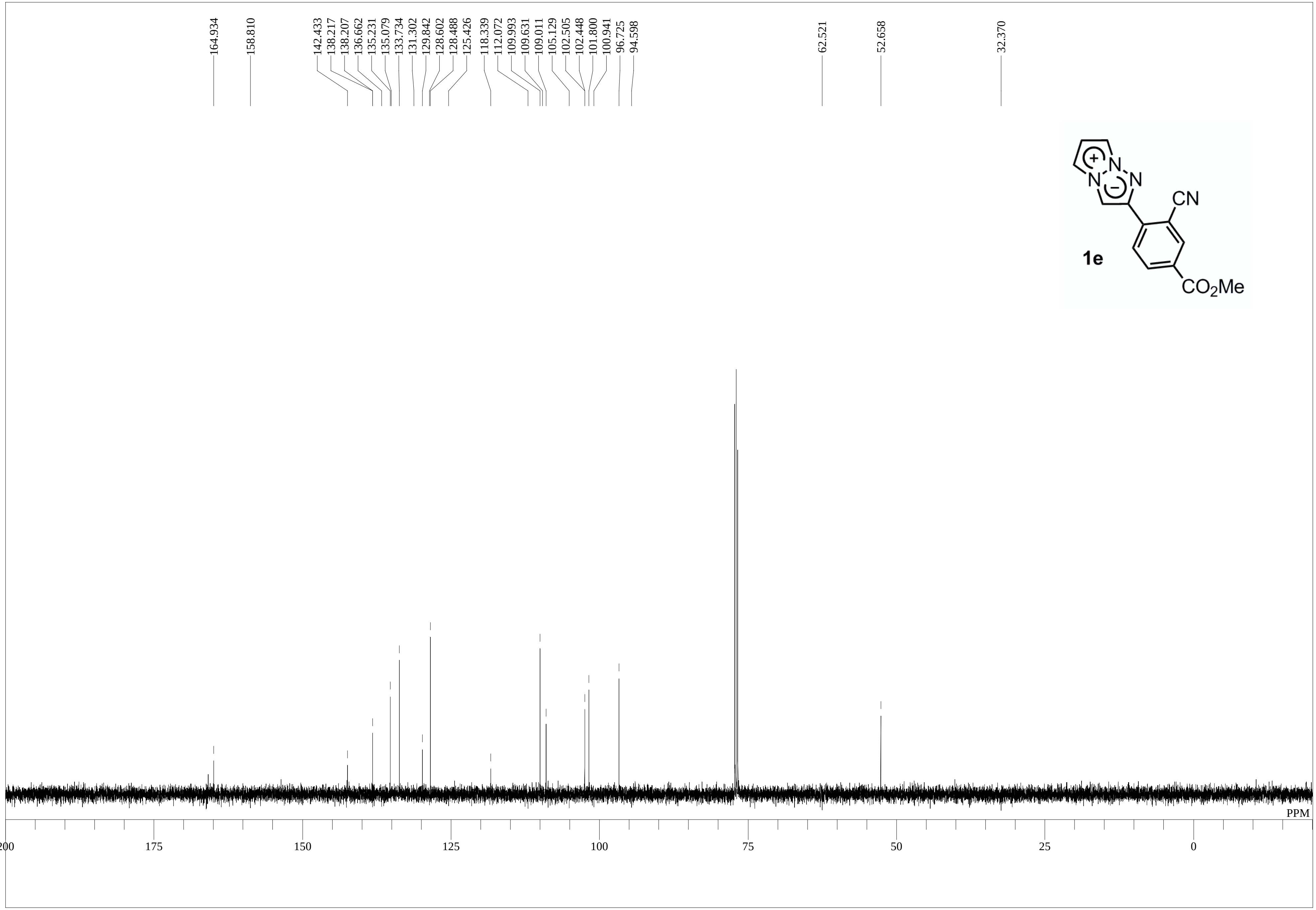

\\Bot025\data\Šw¶\‘åàV\NMR-TAPSu-trans\1249-cc-1.als
1c-1H

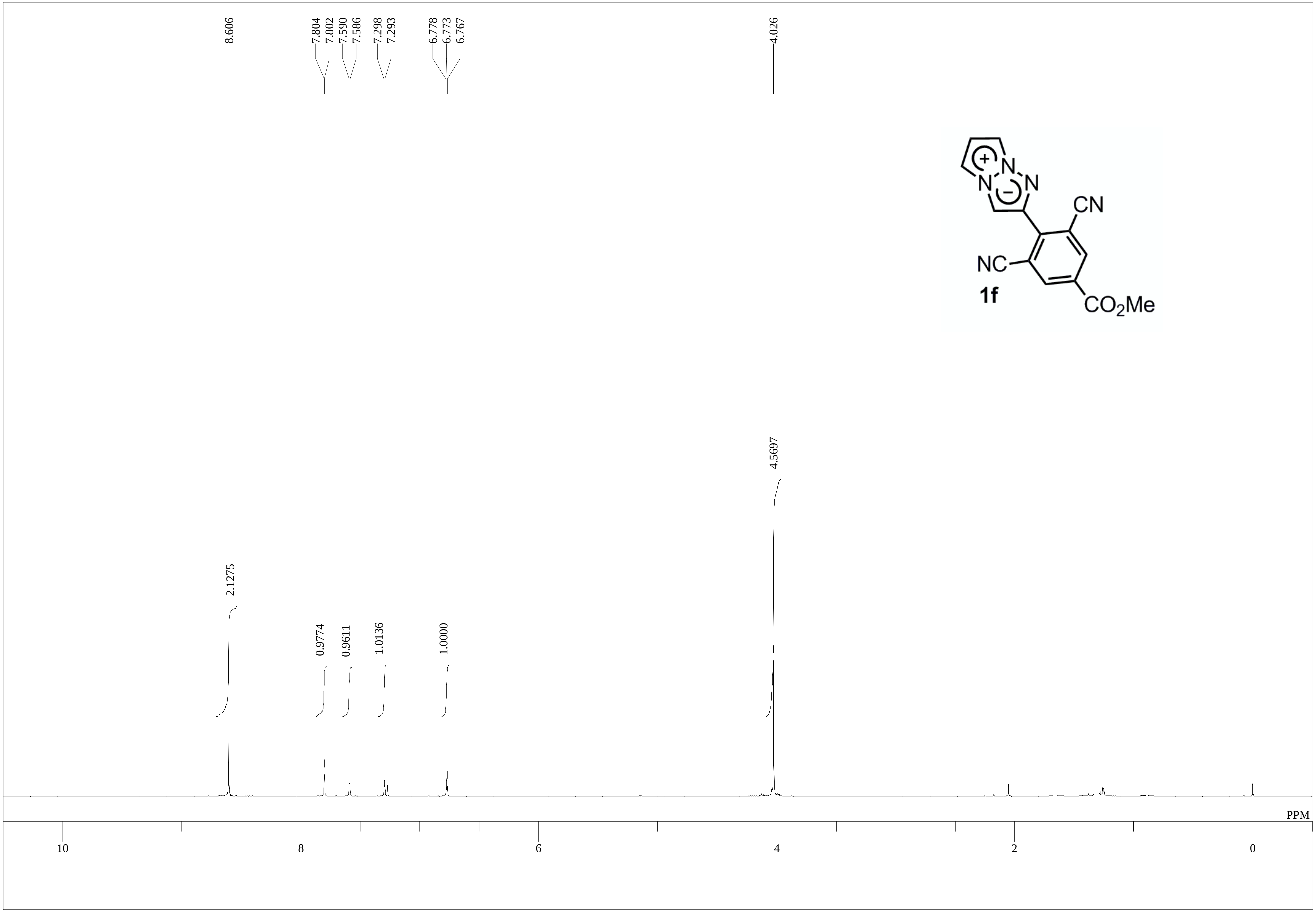

\\Bot025\data\Šw¶\‘ååV\NMR-TAPSu-trans\1249-13C-1.als
1c-13C

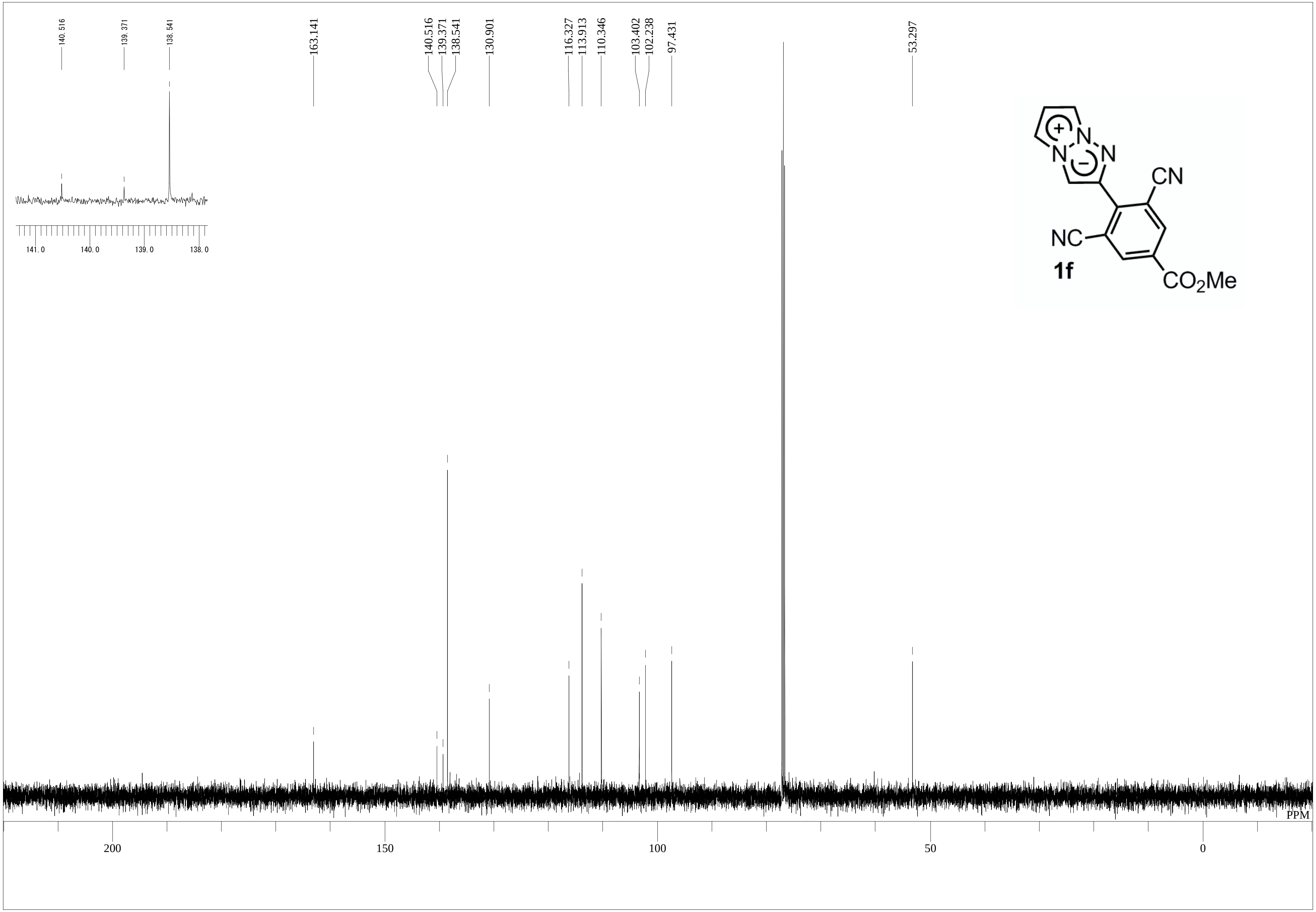

1f

C6H4CO2Me-1H

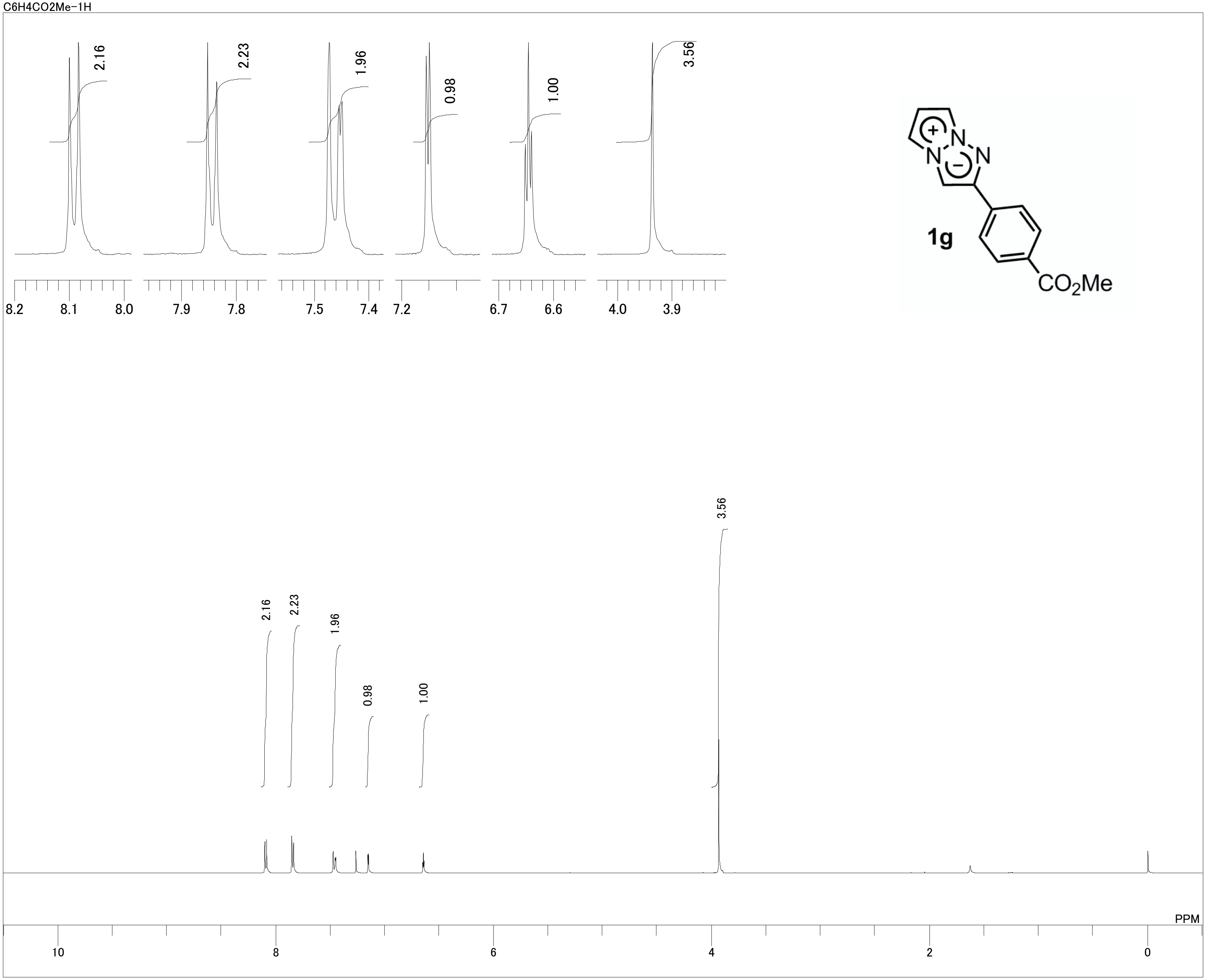

DFILE	C:¥Documents and Settin
COMNT	C6H4CO2Me-1H
DATIM	10-12-2012 17:11:50
OBNUC	1H
EXMOD	single_pulse.ex2
OBFRQ	500.16 MHz
OBSET	2.41 KHz
OBFIN	6.01 Hz
POINT	16384
FREQU	9384.38 Hz
SCANS	5
ACQTM	1.7459 sec
PD	1.0000 sec
PW1	6.50 usec
IRNUC	1H
CTEMP	19.7 c
SLVNT	CDCL3
EXREF	0.00 ppm
BF	0.12 Hz
RGAIN	50

C6H4CO2Me-13C

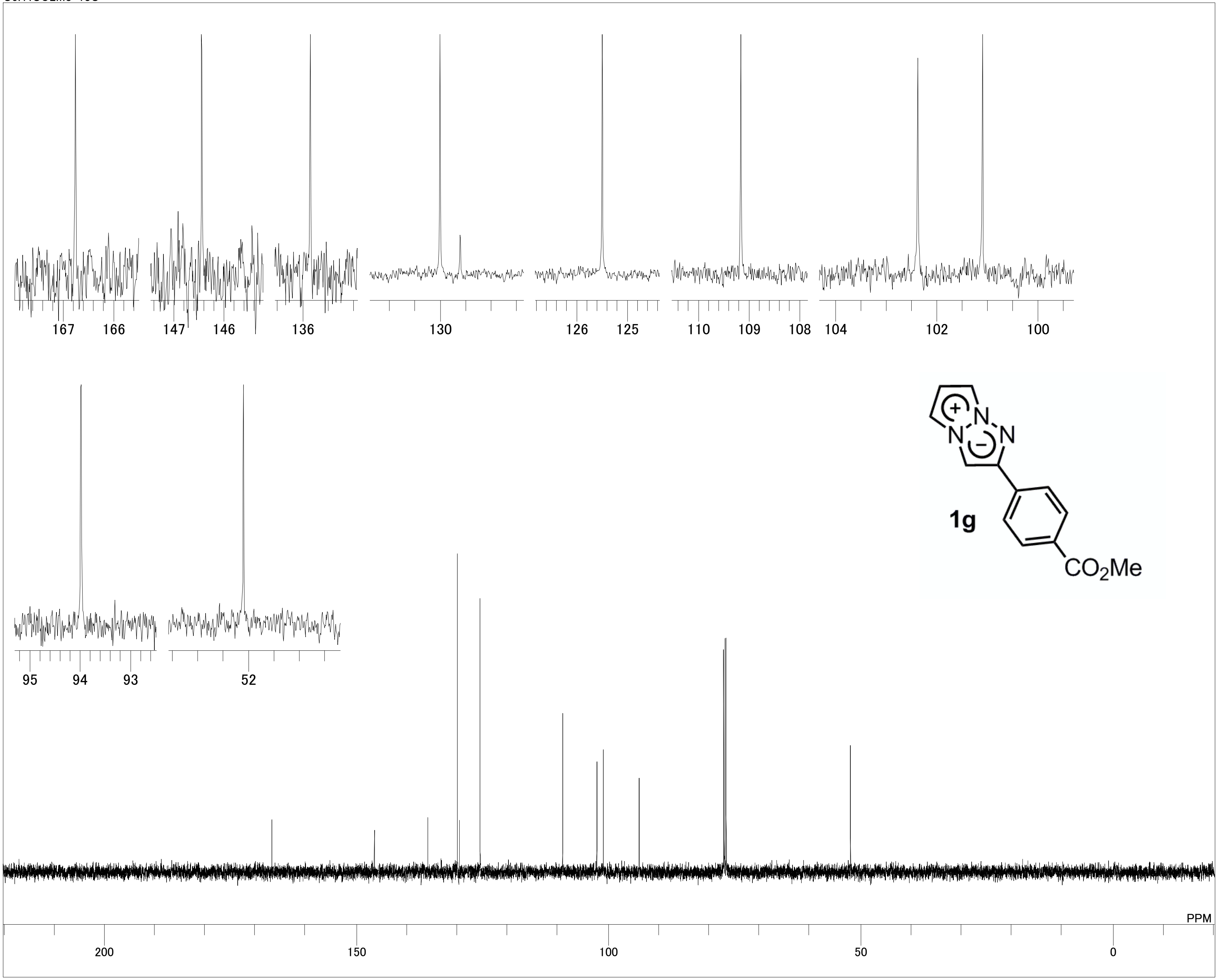

DFILE	C:¥Documents and Settin
COMNT	C6H4CO2Me-13C
DATIM	10-12-2012 19:45:54
OBNUC	13C
EXMOD	single_pulse_dec
OBFRQ	125.77 MHz
OBSET	7.87 KHz
OBFIN	4.21 Hz
POINT	32768
FREQU	39308.18 Hz
SCANS	35
ACQTM	0.8336 sec
PD	2.0000 sec
PW1	3.83 usec
IRNUC	1H
CTEMP	20.0 c
SLVNT	CDCL3
EXREF	77.00 ppm
BF	1.20 Hz
RGAIN	60

\\Bot025\data\Šw¶\‘ååV\NMR-TAPSu-trans\’Ç‰Á\1148-CD2Cl2-1.als
11-1H-CD2Cl2

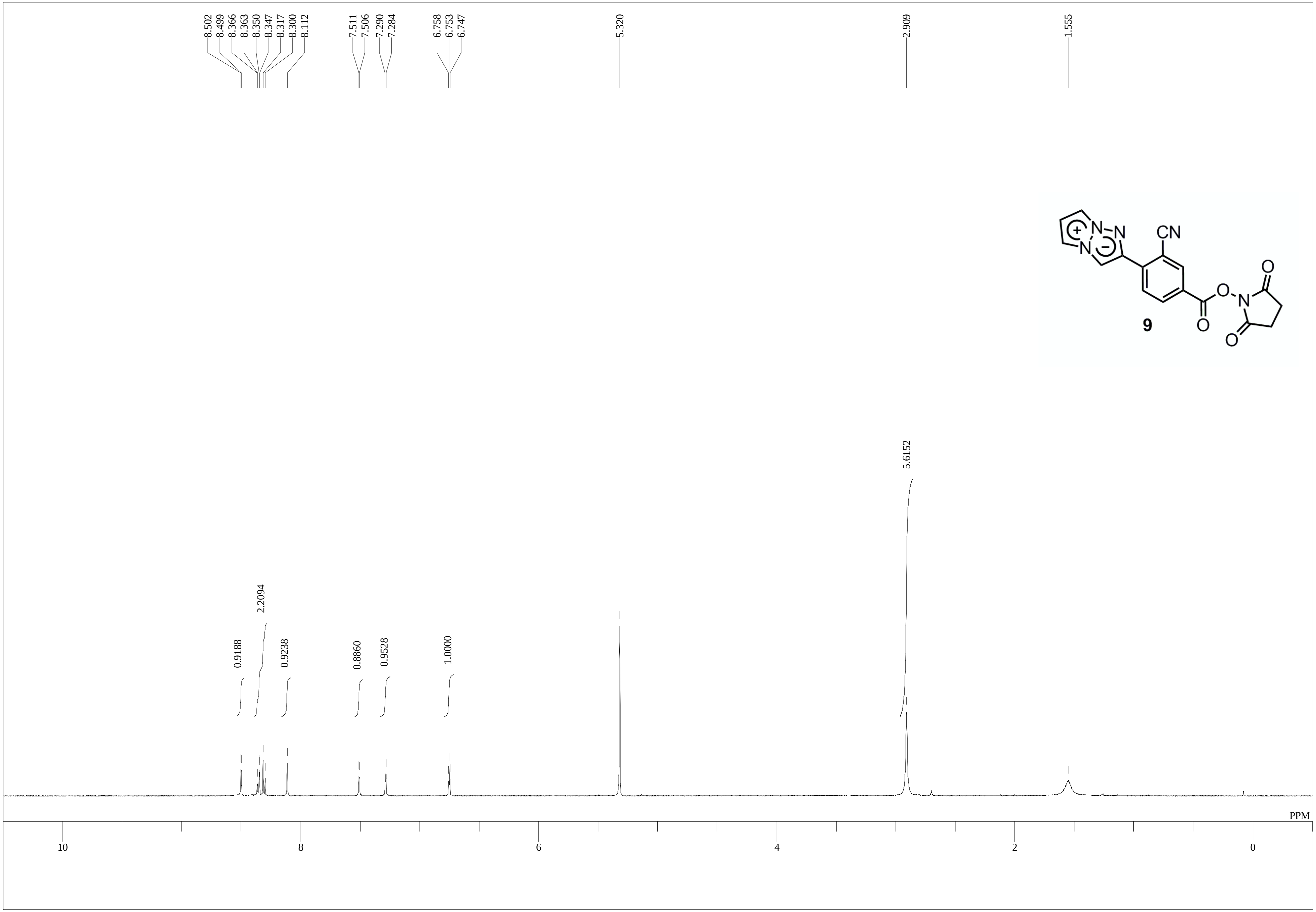

\\Bot025\data\NMR backup\BOT004backup 20130803-20131229\osawa\TAP\TAPSu-data\1277-DMSO-13C.ALS
11-13C

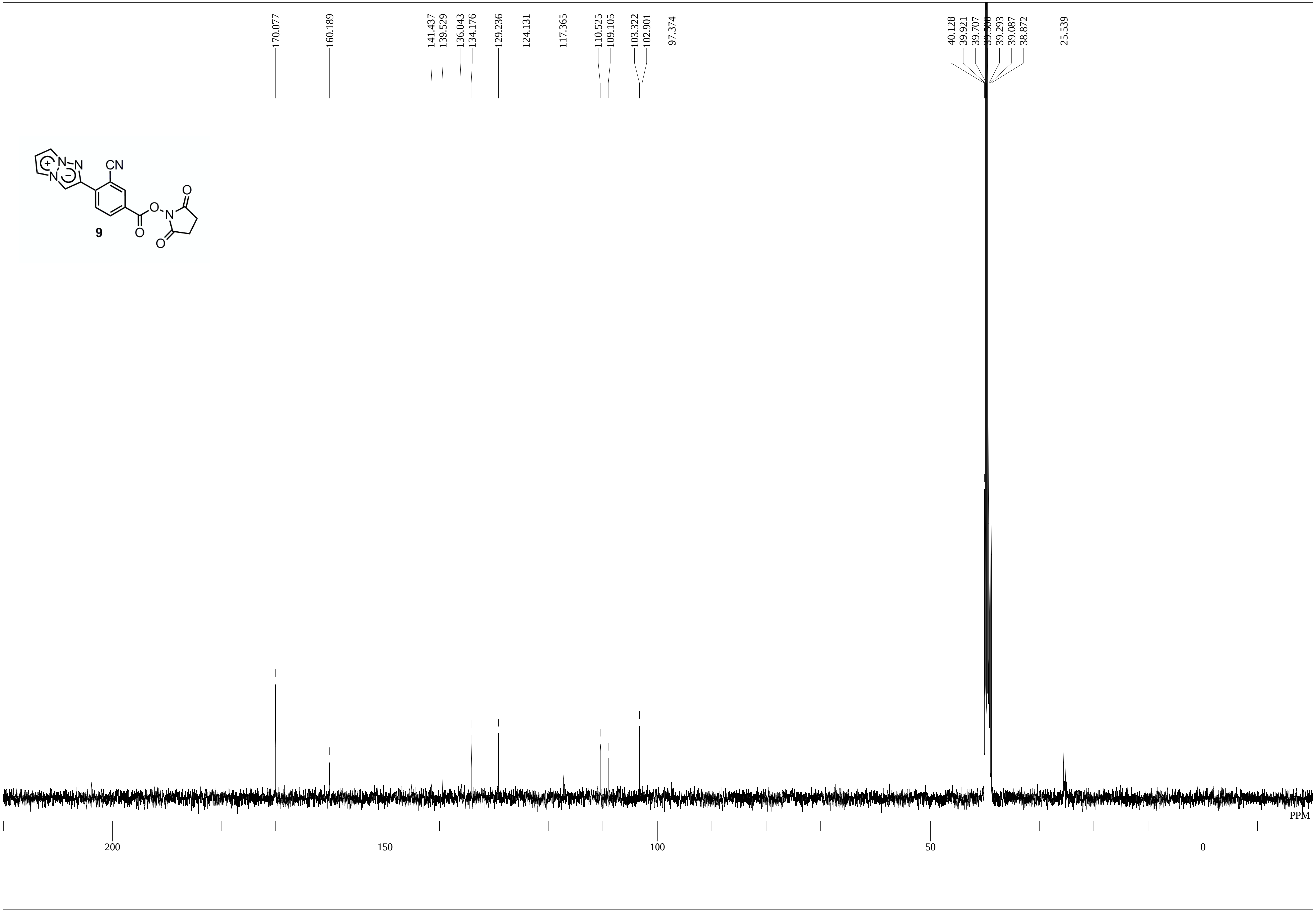

\\Bot025\data\NMR backup\BOT004backup 20130803-20131229\osawa\TAP\1289-cc.ALS
12-1H

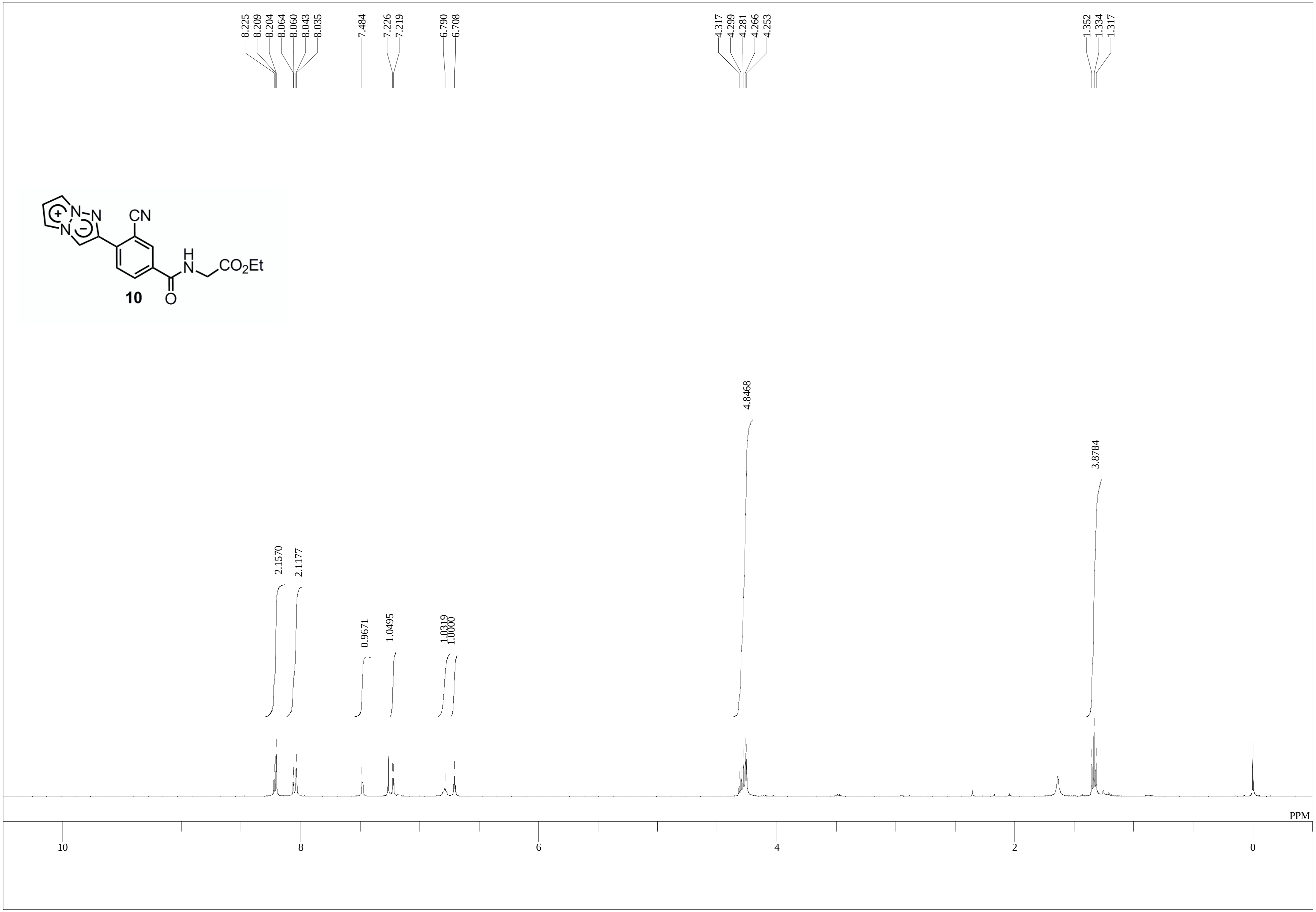

\\Bot025\data\NMR backup\BOT004backup 20130803-20131229\osawa\TAP\TAPSu-data\1289-13C.ALS
12-13C

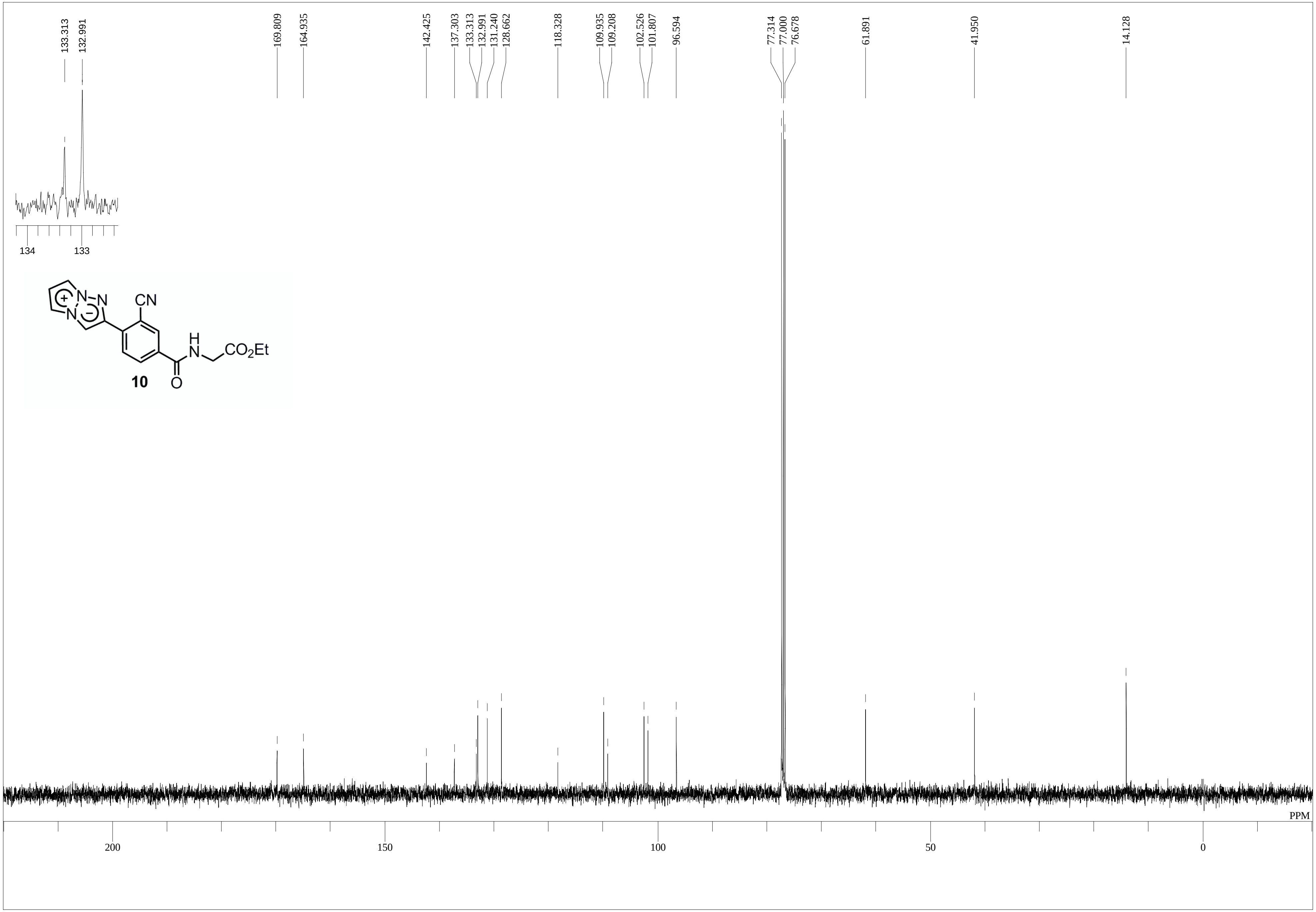

\\150.59.84.6\user\004BOT\osawa\TAP\tap-tripeptide-3.ALS
13-1H

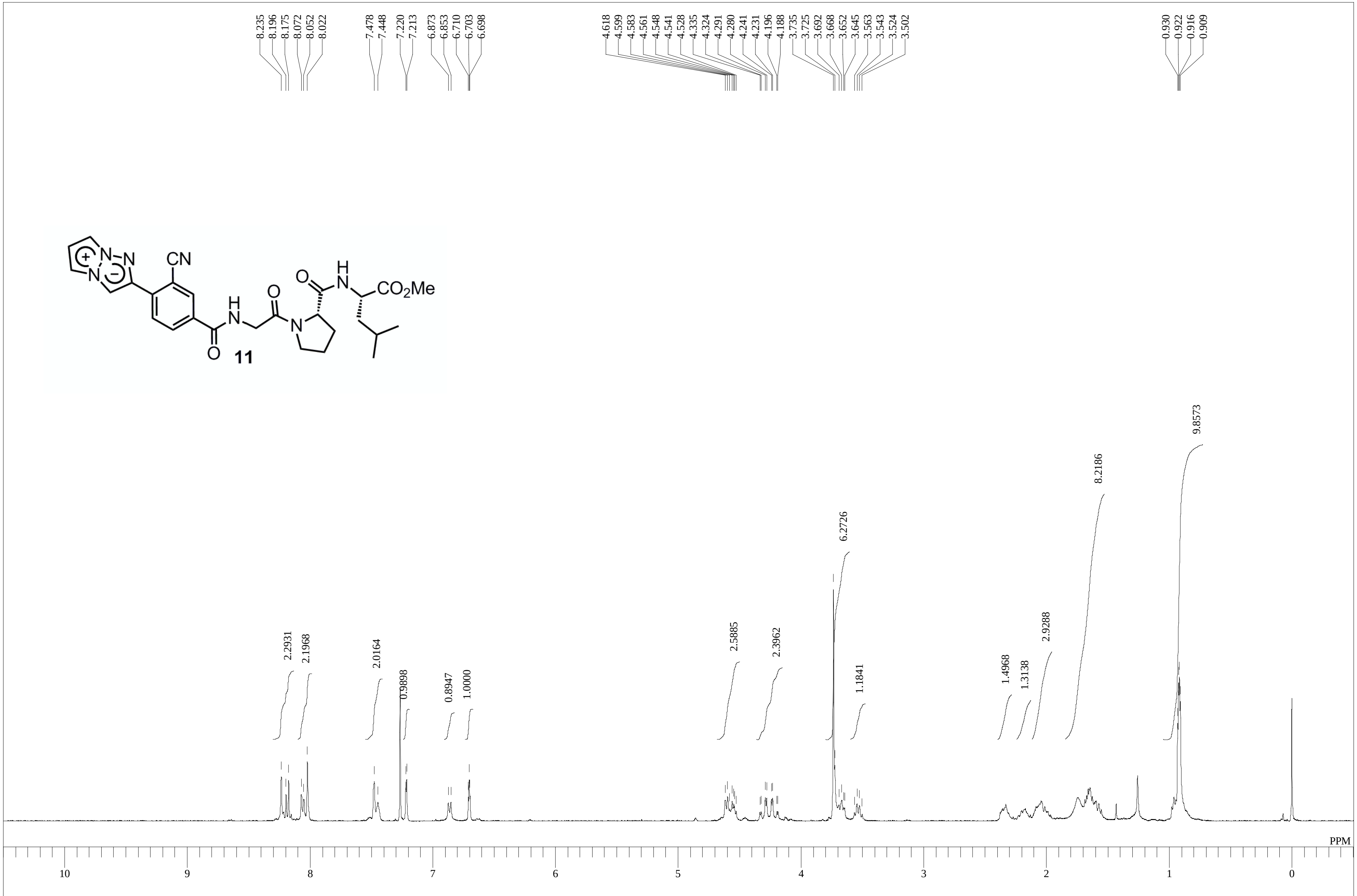

\\150.59.84.6\user\004BOT\osawa\TAP\tap-tripeptide-4-13C.ALS
13-13C

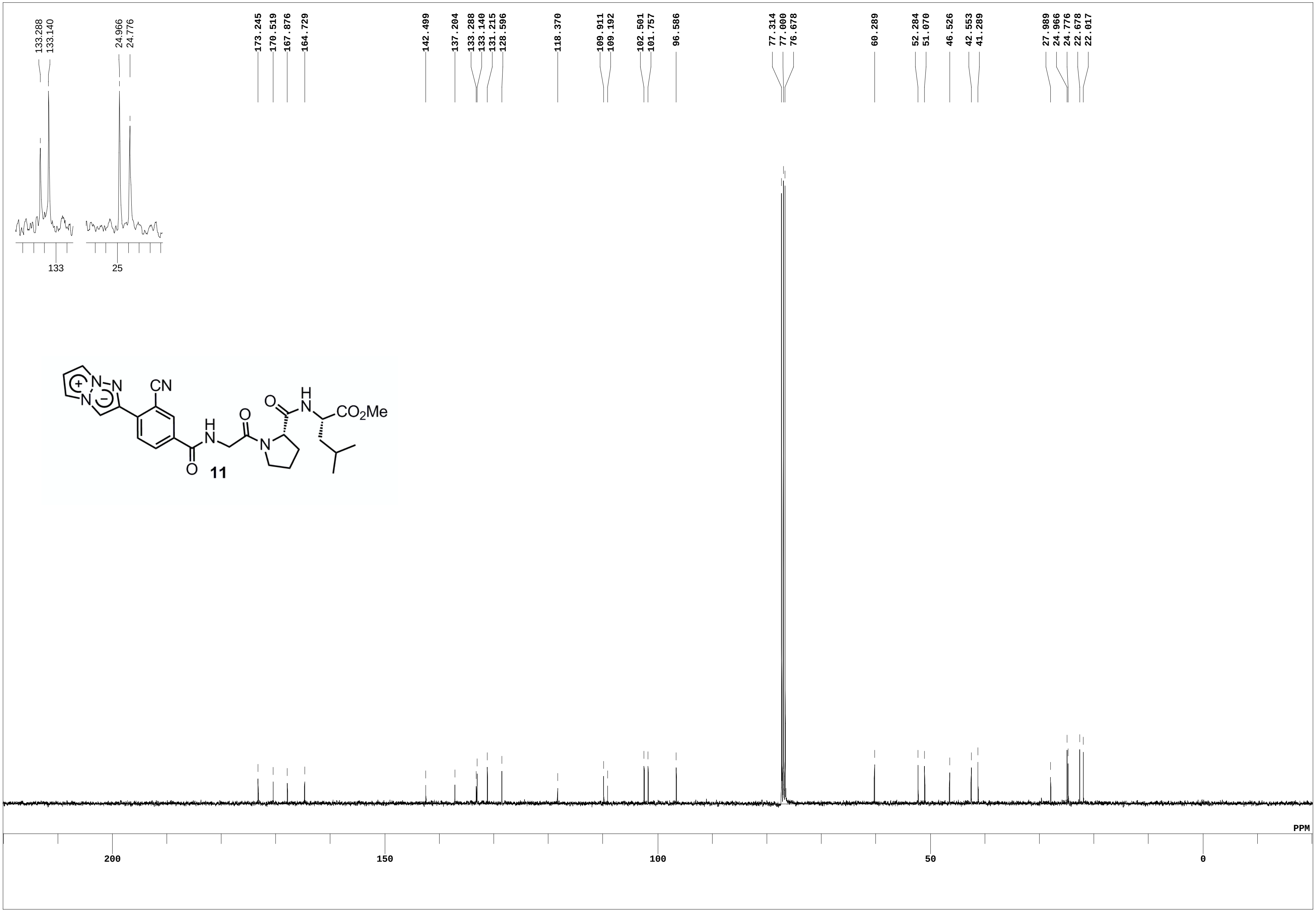

\\Bot025\data\NMR backup\BOT004backup 20130803-20131229\osawa\TAP\1281-cc2.ALS
tripeptide-1H

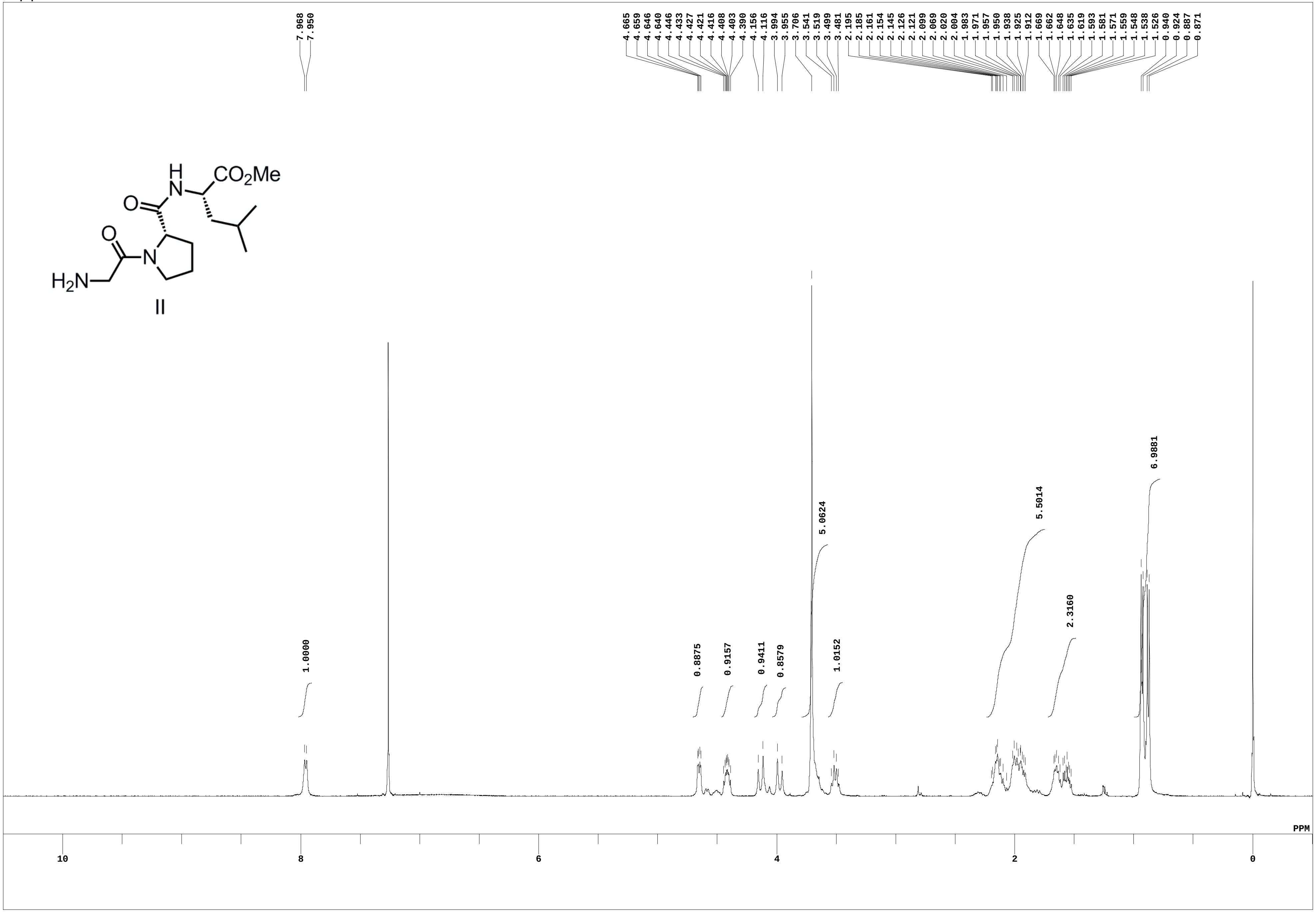

\\Bot025\data\NMR backup\BOT004backup 20130803-20131229\osawa\TAP\1281-13C-2.ALS
tripeptide-13C

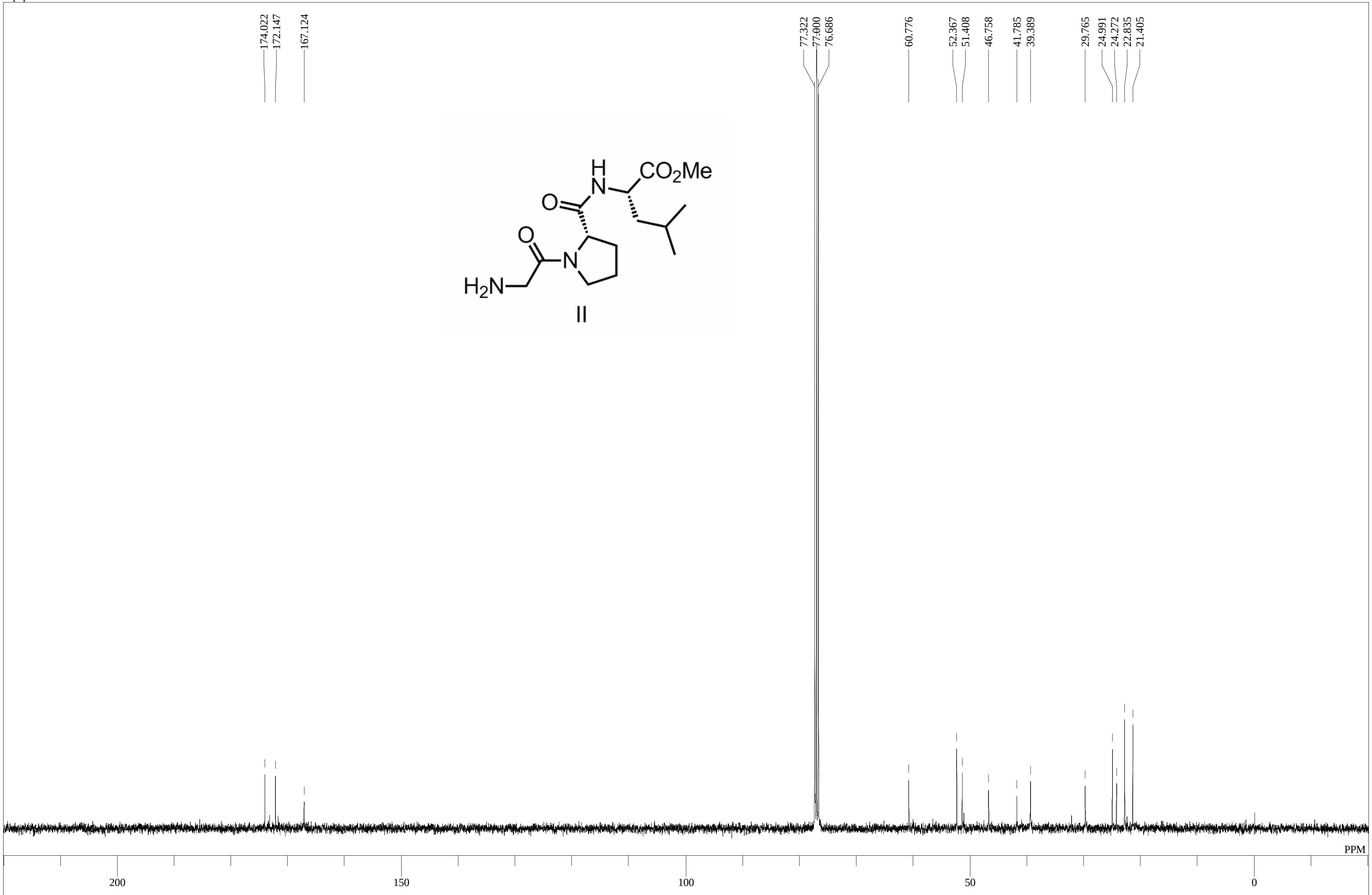

\\150.59.84.6\user\004BOT\osawa\TAP\4-ph-ester-1H-2.als

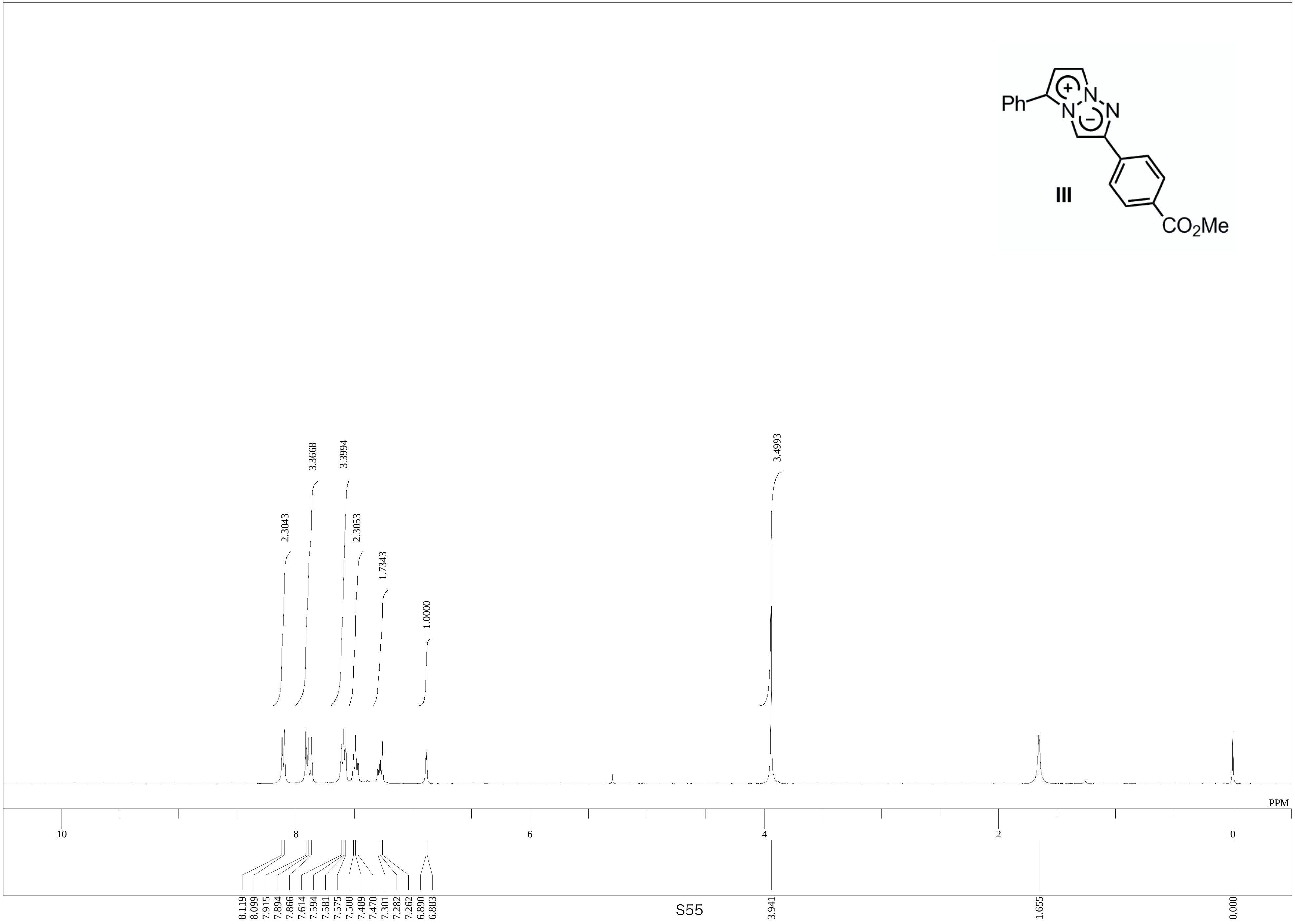

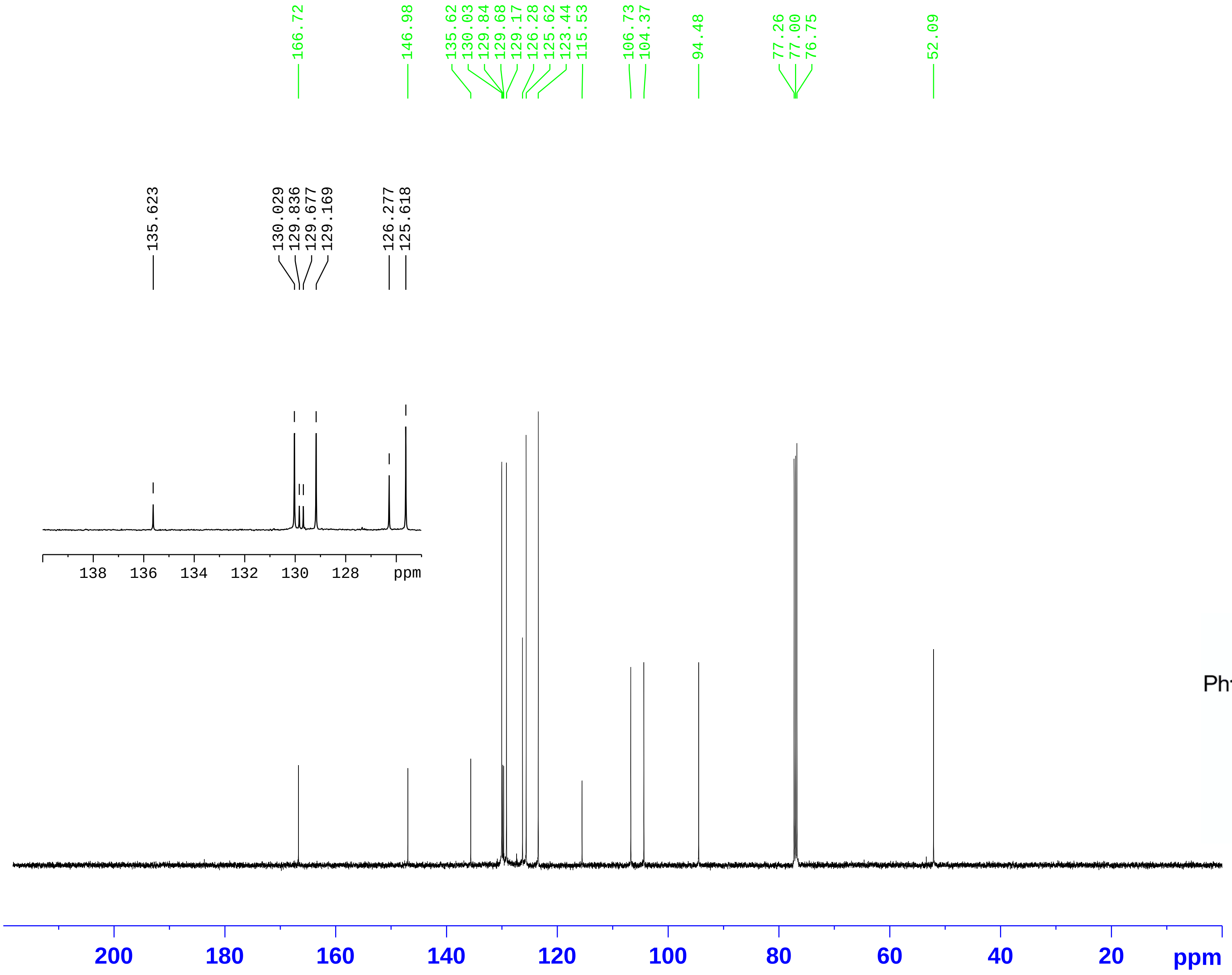


```
Current Data Parameters
NAME     osawa-1311-13C
EXPNO                10
PROCNO                1

F2 - Acquisition Parameters
Date_          20140826
Time              19.30
INSTRUM           spect
PROBHD   5 mm PABBO BB-
PULPROG          zgpg30
TD                65536
SOLVENT           CDCl3
NS                  512
DS                    4
SWH           29761.904 Hz
FIDRES         0.454131 Hz
AQ            1.1010548 sec
RG                  203
DW               16.800 usec
DE                 6.50 usec
TE                301.5 K
D1           2.00000000 sec
D11          0.03000000 sec
TD0                   1

======== CHANNEL f1 ========
NUC1                13C
P1                11.00 usec
PL1                1.00 dB
PL1W        80.20954132 W
SFO1        125.7728791 MHz

======== CHANNEL f2 ========
CPDPRG2         waltz16
NUC2                 1H
PCPD2             80.00 usec
PL2                1.00 dB
PL12              17.77 dB
PL13              18.00 dB
PL2W        19.45380783 W
PL12W        0.40926495 W
PL13W        0.38815448 W
SFO2        500.1420006 MHz

F2 - Processing parameters
SI                32768
SF          125.7603104 MHz
WDW                  EM
SSB      0
LB                 1.00 Hz
GB       0
PC                 1.40
```

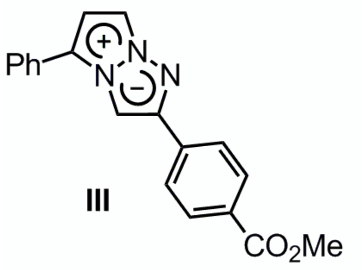

III

\\150.59.84.6\user\004BOT\osawa\TAP\4-ph-cnester.ALS

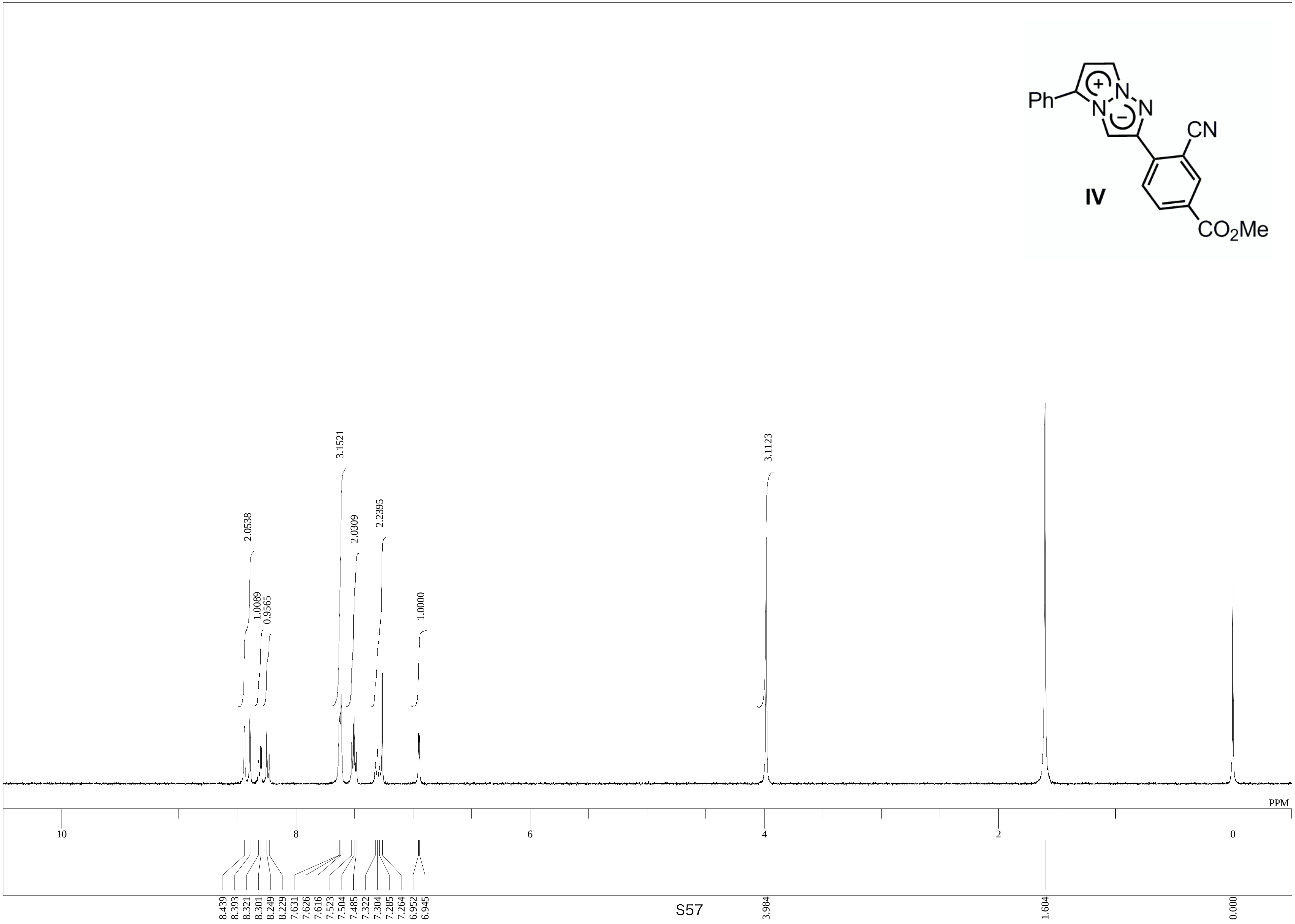

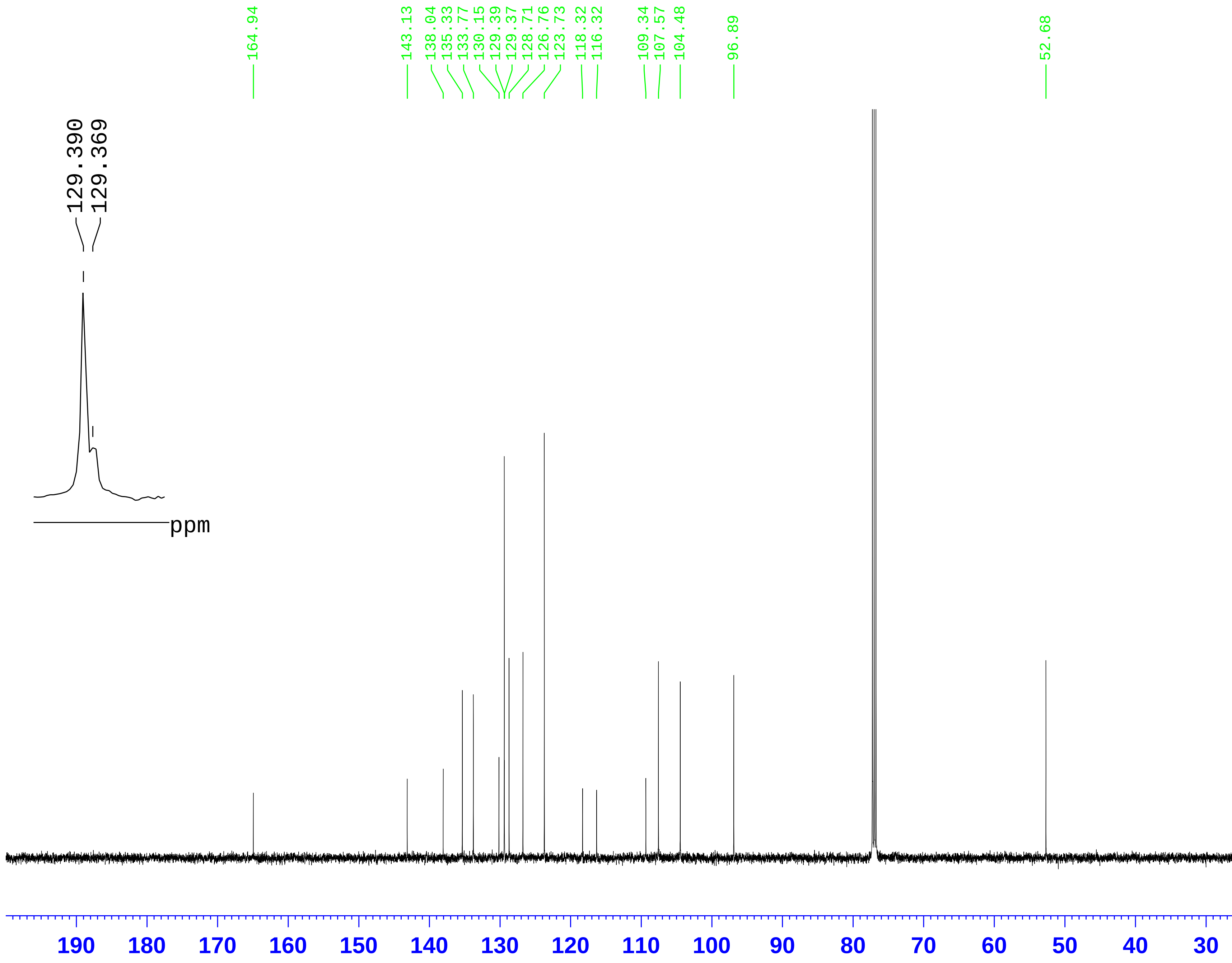


```
Current Data Parameters
NAME      osawa-1304-13C
EXPNO                 10
PROCNO                 1

F2 - Acquisition Parameters
Date_           20140826
Time               22.23
INSTRUM            spect
PROBHD   5 mm PABBO BB-
PULPROG          zgpg30
TD                 65536
SOLVENT            CDCl3
NS                  1024
DS                     4
SWH            29761.904 Hz
FIDRES          0.454131 Hz
AQ             1.1010548 sec
RG                   203
DW                16.800 usec
DE                  6.50 usec
TE                 301.3 K
D1            2.00000000 sec
D11           0.03000000 sec
TD0                    1

======== CHANNEL f1 ========
NUC1                 13C
P1                 11.00 usec
PL1                 1.00 dB
PL1W         80.20954132 W
SFO1         125.7728791 MHz

======== CHANNEL f2 ========
CPDPRG2          waltz16
NUC2                  1H
PCPD2              80.00 usec
PL2                 1.00 dB
PL12               17.77 dB
PL13               18.00 dB
PL2W         19.45380783 W
PL12W         0.40926495 W
PL13W         0.38815448 W
SFO2         500.1420006 MHz

F2 - Processing parameters
SI                 32768
SF           125.7603030 MHz
WDW                   EM
SSB      0
LB                  1.00 Hz
GB       0
PC                  1.40
```

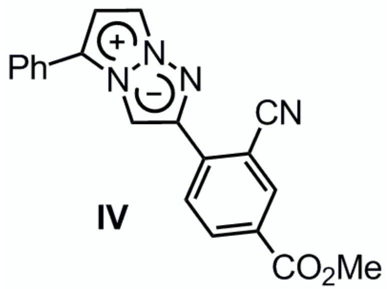

Supplement: Supplementary file 1 [file SC-006-C4SC02780A-s001.pdf]
